# Recent Advances on Synthetic Methodology Merging C–H Functionalization and C–C Cleavage

**DOI:** 10.3390/molecules25245900

**Published:** 2020-12-13

**Authors:** Hamid Azizollahi, José-Antonio García-López

**Affiliations:** 1 Department of Chemistry, Faculty of Science, Ferdowsi University of Mashhad, Mashhad 91775-1436, Iran; 2 Grupo de Química Organometálica, Campus de Espinardo, Universidad de Murcia, 30100 Murcia, Spain

**Keywords:** C–H functionalization, C–H activation, C–C cleavage, synthetic methodology, transition-metal catalysis, photocatalysis

## Abstract

The functionalization of C–H bonds has become a major thread of research in organic synthesis that can be assessed from different angles, for instance depending on the type of catalyst employed or the overall transformation that is carried out. This review compiles recent progress in synthetic methodology that merges the functionalization of C–H bonds along with the cleavage of C–C bonds, either in intra- or intermolecular fashion. The manuscript is organized in two main sections according to the type of substrate in which the cleavage of the C–C bond takes place, basically attending to the scission of strained or unstrained C–C bonds. Furthermore, the related research works have been grouped on the basis of the mechanistic aspects of the different transformations that are carried out, i.e.,: (a) classic transition metal catalysis where organometallic intermediates are involved; (b) processes occurring via radical intermediates generated through the use of radical initiators or photochemically; and (c) reactions that are catalyzed or mediated by suitable Lewis or Brønsted acid or bases, where molecular rearrangements take place. Thus, throughout the review a wide range of synthetic approaches show that the combination of C–H and C–C cleavage in single synthetic operations can serve as a platform to achieve complex molecular skeletons in a straightforward manner, among them interesting carbo- and heterocyclic scaffolds.

## 1. Introduction

The fields of C–H activation/functionalization have become well-established areas in organic synthesis, surging as reliable and powerful tools to achieve a wide range of transformations [1,2,3,4,5,6,7]. The past twenty years have witnessed a great evolution in the approach to perform the functionalization of C–H bonds, enhancing its applicability [8]. Nowadays, there is a growing interest to extend the frontiers of the C–H functionalization field through its merging with other aspects of synthesis, for instance electrochemical [9,10,11]; and photochemical processes [12,13], cascade reactions [14,15], or functionalization of remote positions [16,17,18,19], among others. Parallel to the research on C–H functionalization, the topic of C–C bond cleavage has experienced an exponential development in recent times [20,21,22,23,24,25]. Remarkably, the consideration of ubiquitous C–C bonds as functional groups opens up enormous possibilities for synthesis, generating novel disconnections in retrosynthesis previously thought inaccessible and unveiling new building blocks. In this regard, chemists have tackled the scission of C–C bonds with different hybridization and taken advantage of some features of carbon skeletons such as the favorable release of the strain in small carbocyclic rings [26]. The merge of C–H/C–C bond cleavage can potentially give rise to a vast array of methods that allow straightforward routes to the synthesis of complex organic skeletons, including relevant heterocyclic scaffolds.

This review will focus on the recent advances of synthetic methodology involving transformations where both a C–H and a C–C bond are cleaved in the overall reaction. We have considered those research works where the C–H bond cleavage occurs either through the formation of carbon–metal bonds (C–H activation) or through other processes, for example via the homolytic C–H bond scission. In addition, we have included some interesting reactions where the cleavage of a C–H bond is used as a tool to achieve reactive intermediates that are further transformed, even though when that specific C–H bond is restored in the final organic product arising from the general reaction with apparent no derivatization.

In early 2017 Marek and co-workers reported in a nice review on the C–H/C–C functionalization topic [27], collecting the relevant progress made until that date. Given the dynamism of this field, a considerable amount of work has been carried out in this specific area within the last years, which we have tried to summarize and present in this manuscript. This review focuses on the research made in the field of transition metal-catalysis after the publication of Marek’s revision. Nevertheless, we have not included reactions where the C–C cleavage proceeds via decarboxylative [28,29]/decarbonylative [30] processes or through the use of 2-norbornene auxiliary ligands (Catellani approach) [31,32,33], since these topics have been reviewed recently. We have, however, taken into account those synthetic methods based on the generation of radical or polar intermediates published in the last five years.

We have grouped the different reactions according to the type of substrate in which the C–C scission takes place, basically regarding the two main approaches where either strained or unstrained C–C bonds are cleaved. Within each class there are some sub-groups depending on the specific methodology employed to merge C–H/C–C cleavage. For instance: (a) methods using metal mediated or catalyzed reactions occurring through organometallic intermediates; (b) processes involving radical intermediates, generated either photochemically or thermally; and (c) other methods relying on the use Lewis or Brønsted acid or base as key feature to carry out the reaction.

Furthermore, in order to facilitate the comprehension to the non-specialized reader we have focused the discussion on the mechanistic aspects underneath theses reactions where a series of elementary steps lead to overall complex transformations. We have highlighted the C–H and the C–C bonds that are cleaved during the reaction in red and blue color respectively. Similarly, the new C–C or C–heteroatoms bonds formed are remarked in bold black color.

## 2. Synthetic Methodology Involving C–H Functionalization Along with the Cleavage of Strained C–C Bonds

As mentioned in the introductory section of this review, the release of the strain of carbocyclic structures is one of the driving forces that can promote the cleavage of C–C bonds [26], generating intermediates of different nature depending on the experimental conditions which can further evolve intra- or intermolecularly to afford new organic scaffolds. This section presents recent progress carried out on the different approaches where the functionalization of a C–H bond is achieved through the use of cyclopropyl and cyclobutyl scaffolds [34,35,36,37,38,39,40,41].

### 2.1. Reactions Involving Transition-Metal Catalyzed or Mediated Processes and Strained Substrates

Transition-metal catalysis occupies a privileged position in the development of new synthetic methodologies given its enormous versatility in terms of bond activation and bond formation processes, and therefore they have been used extensively to perform either the functionalization of C–H or C–C bonds [23,42,43]. Nevertheless, in some cases these two types of bonds can be activated in a single synthetic operation. In this section, recent examples show the use of precious- or base-metals, or a combination of both, to achieve interesting organic transformations.

**Scheme 1:**
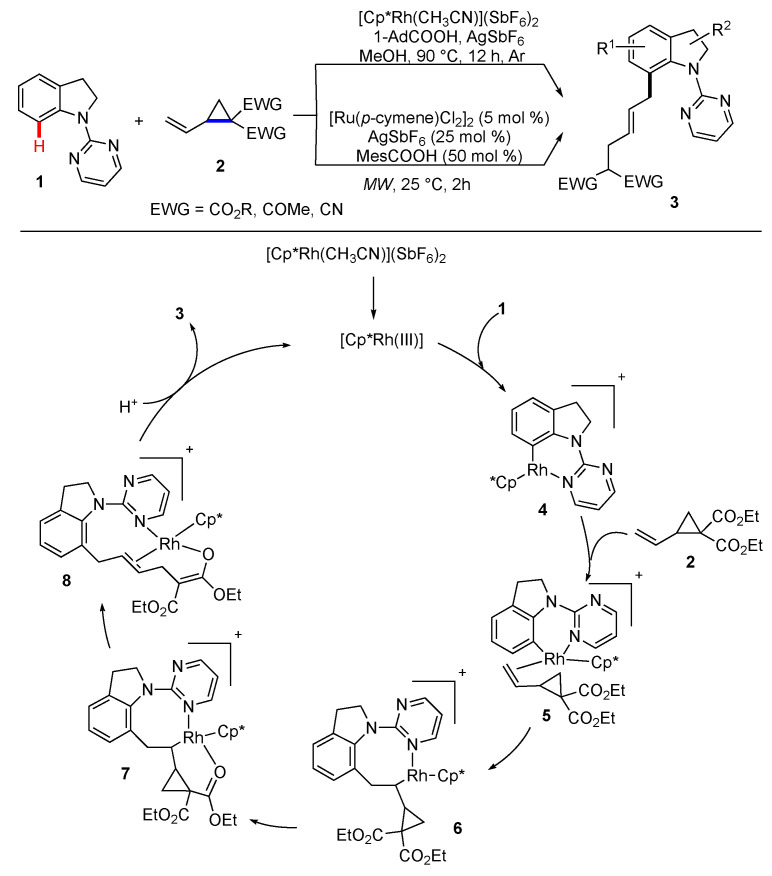
Rh-catalyzed functionalization of indolines. Cp* refers to pentamethylcyclopentadienyl ligand.

The direct C7 allylation of indolines via sequential C–H and C–C activation catalyzed by Rh(III) was reported by Song and co-workers (Scheme 1) [44]. A pyrimidine ring bonded to the nitrogen atom of the indoline core was used as directing group to promote the C7–H metalation of substrates **1**. Coordination and migratory insertion of a substituted vinylcyclopropane (VCP) led to the formation of an eight-membered rhodacycle **6**. The intramolecular coordination of one of the ester groups in the intermediate **7** would facilitate the C–C cleavage of the cyclopropane fragment to give a coordinated enolate **8**. Subsequent protonation would render the desired C7-allylated indoline and restore the active Rh(III) species. The optimal reaction conditions included 20 mol% of AgSbF_6_ in order to form cationic Rh(III) species in situ and 30 mol% of 1-adamantane carboxylic acid as an additive to promote the metalation step. Further work on this reaction pointed to the possibility to carry out this transformation at room temperature in solvent-free and microwave-assisted conditions through Ru-catalysis [45].

**Scheme 2:**
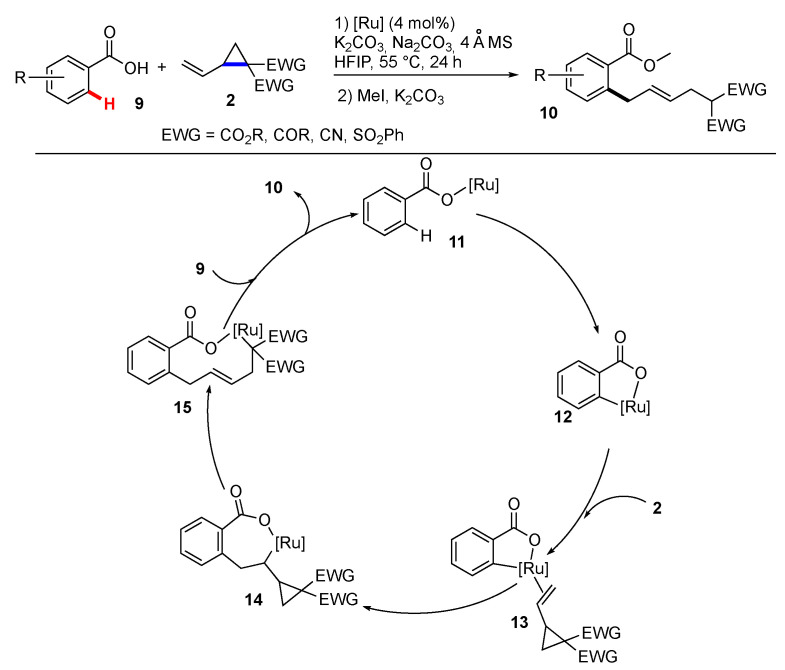
Ru-catalyzed allylation of benzoic acids.

The Gooßen group exploited the favorable scission of the strained C–C bond inherent to vinylcyclopropenes to form *ortho*-allylated benzoic acids and esters **10** (Scheme 2) [46]. The use of a catalytic amount of [Ru(*p*-cymene)Cl_2_]_2_ in the presence of a base and HFIP solvent allowed the smooth metalation of the *ortho*-C–H bond of benzoic acid derivatives. As in other reactions where VCPs are used as coupling partners, the insertion of the olefine in the Ru–C bond was followed by C–C bond cleavage and protodemetalation. The coupling products were isolated as methyl esters upon an in situ methylation step. The scope of the reaction revealed the compatibility of the reaction conditions with the halogen substituents on the aromatic ring. The VCPs **2**, however, must bear two electron-withdrawing groups (such as two ester substituents) to allow the C–C bond cleavage to proceed. The yield and *E/Z* ratio of the allylated products **10** depended inversely on the bulkiness of the ester groups, with the bulkier ones giving lower yields but superior regioselectivities.

**Scheme 3:**
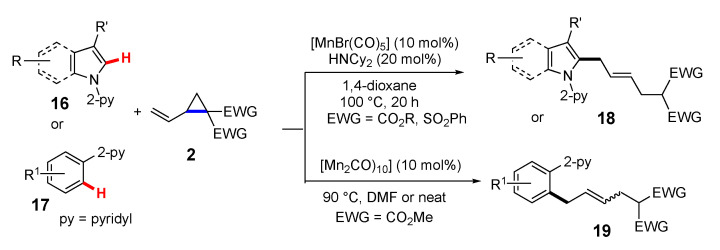
Mn-catalyzed allylation of 2-pyrimidyl arenes and heteroarenes.

Following up on the interest to apply base-metal catalysts to the C–H activation field [47], Ackermann showed the possibility to carry out the allylation of indoles via C–H/C–C cleavage thorough the use of vinylcyclopropenes and a Co(III) catalyst [48]. Soon thereafter, both Ackermann’s and Glorius’ groups reported nearly simultaneously the use of Mn species to carry out this transformation (Scheme 3) [49,50]. Both protocols employed substrates bearing a 2-pyridyl directing group to promote the C–H metalation. The insertion of the terminal C=C double present in the vinylcyclopropene coupling partner, followed by β-carbon elimination and protodemetalation would afford the allylated products **18** or **19** as a *E/Z* mixture of isomers. The *E/Z* selectivity was affected by the conditions. When the reaction was carried out in concentrated DMF solution or neat conditions using [Mn_2_(CO)_10_] as the catalyst, *E/Z* mixtures ranging from 2.1:1 to 4.7:1 were obtained. The *E*-diastereoselectivity was improved by the use of [MnBr(CO)_5_] in the presence of HNCy_2_, in 1,4-dioxane at 100 °C, reaching *E/Z* ratios from 4:1 to 10:1. These last conditions were suitable to perform the 2-allylation of tryptophan derivatives precluding the racemization of the chiral center. Several mechanistic experiments were carried out to outline the characteristics features of this reaction. A possible radical pathway could be discarded since the reaction proceeded smoothly in the presence of radical scavengers. The kinetic isotopic effect had a value close to 1, indicating that the C–H activation was not the rate limiting step. In addition, DFT calculations showed that the formation of *E* isomer was favoured due to the easier coordination of the ester group (present in the starting VCP) to the metal in the transition state involved in the C–C cleavage step.

**Scheme 4:**
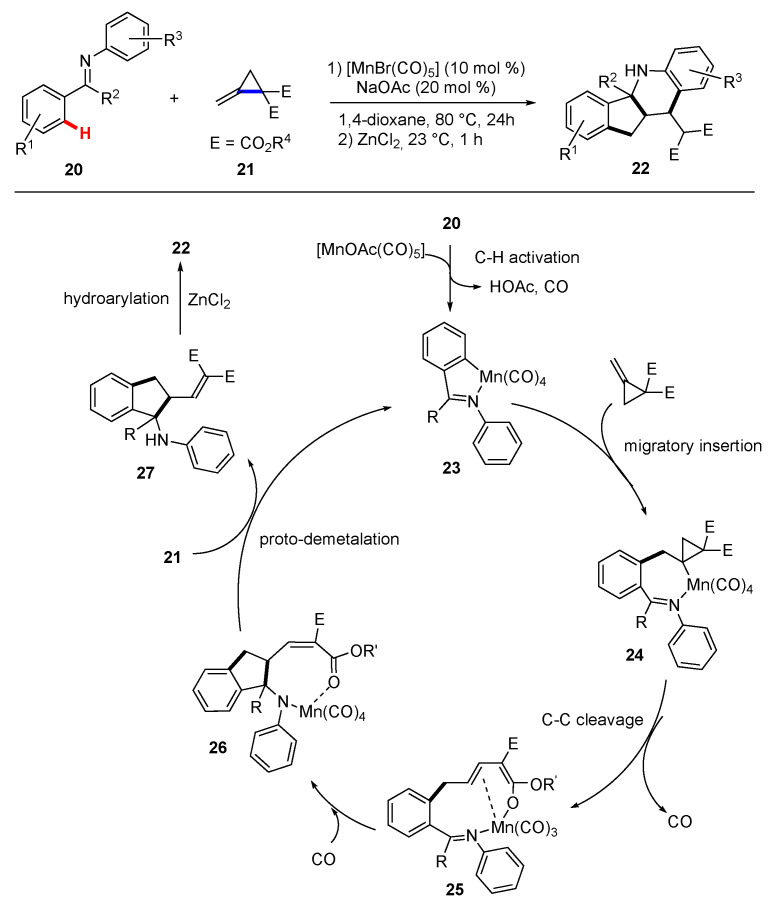
Mn-catalyzed functionalization of imines.

In 2017, Ackermann described the use of sustainable Mn catalysis to perform the C–H/C–C activation process taking place in the coupling of imines **20** and methylenecyclopropanes (MCPs) (Scheme 4) [51]. In this case several Mn(I) complexes, and particularly [MnBr(CO)_5_], were able to successfully deliver the coupling product, overpassing the performance of other transition metal catalyst based on Ru, Rh or Pd. The proposed mechanism is initiated by the coordination of the imine to Mn(I) followed by acetate-assisted C–H activation. The aryl-Mn intermediate **23** evolves through migratory insertion of the terminal olefin present in the MCP coupling partner to give the intermediate **24**. Then, the cyclopropyl ring opens and a subsequent intramolecular nucleophilic attack affords the carbocycle **27**. In a consecutive second step, the authors performed a Zn-mediated hydroarylation process to get the polycyclic amines **22**.

**Scheme 5:**
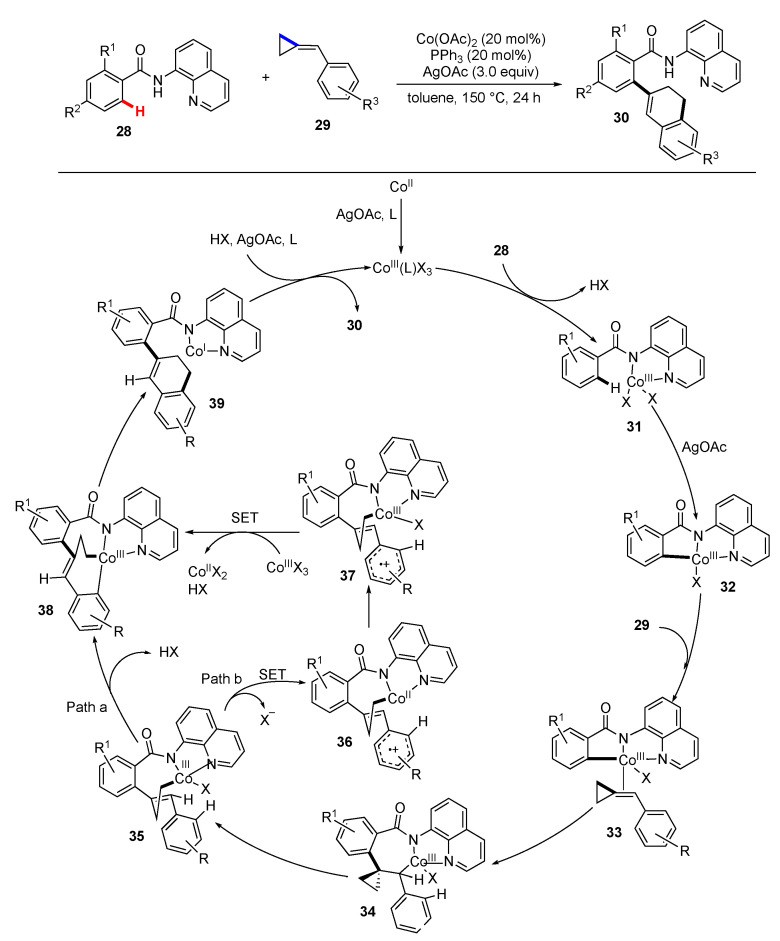
Cobalt-catalyzed reaction involving C–H/C–C cleavage.

A cobalt-catalyzed tandem C–H activation/C–C cleavage/C–H activation process was reported recently by Kwong and co-workers (Scheme 5) [52]. The reaction involved the coupling of *N*-(quinolin-8-yl)-benzamide substrates **28** and alkylidenecyclopropanes (ACPs). The chelation of the 8-amino-quinolyl fragment to Co(III) species would promote the C–H metalation to give the *C*,*N*,*N*-pincer intermediate **32**. Then, coordination and migratory insertion of the exocyclic double bond of the ACP coupling partner would render a seven-membered cobaltacycle **34**. Subsequently, the C–C bond of the cyclopropyl ring would be cleaved via β-C elimination to render a σ-alkyl Co(III) intermediate **35**. The authors proposed the evolution of **35** through two possible paths. Mechanistic studies point the most probable one to proceed through a C–H activation on the nearby phenyl ring and reductive elimination of Co(I) with C–C bond formation to afford the functionalized benzamide **30**. An alternative path involving a single-electron transfer (SET) from the adjacent phenyl ring to the metal seems less probable since no related aryl-TEMPO adducts were observed when the reaction was performed in the presence of the mentioned radical scavenger. In both paths the presence of an oxidant such as AgOAc is necessary to restore the catalytically active Co(III) species. Noteworthily, the reaction conditions tolerate the presence of halides in the aromatic rings of the starting benzamides.

**Scheme 6:**
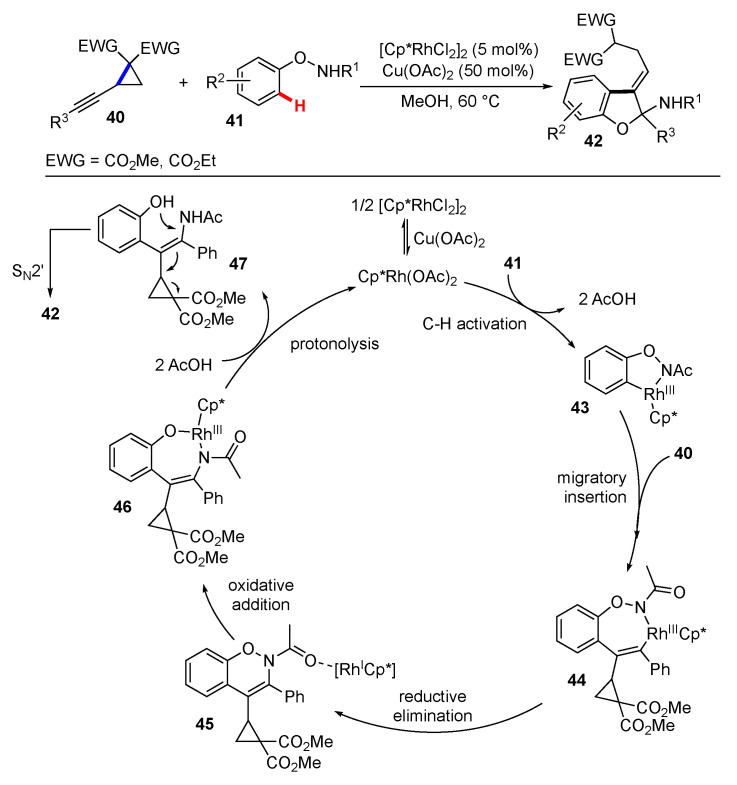
Rh-catalyzed synthesis of 2,3-dihydrobenzofuran derivatives. Cp* refers to pentamethylcyclopentadienyl ligand.

Xu’s group utilized alkynylcyclopropanes **40** to get 2,3-dihydrobenzofuranes **42** through the Rh-catalyzed coupling with *N*-aryloxyamides **41** (Scheme 6) [53]. The reaction mechanism comprised the oxyamide-directed *ortho*-C–H metalation followed by migratory insertion of the alkynyl fragment and N–C bond formation to give the intermediate **45**. The remaining O–N bond could then add oxidatively to Rh(I). Finally, the acetic acid present in the reaction mixture would activate the opening of the cyclopropyl ring and subsequent intramolecular cyclization to furnish the heterocyclic scaffolds **42**.

**Scheme 7:**
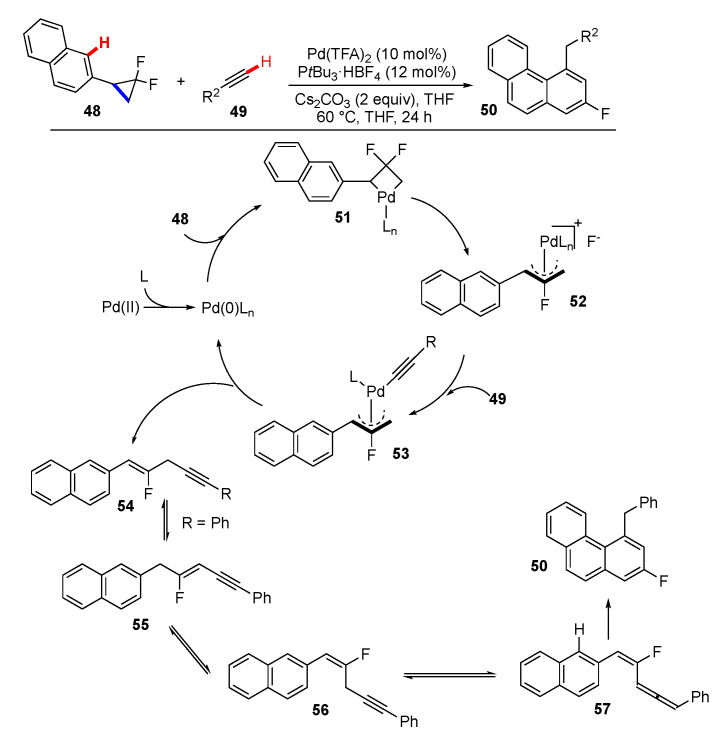
Pd-catalyzed synthesis of phenanthrenes.

Fu et al. reported the synthesis of fluorinated phenanthrenes **50** through the cleavage of a C–F, C–C and C–H bond present in *gem*-difluorinated cyclopropanes **48** (Scheme 7) [54]. The coupling reaction of cyclopropyl derivatives **48** and terminal alkynes was carried out utilizing Pd(TFA)_2_/PtBu_3_·HBF_4_ to generate the Pd(0) catalytically active species. The oxidative addition of the strained C–C bond to Pd(0) and β-fluorine elimination gave rise to the π-allyl complex **52** which was further trapped by the terminal alkyne **49**. The fluorinated eneyne **54** underwent an isomerization an intramolecular cyclization with the naphthalene core to deliver the phenanthrene structures **50**.

**Scheme 8:**
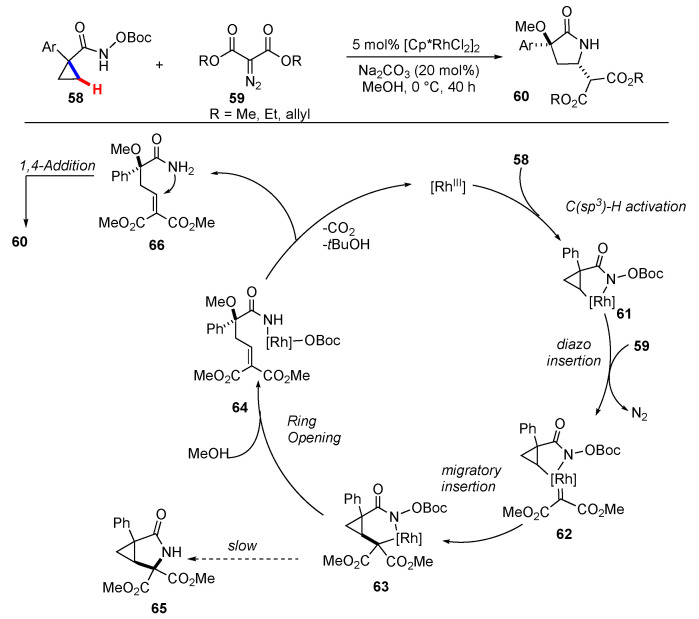
C–H/C–C activation of cyclopropane hydroxamates.

Recently, Cramer et al. developed a Rh(III)-catalyzed diastereoselective synthesis of α-alkoxylated γ-lactams **60** merging C–H and C–C bond activation (Scheme 8) [55]. The three-component reaction coupled arylcyclopropane hydroxamates **58**, diazomalonate derivatives **59** and an alcohol. The proposed mechanism starts with the coordination of the hydroxamate moiety of **58** to Rh(III), followed by Csp^3^–H activation on the cyclopropyl ring to form the five-membered rhodacycle **61**. Next, the diazomalonate would transfer the carbene moiety to the metal upon release of a molecule of N_2_, and a subsequent migratory insertion into the Rh–C bond would give an enlarged six-membered rhodacycle **63**. The cyclopropyl ring opening might be triggered by the nucleophilic attack of the alcohol on that intermediate, given the partial charges developed in such species. The C–C bond cleavage would occur with concomitant N–O bond cleavage to give the intermediate **64**. Finally, a protodemetalation step would regenerate the Rh(III) catalyst and the free amide that would undergo a 1,4-addition to the conjugated ester to render the *N*-heterocycle.

**Scheme 9:**
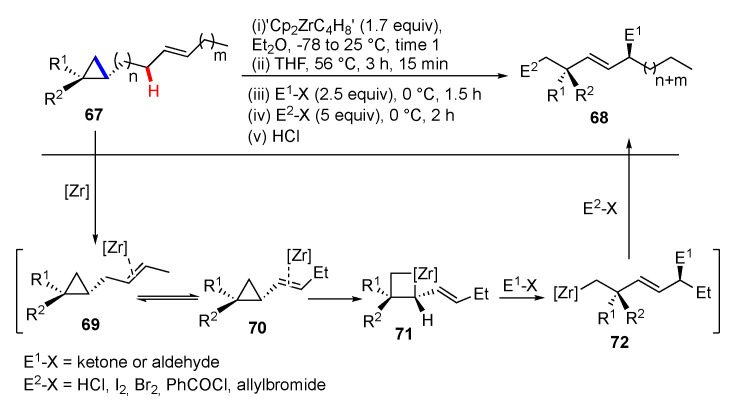
Zr-mediated functionalization of ω-ene-cyclopropane derivatives.

In 2017, Marek published a protocol to carry out the activation of allylic C–H bonds along with C–C cleavage of ω-ene-cyclopropane substrates **67** as an extension of a previous work on this topic (Scheme 9) [56,57,58]. 

The reaction was mediated by [Cp_2_Zr(η^2^-butene)] and the proposed mechanism involved the allylic C–H activation followed by olefin isomerization to reach the intermediate **70**, where cyclopropyl ring opening would lead to the four-membered zirconacycle **71**. The sequential addition of two electrophilic coupling partners such as ketones, allyl- or acyl halides and iodine among others, allowed the synthesis of the difunctionalized products **68**.

**Scheme 10:**
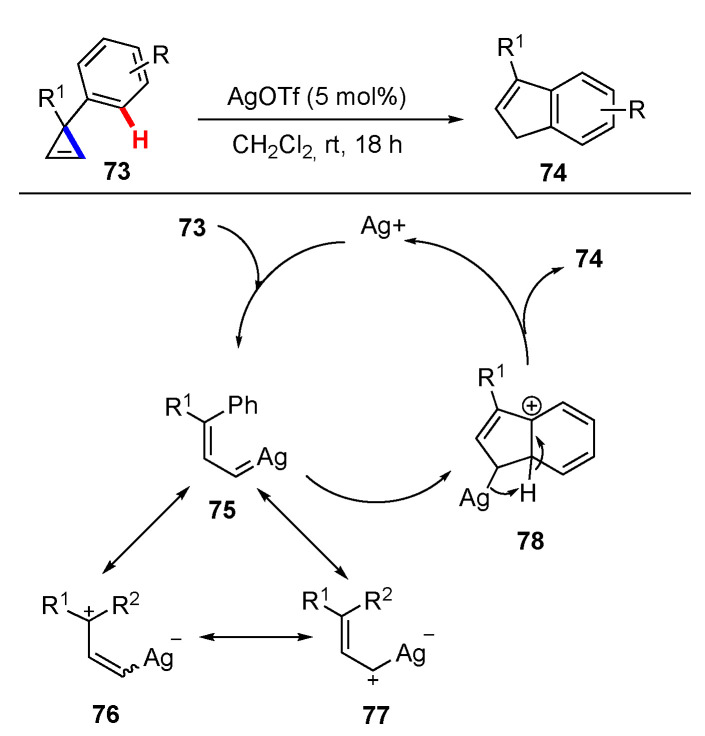
Silver-catalyzed synthesis of 1*H*-indene derivatives.

Endo et al. developed a silver-catalyzed intramolecular cyclization of 3,3-diaryl or 3,3-aryl,alkyl-cyclopropenes **73** (Scheme 10) [59]. The authors proved that silver salts such as AgOTf, AgOAc or AgF, could promote the opening of the cyclopropane ring to form a vinyl carbene intermediate **75** which could evolve through a Friedel-Crafts-type reaction to afford the desired indene core **74**.

**Scheme 11:**
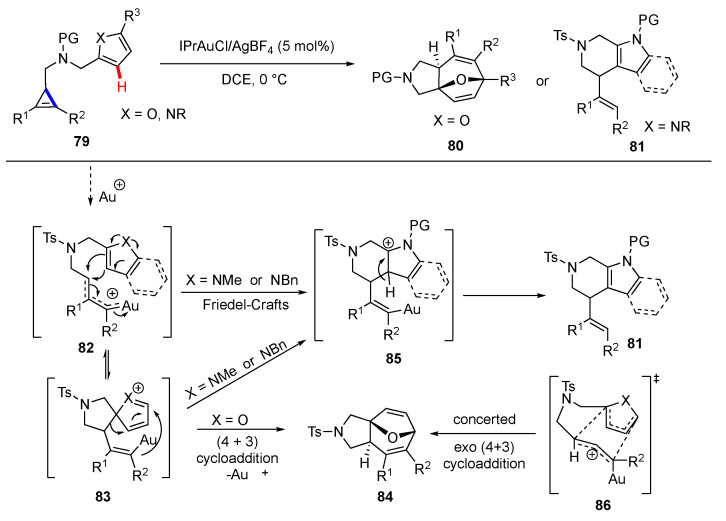
Au-catalyzed synthesis of heterocycles.

A related strategy was employed by Hashmi et al. to generate interesting heterocyclic scaffolds. In this case, cyclopropene derivatives **79** bearing a tethered heteroaromatic ring on the Csp^3^ center of the strained ring were used (Scheme 11) [60]. The outcome of the cyclization reaction was heavily dependent on the type of heterocycle present in the substrate. The proposed mechanism involved the opening of the cyclopropene fragment by Au(I) carbene catalyst to generate a vinyl gold carbene intermediate **82**. When **79** contained an indole or pyrrole substituent, a Friedel-Crafts cyclization took place to render the tetrahydro-β-carbolines **81**. 

**Scheme 12:**
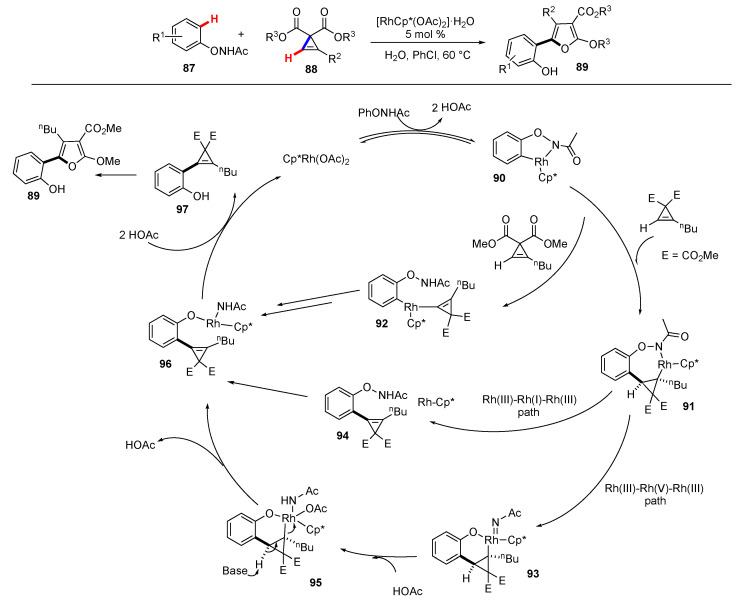
Rh-catalyzed coupling of *N*-phenoxyacetamides with cyclopropenyl esters. Cp* refers to pentamethylcyclopentadienyl ligand.

Glorius reported the synthesis of arylated furans **89** by a Rh-catalyzed coupling of *N*-phenoxyacetamides **87** with cyclopropenyl esters **88** (Scheme 12) [61]. Several mechanistic proposals were assessed experimentally. The authors concluded that the most probable path involved the *ortho*-metalation of the *N*-phenoxyacetamide substrate with formation of the five-membered rhodacycle **90**. The strained cyclopropenyl derivative would suffer the insertion into the Rh–C bond to give the seven-membered rhodacycle **91**. The Rh(III) center would then undergo an oxidative addition of the N–O bond present in the starting phenoxamide, to render a Rh(V) species **93**. Next a base-assisted *anti* β-H elimination would afford the arylated cyclopropene **97**. Under the reaction conditions, the cyclopropene unit would undergo a cycloisomerization process involving the cleavage of the C–C bond of the strained carbocycle.

**Scheme 13:**
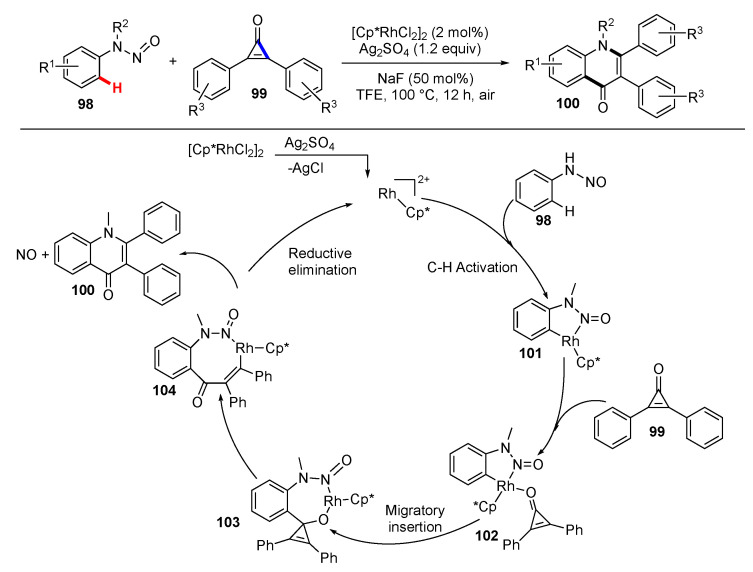
Rh-catalyzed synthesis of quinolones. Cp* refers to pentamethylcyclopentadienyl ligand.

The Chen group developed the Rh-catalyzed coupling of *N*-nitrosoanilines **98** and diphenylcyclopropenones **99** to get 4-quinolone scaffolds **100** (Scheme 13) [62]. In this case, the *N*-nitroso moiety exerted the role of directing group for the *ortho*-C–H metalation of the substrates **98**. Coordination of the carbonyl group present in **99**, and regioselective migratory insertion of the C–Rh bond led to the intermediate **103**. Further β-C elimination opened the strained ring, and later evolution via reductive elimination with extrusion of NO afforded the desired quinolones **100**.

**Scheme 14:**
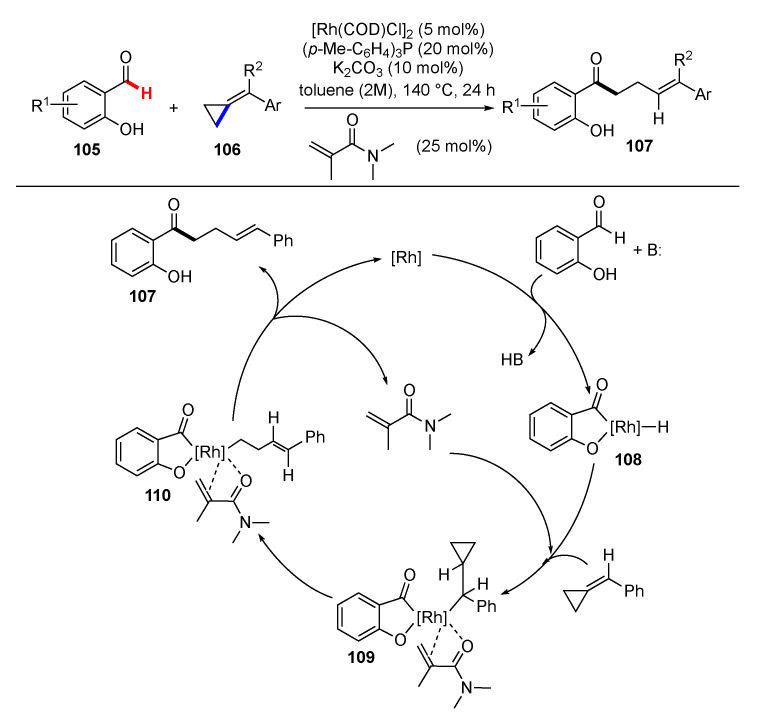
Rh-catalyzed functionalization of aldehydes.

Recently, Li described a synthetic route to γ,δ-unsaturated ketones **107** through the Rh-catalyzed hydroacylation of alkylidenecyclopropanes **106** employing salicylaldehydes **105** (Scheme 14) [63]. The reaction would be initiated with the oxidative addition of the C–H bond from the aldehyde moiety to Rh(I). Then, a hydrorhodiation of the alkylidene fragment would give the intermediate **109**. Opening of the strained carbocycle through β-C elimination would afford the acyl-alkyl-Rh(III) species **110** from where reductive elimination can take place smoothly. The success of the coupling relied on the presence of the coordinating OH group in the starting aldehyde, able to stabilize the acyl-Rh intermediate **110** and preventing decomposition routes such as decarbonylative processes.

**Scheme 15:**
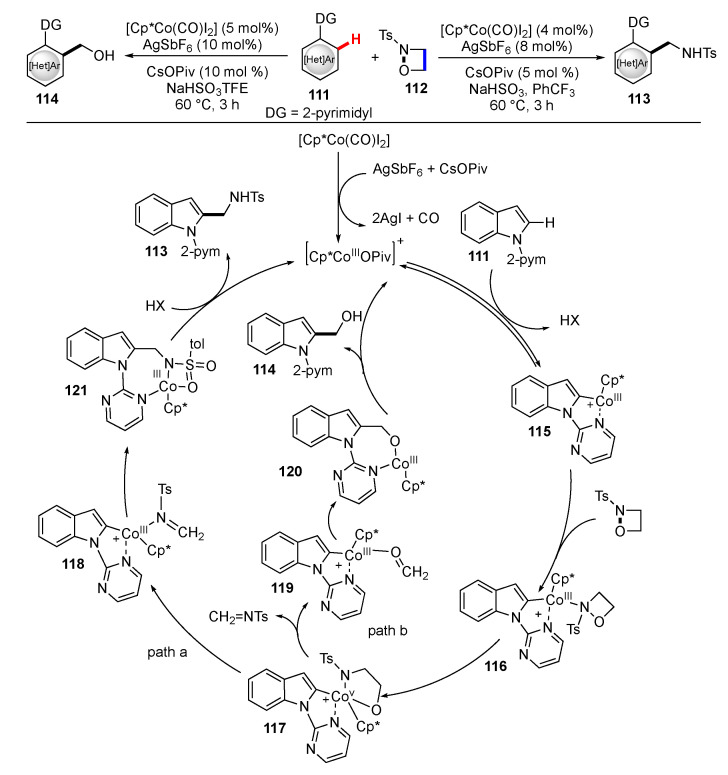
Co-catalyzed aminomethylation and hydroxymethylation of arenes. Cp* refers to pentamethylcyclopentadienyl ligand.

In 2019, Loh’s research group reported a solvent-controlled C–H aminomethylation and hydroxymethylation of arenes and heteroarenes (Scheme 15) [64]. The process exploited the cobalt-catalyzed ring-opening reaction of 1,2-oxazetidine derivatives **112**, and overall integrated the cleavage of a C–H, C–C and a N–O bond. The screening of the conditions revealed that the use of PhCF_3_ as the solvent promoted the C–H aminomethylation reaction, while trifluoroethanol favoured the hydroxymethylation instead. The proposed mechanism is triggered by the aryl or heteroaryl (mainly indole) pyrimidine-directed C–H cobaltation to give **115**. Then, the coordination of the 1,2-oxazetidine and oxidative addition of the N–O bond to the Co(III) center would afford the *N*,*O*-chelated Co(V) intermediate **117**, which could undergo two competitive β-carbon elimination process to release either a molecule of formaldehyde (path a), or a molecule or *N*-protected-formaldimine (path b) and the Co(III) species **118** or **119** respectively. In each path, the C–Co bond could insert into the O=C or N=C bond of the coordinated formaldimine or formaldehyde to give seven-membered cobaltacycles **121** or **120** respectively. Final protodemetalation would afford the functionalized products and regenerate the Co(III) active species. The authors proposed that the solvent might influence the evolution of the intermediate **117**, with protic solvents establishing hydrogen-bonding interactions with the imine moiety and favouring the formation of the intermediate **120**.

**Scheme 16:**
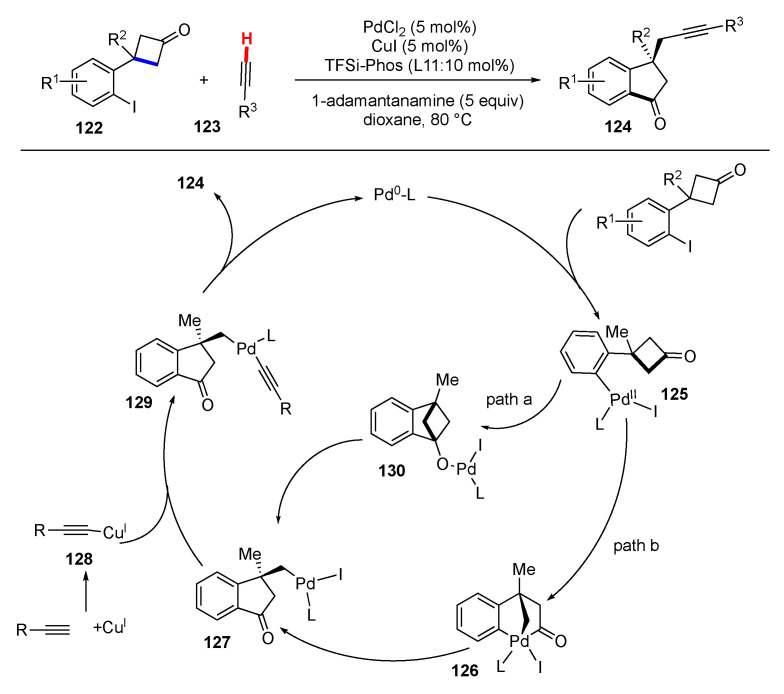
Pd-catalyzed synthesis of cyclopentanones.

Xu and co-workers tackled an enantioselective Sonogashira-type coupling of terminal alkynes with cyclobutatone substrates **122** (Scheme 16) [65]. The palladium/copper-catalyzed process required the use of a phosphoramidate ligand and 1-adamantamine base. The authors proposed two possible paths for this reaction. In both of them, the oxidative addition of the arylhalide to Pd(0) occurs in first place. In the path a, addition to the carbonyl group and regioselective C–C cleavage would render the σ-alkyl Pd(II) intermediate **127**, which could then react with the alkynylcopper species formed in situ. The path b would reach to the intermediate **126** upon oxidative addition of the C–CO bond present in the strained cyclobutanone ring. The Pd(IV) species would then evolve through a reductive elimination to give the intermediate **127**.

**Scheme 17:**
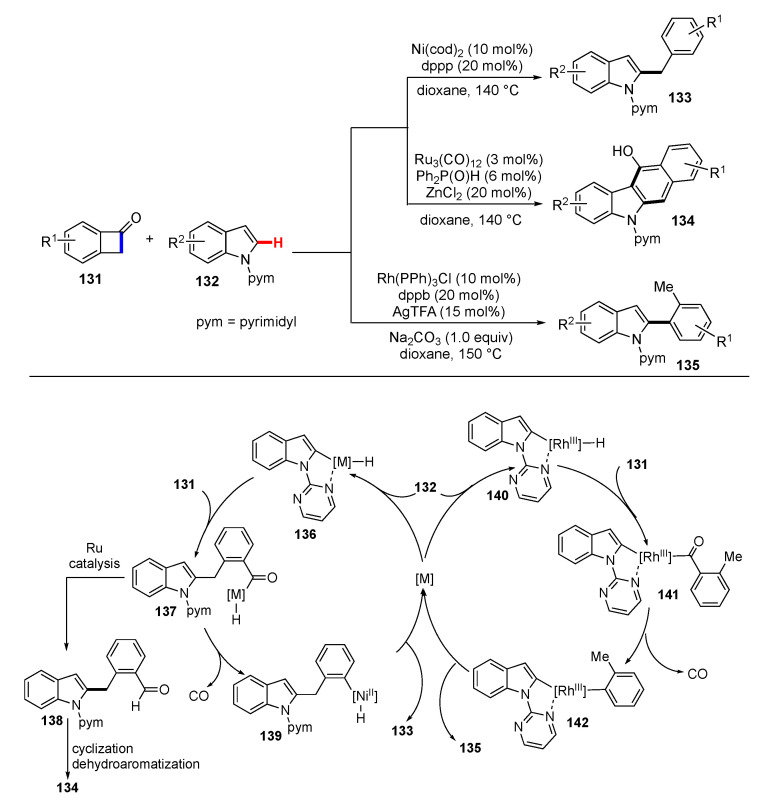
Divergent coupling of cyclobutanone and indole derivatives.

Very recently, Wei et al. exploited the reactivity of cyclobutenones **131** in a divergent cascade process that involved the C–H functionalization of indoles [66]. The result of the coupling reaction was variable according to the metal catalyst of choice (Scheme 17). The use of [Ni(cod)_2_], [Ru_3_(CO)_12_] or [Rh(PPh_3_)_3_Cl] as catalyst, led respectively to the formation of 2-benzylindoles **133**, benzo[*b*]carbazoles **134** and 2-arylindoles **135**. Mechanistically, regardless the catalyst employed, the three different reactions started with the C–H metalation through the oxidative addition of the *N*-pyrimidyl indole to the low-valence metal center. In the case of the Rh-catalyzed process, the C(O)-Csp^3^ bond of the cyclobutanone is cleaved and the resulting alkyl and hydride ligands on the coordination sphere of Rh are coupled to give the intermediate **141.** A further decarbonylation step and C–C coupling occurs, furnishing the arylated compound **135**. However, in the cases of the Ni- and Ru-catalyzed reactions, upon the C(O)-Csp^3^ bond scission, a heteroaryl-Csp^3^ coupling takes place to deliver the intermediate **137**. Afterwards the Ni-catalysis produces a decarbonylation and reductive elimination to render **133**. The Ru-catalysis affords the aldehyde **138** which evolves through an intramolecular cyclization to give **134**.

**Scheme 18:**
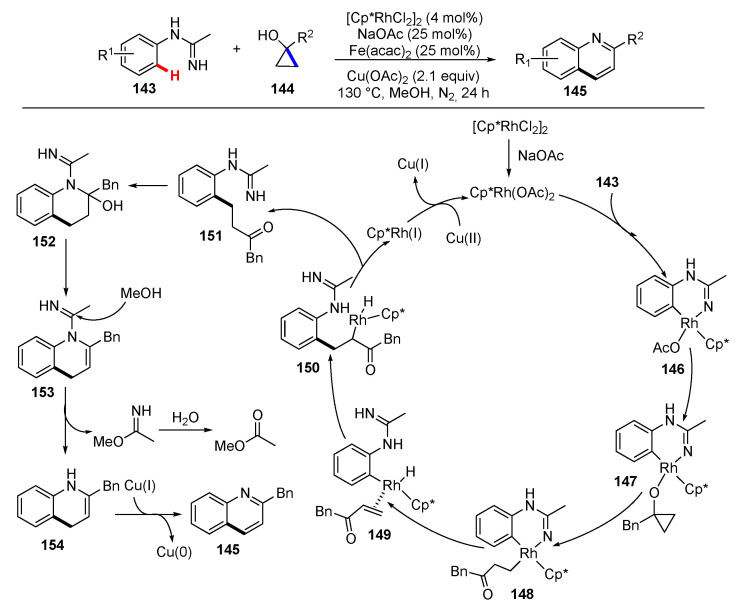
Pd-catalyzed synthesis of quinolines. Cp* refers to pentamethylcyclopentadienyl ligand.

Tertiary alcohols bearing strained rings such as cyclopropanol and cyclobutanol have been widely used in strategies involving transition-metal catalyzed C–C cleavage [38,40,41]. In this regard, Li et al. tackled the synthesis of 2-substituted quinolines **145** through the Rh(III)-catalyzed C–H activation of imidamide substrates **143** and cross-coupling with cyclopropanols (Scheme 18) [67]. 

The reaction was carried out with 4 mol% of [Cp*RhCl_2_]_2_ in the presence of NaOAc, [Fe(acac)_3_] (25 mol%) and 2.1 equivalents of Cu(OAc)_2_ as oxidant. The plausible catalytic cycle would involve the orthometalation of the amidamide substrate by Rh(III). The coordination of the cyclopropanol, followed by the ring-opening would render the alkyl intermediate **148**. Next, β-hydride elimination and insertion of the coordinated olefin gives the alkyl-Rh intermediate **150**, which upon reductive elimination affords the *ortho*-alkylated amidamide **151**. An intramolecular cyclization and oxidation process gives rise to the desired 2-substituted quinolones **145**. The scope of the reaction included benzyl-, phenoxy- and alkylcyclopropanols.

In 2019, Bi and co-workers performed DFT calculations [68] to deepen in the mechanistic aspects of the related and previously reported Rh-catalyzed ortho alkylation of arenes using cyclopropanol derivatives by means of C–H and C–C cleavage [69,70]. They concluded that the activation of the C–H bond prior to the cyclopropanol ring opening was not favourable thermodynamically. Furthermore, an outer-sphere concerted metalation-deprotonation (CMD) process seems to fit better with the experimental observations than the inner-sphere one, in which the activation energy barrier for the C–H activation is too high for a reaction taking place at room temperature in that case.

**Scheme 19:**
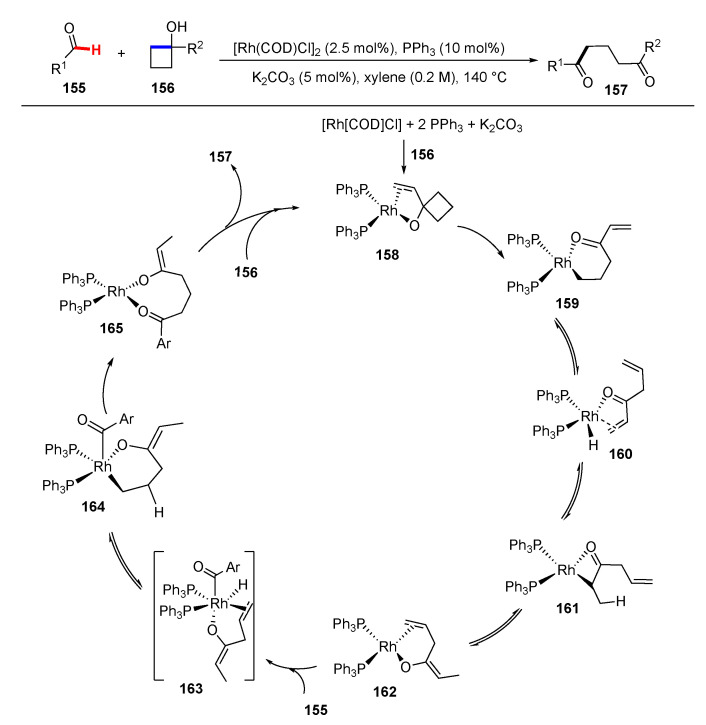
Rh-catalyzed synthesis of 1,5-diketones.

Zhang published the Rh-catalyzed synthesis of 1,5 diketones through the coupling of aldehydes and vinylcyclobutanols **156** (Scheme 19) [71]. The reaction would be initiated by the coordination of the cyclobutanol to Rh(I), followed by opening of the ring. Next, β-H elimination and re-insertion of the Rh–H moiety would give the intermediate **161**. The oxidative addition of the C–H bond of the aldehyde coupling partner would give the acyl Rh(III) species **163**. A hydrometalation step followed by reductive elimination would render the 1,5-diketone **157** and regenerate the Rh(I) active catalyst. A range of both aryl- and alkyladehydes worked out nicely as suitable substrates.

**Scheme 20:**
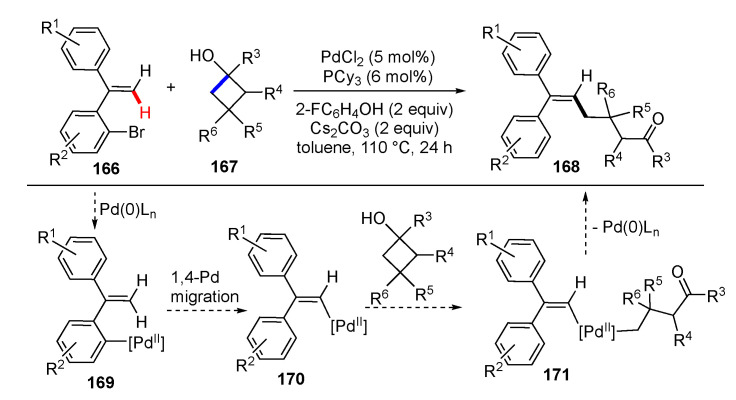
Functionalization of *gem*-diaryl alkenes through 1,4-Pd migration.

One of the strategies to functionalize unreactive remote C–H bonds relays on the generation of a carbon-metal bond on a reactive position of the molecule, which can later undergo a migration of the metal through the molecular skeleton [14,18,72,73]. Yu and co-workers made use of this approach to get the regioselective C–H alkylation of *gem*-diaryl alkenes **166** (Scheme 20) [74]. In this case the reaction was triggered by the oxidative addition of the C–Br bond present in the aryl group of the substrates **166**. 1,4-Pd migration placed the metal on the alkene moiety, which in turn reacted with the cyclobutanol coupling partner to give the alkylated olefins **168**, upon C–C cleavage of the strained carbocycle and subsequent reductive elimination. The optimization of the reaction conditions and DFT calculations showed that an auxiliary ligand such as 2-fluorophenol was beneficial to facilitate the 1,4-migration process.

### 2.2. Reactions Involving Radical Intermediates

There is a growing interest in the development of synthetic methods that combine the C–H/C–C bond cleavage of strained carbo- or heterocycles by means of radical intermediates [24,75,76]. There are several approaches in this field, depending on the way to generate the reactive radical species, for instance through the use of photochemical catalysts or the introduction of radical promoters in the reaction mixture.

**Scheme 21:**
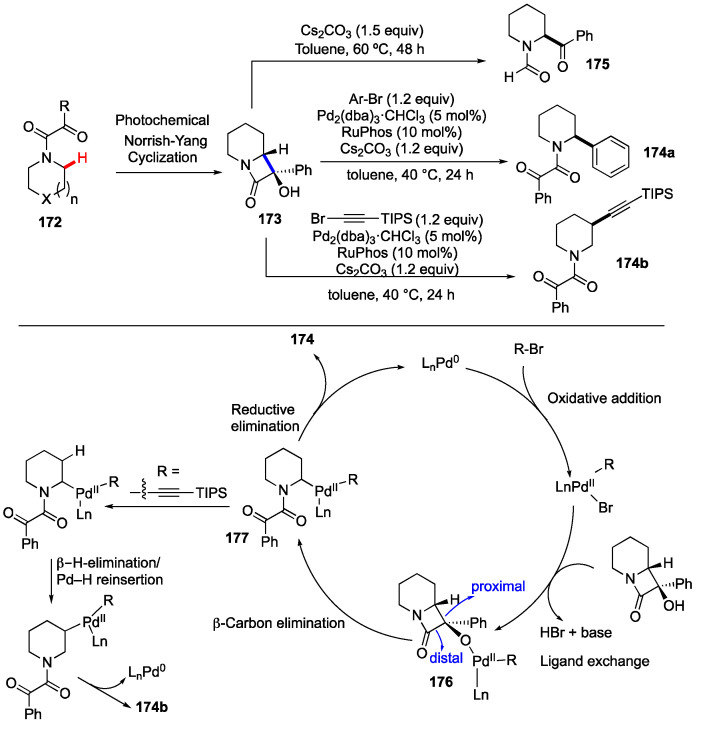
Pd-catalyzed functionalization of cyclic amines.

Inspired by Uemura’s work [77], Sarpong and co-workers developed a synthetic route for the α-C–H arylation of cyclic aliphatic amines such as piperidine or morpholine (Scheme 21) [78]. The sequential C–H and C–C functionalization starts with the conversion of the amines to the α-ketoamide derivatives **172**. These molecules can undergo a C–H functionalization by means of a photochemical Norrish-Yang Type II reaction to afford the α-hidroxy-β-lactam **173**. Next, the submission of **173** to Pd-catalysis under the conditions depicted in the Scheme 21, led to the arylated products **174a** via the following mechanism: first, the oxidative addition of ArBr to Pd(0) would give an Ar–Pd intermediate which could coordinate to the alcohol moiety present in **173**. Subsequent β-carbon elimination would lead to the α-palladated amine **177**. Finally reductive elimination of Pd(0) would afford the functionalized amine. Remarkably, the use of RuPhos ligand promoted the regioselective lactam ring opening to give the desired organopalladated intermediate **177**. In terms of substrate scope, arylbromides could be replaced by arylchlorides although the reaction required the use of XPhos ligand and heating to 100 °C to give moderate yields of the coupling product. Alkenyl- and alkynylbromides were also suitable coupling partners. Interestingly, the reaction with the (bromoethynyl)triisopropylsilane rendered the alkynylated product in β-position **174b**, instead of the expected α-functionalized one. This behaviour can be explained through a β-H elimination/re-insertion pathway taking place on the α-palladated heterocycle prior to the reductive elimination step. In addition, the authors observed that when heating the α-hidroxy-β-lactam **173** in the presence of Cs_2_CO_3_ without the Pd catalyst, a Cs-promoted distal C–C cleavage could take place to render the α-acylated derivatives **175**.

**Scheme 22:**
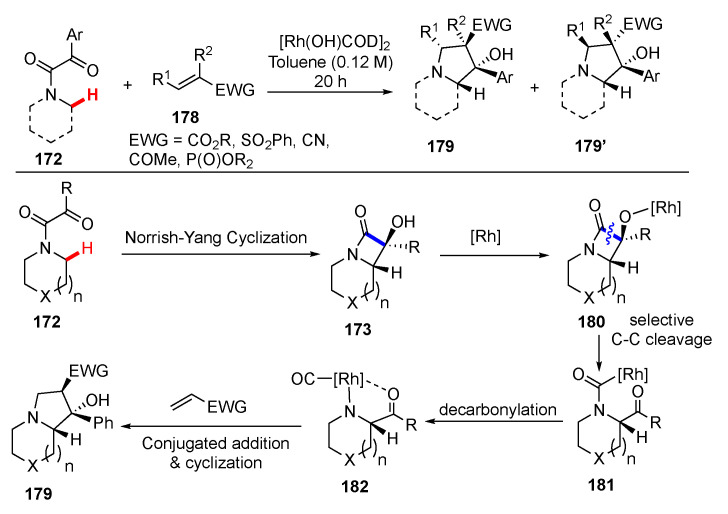
Rh-catalyzed functionalization of cyclic amines.

A related strategy was applied to the synthesis of indolizidine derivatives **179** (Scheme 22). In contrast to their previous work where a Pd catalyst was used (Scheme 21), they found that 5 mol% of [Rh(OH)(COD)]_2_ in the presence of 15 mol% of Xantphos ligand and K_2_CO_3_ could selectively promote the cleavage of the distal C–C bond through the β-carbon elimination process in **180** [79]. Then, a decarbonylation step would render the chelated complex **182**. The reaction with an acrylate molecule would be followed by the conjugated addition and subsequent intramolecular aldol reaction to furnish the desired indolizidine derivatives **179** as mixture of diastereoisomers. DFT calculations also supported a more favourable distal C–C cleavage over the proximal one in the cyclobutanol moiety under these conditions.

**Scheme 23:**
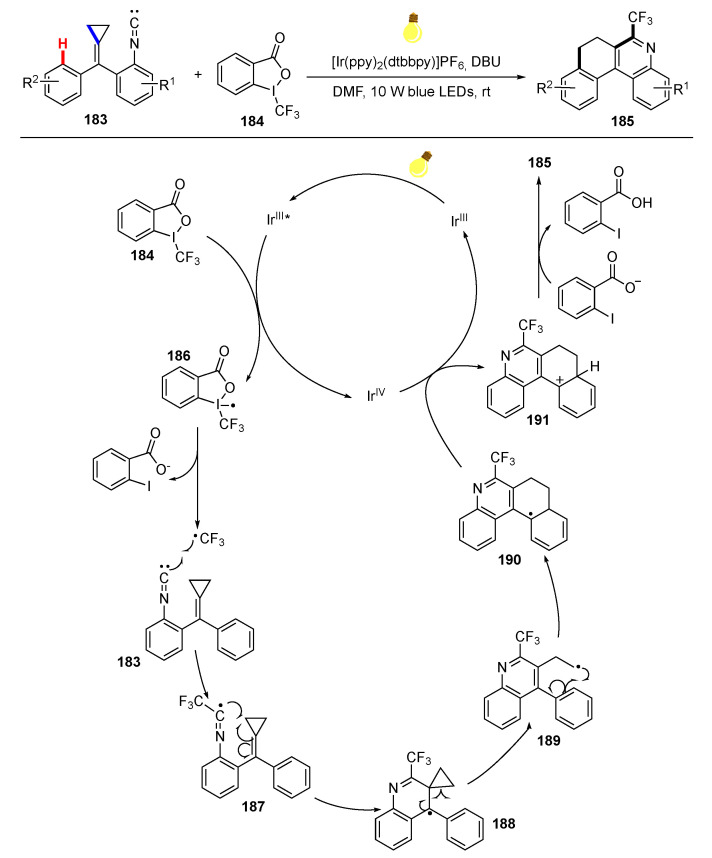
Ir-photocatalyzed synthesis of phenanthridines. The term Ir^III^* refers to the excited state of the photocatalyst.

Shi and co-workers took advantage of the photochemical generation of trifluoromethyl radicals from Togni’s reagent to establish a route to 6-(trifluoromethyl)-7,8-dihydrobenzo[k]phenanthridine derivatives **185** (Scheme 23) [80]. The authors employed conveniently designed isonitrile substituted methylenecyclopropane derivatives as starting materials and [Ir(ppy)_2_(dtbbpy)PF_6_] as photocatalyst, under blue LED irradiation. The reaction is triggered by the photochemical excitation of the Ir(III) catalyst, promoting an electron transfer to the Togni’s reagent, hence producing an Ir(IV) species, and a trifluoromethyl radical upon homolytic cleavage of the I–CF_3_ bond. The addition of the CF_3_ radical to the isonitrile moiety gives rise to the imidoyl intermediate **187**. Subsequent intramolecular cyclization and opening of the cyclopropyl unit driven by strain-release afford the intermediate **189**. The intramolecular attack of the primary radical to the aryl ring and SET of the resulting intermediate to Ir(IV), restore the Ir(III) active species and the cyclic intermediate **191**, which undergoes a deprotonation leading to the formation of the phenanthridine core. Replacing the cyclopropyl by a cyclobutyl ring in the starting material afforded the corresponding products bearing a seven-membered carbocycle.

**Scheme 24:**
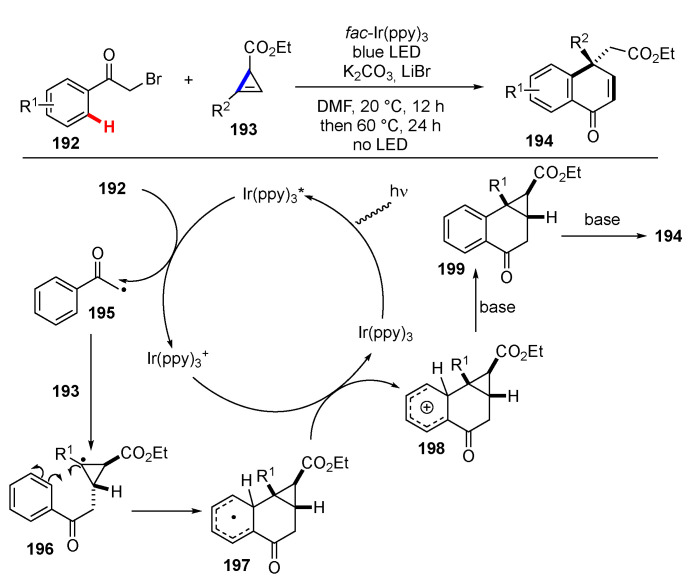
Ir-photocatalyzed synthesis of naphthalenones. The term Ir(ppy)_3_* refers to the excited state of the photocatalyst.

Landais et al. studied the synthesis of naphthalenone derivatives **194** through the addition of α-bromoacetophenones onto cyclopropenes in the presence of the photocatalyst *fac*-Ir(ppy)_3_ and visible light (Scheme 24) [81]. In this case, the excited photocatalyst can promote the electron transfer to the α-bromoacetophenones **192**, which further decomposes generating the radical **195**. The addition of such intermediate to the strained olefin renders a new radical intermediate **196** that can easily attack to the aromatic ring. The intermediate **197** is then oxidized by the Ir(IV) species to give the cationic species **198**, which recovers the aromaticity upon the loss of a proton. Finally, the basic conditions promote the opening of the cyclopropyl ring upon deprotonation in α-position to the ketone. Interestingly, the overall addition of the phenacyl moiety to the olefin occurs in a *syn* fashion, preserving the chiral stereocenter present in the cyclopropene starting material.

**Scheme 25:**
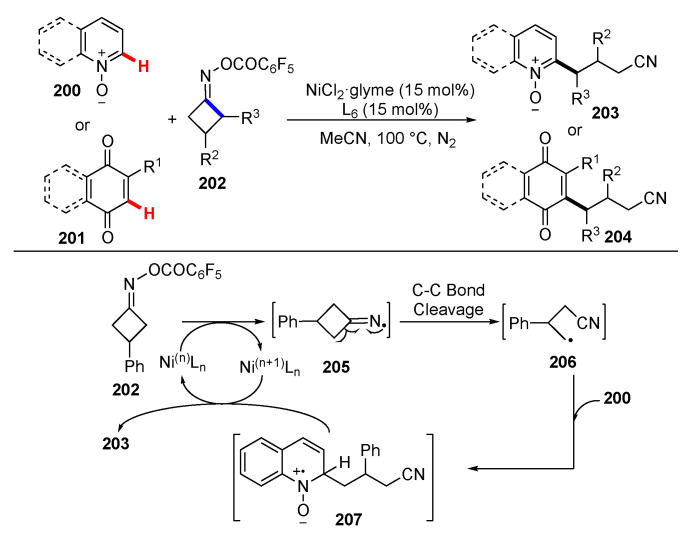
Ni-catalyzed cyanoalkylation of quinoline *N*-oxides and 1,4-quinolones.

Nitrogen-centered radical precursors have proven to be versatile and useful species in organic synthesis [82,83,84]. More specifically iminyl radical precursors have been utilized to generate cyanoalkylradical intermediates that can be further cross-coupled [75,85]. Besides, the alkylation of aromatic heterocycles through the addition of radicals, generally known as Minisci-type alkylation, is an interesting approach for C–H functionalization [86]. Hence, the use of cyanoalkylradicals generated through C–C cleavage has been recently incorporated to the Minisci-type synthetic strategy. For instance, Guo’s group reported the direct C–H cyanoalkylation of quinoline *N*-oxide **200** and 1,4-quinone **201** derivatives employing the cyclobutanone pentafluorobenzoyloxime **202** as the alkylating agent (Scheme 25) [87]. The reaction was catalyzed by NiCl_2_·diglyme in the presence of a substituted 1,10-phenanthroline ligand. The proposed mechanism involves single electron transfer from the Ni-catalyst to the oxime **202**, provoking the homolytic N–O cleavage, and subsequent C–C cleavage of the iminyl radical to generate the cyanoalkylradical species **206**. The addition of the radical to the quinoline *N*-oxide rendered the new intermediate **207**, which underwent a single-electron oxidation and deprotonation to furnish the alkylated heterocycle **203**. 

**Scheme 26:**
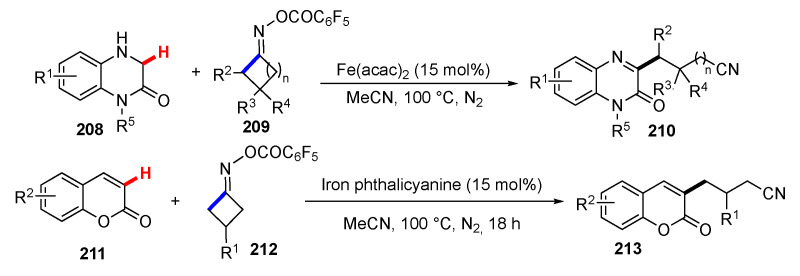
Fe-catalyzed cyanoalkylation of heterocycles.

The use of iron catalysts allowed the cyanoalkylation of quinoxalines, coumarines and 2*H*-indazoles via similar radical mechanism (Scheme 26) [88,89].

**Scheme 27:**
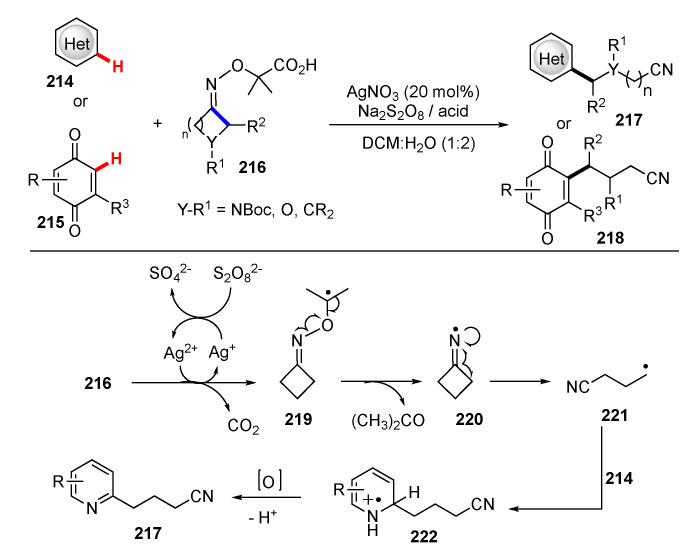
Radical cyanoalkylation of quinoline *N*-oxides and 1,4-quinolones.

The Xu group explored the cyanoalkylation of heterocycles and quinones based on Minisci-type alkylation reactions relying on iminyl-radical species generated through a decarboxylative process of substrates **216** (Scheme 27) [90]. In this case, the process would be triggered by the oxidation of Ag(I) to Ag(II) in the presence of persulfate. In turn, the Ag(II) species would promote an oxidative decarboxylation process on the α-imino-oxy acids, further releasing a molecule of acetone and the corresponding iminyl radical **220**, which would evolve as described above. Noteworthy, the reaction also proceeded nicely with less strained imino-oxy acid arising from five-membered cyclic ketones. This strategy is a complementary approach to previous ones based on reductive SET procedures to generate the desired iminyl radical.

**Scheme 28:**
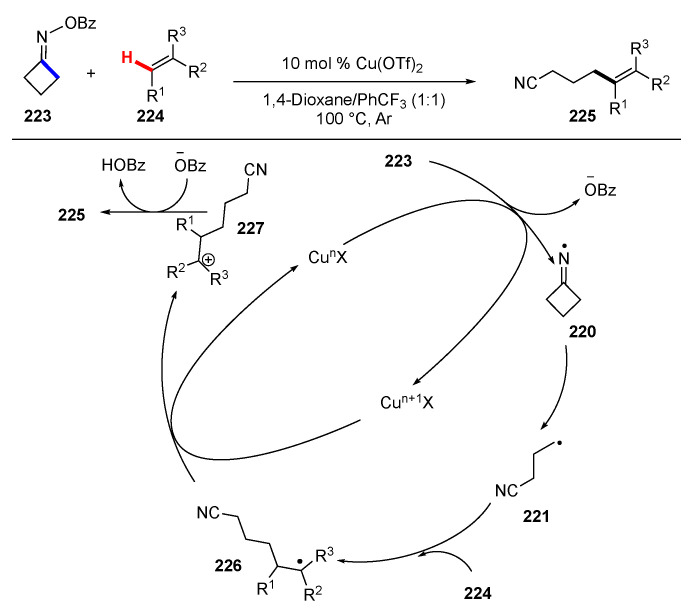
Radical cyanoalkylation of alkenes.

Shi and co-workers explored a Heck-like coupling of alkenes with the cyclobutanone oxime derivatives **223** (Scheme 28) [91]. The reaction proceeds in the presence of a copper salt, e.g., Cu(OTf)_2_, CuI or Cu powder in a mixture of 1,4-dioxane/PhCF_3_ at 100 °C under argon. The mechanistic proposal implies the generation of the iminyl radical upon a single electron transfer from copper species. The homolytic C–C cleavage renders the cyanoalkyl radical **221**, which adds to the olefin. The new radical **226** is then oxidized by copper to give a carbocation that can be easily deprotonated leading to the functionalized olefin **225**. The presence of radical scavengers totally supressed the progress of the reaction, giving further support to the radical pathway proposal.

**Scheme 29:**
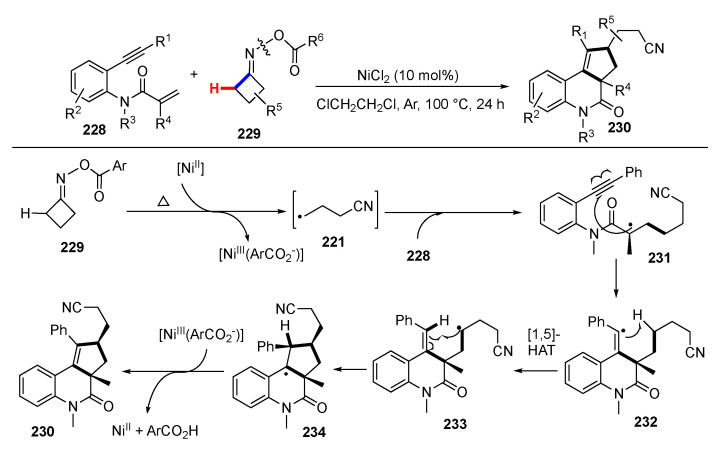
Ni-catalyzed radical cyanoalkylation/cyclization cascade.

A more complex cascade reaction was reported by Li’s group (Scheme 29) [92]. They described a NiCl_2_-catalyzed [2 + 2 + 1] carboanulation of 1,7-eneynes **228** and cyclobutanone-oximes **229**, leading to cyano-functionalized 4*H*-cyclopenta[*c*]quinolin-4-ones **230**. The procedure takes advantage of the cyanoalkyl radical generation upon single electron reduction of the cyclobutanone-oxime. The addition of the cyanoalkyl radical to the olefin enables an intramolecular cascade cyclization and 1,5-*H* abstraction leading to the species **234** that are finally oxidized by Ni(III) to release the fused carbocycle **230**.

**Scheme 30:**
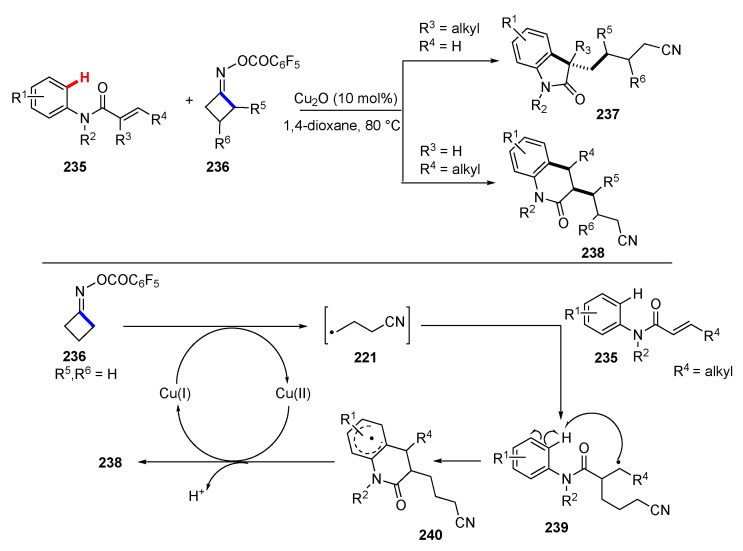
Synthesis of cyanoalkylated *N*-heterocycles.

Closely related methodology was employed to obtain 3-cyanoalkylated oxindoles **237** and dihydroquinoline-2(1*H*)-ones **238** by utilizing *N*-aryl acrylamides as starting materials (Scheme 30) [93].

The disclosure of the possibility to generate iminyl radicals under mild conditions through the SET oxidation of readily accessible α-imino-oxy acids using photoredox catalysts has allowed further applications in organic synthesis [94,95,96,97].

**Scheme 31:**
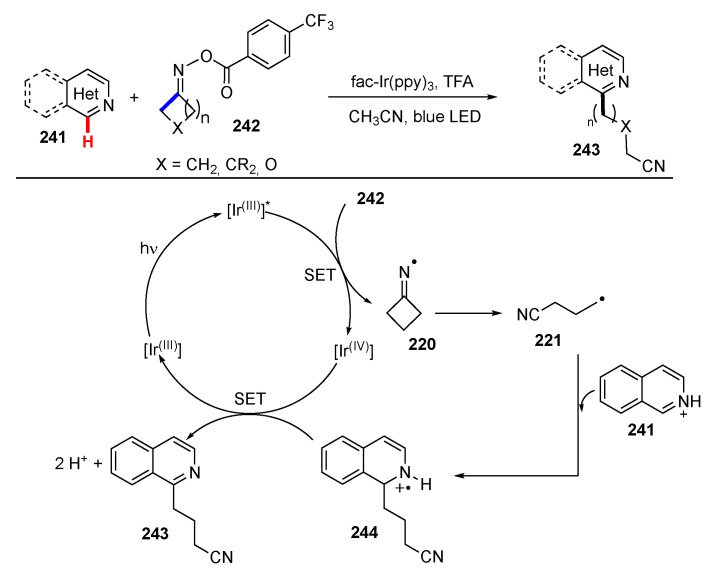
Ir-photocatalyzed cyanoalkylation of quinolines.

For instance, Xia and co-workers employed the oxime esters **242** to carry out the cyanoalkylation of quinolines and isoquinolines among other *N*-heterocyclic cores, via photocatalysis (Scheme 31) [98]. Under blue LED irradiation, the photoexcitation of *fac*-Ir(ppy)_3_ promotes the SET to the *O*-acyl oxime starting material, provoking the formation of the iminyl species **220** and subsequent homolytic C–C bond cleavage to render the cyanoalkyl radical **221**. The scope also included several substituted cycloketone oxime derivatives.

**Scheme 32:**
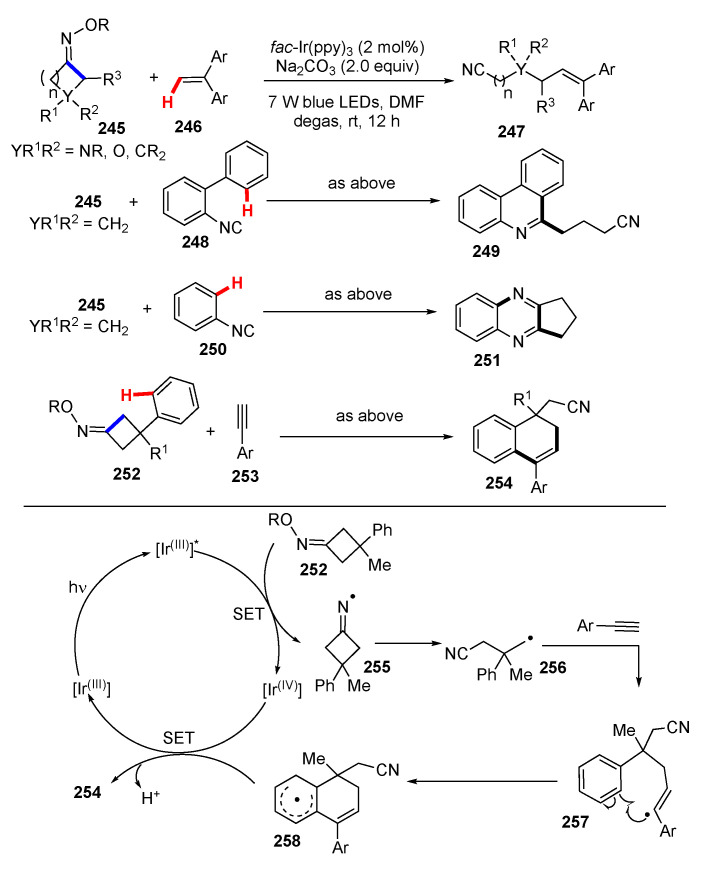
Photochemical coupling of cyanoalkyl radicals with unsaturated reagents.

Xiao’s group developed cascade reactions based on the photochemical generation of cyanoalkyl radicals and trapping with 1,1-disubstituted olefines, leading to the cyanoalkylation of the C–H bond in **246** (Scheme 32) [99]. The authors studied other unsaturated molecules bearing a pendant aryl group where the attack of the radical cyanoalkylated moiety could accomplish the C–H functionalization. For instance, the 2-phenylbenzonitrile **248** was converted to the phenanthridine derivative **249**. Related attack of cyanoalkylradicals to aryl cyanides to produce cyclopenta[*b*]quinoxalines **251** was reported by Zhang [100]. Similarly, the reactions of cyclobutanone oximes **252** containing an aryl group in β-position to the oxime moiety could furnish the 1,2-dihydronaphthalenes **254** by introducing a terminal alkyne in the reaction mixture.

**Scheme 33:**
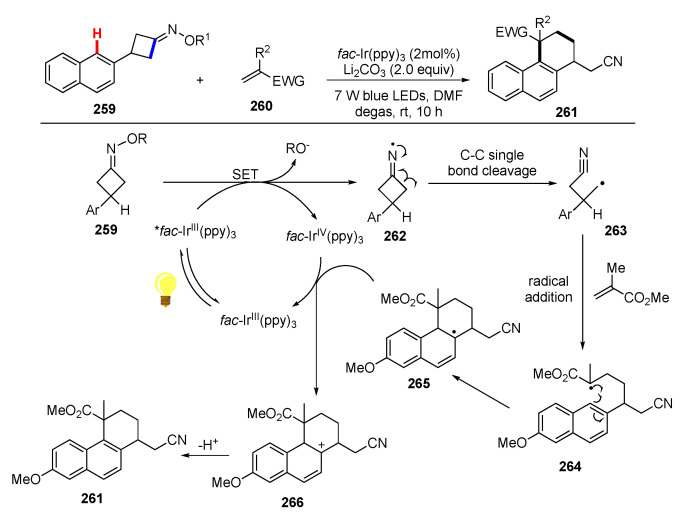
Ir-photocatalyzed synthesis of tetrahydrophenanthrenes. The term *fac-Ir^III^(ppy)_3_ refers to the excited state of the photocatalyst.

Engaged in the growing interest to explore the chemistry of nitrogen-centered radicals, Xiao and co-workers described a synthetic strategy to get 1,2,3,4-tetrahydrophenanthrenes **261** (Scheme 33) [101]. 

The overall mechanism is similar to the metal-photocatalyzed cyanoalkylation reactions described above. An excess of α-methyl methacrylate is able to capture the cyanoalkyl radical **263**, avoiding the undesired hydrogen atom abstraction that would render the reduced product. The regioselective addition of the secondary radical to the naphthyl ring would afford the cycloalkyl radical intermediate **265**. DFT calculations support a more favorable attack to the C–H in alpha position in agreement with the experimental observation. Final SET from **265** to the Ir(IV) gives back Ir(III) and the tetrahydrophenanthrene derivative upon deprotonation. 

**Scheme 34:**
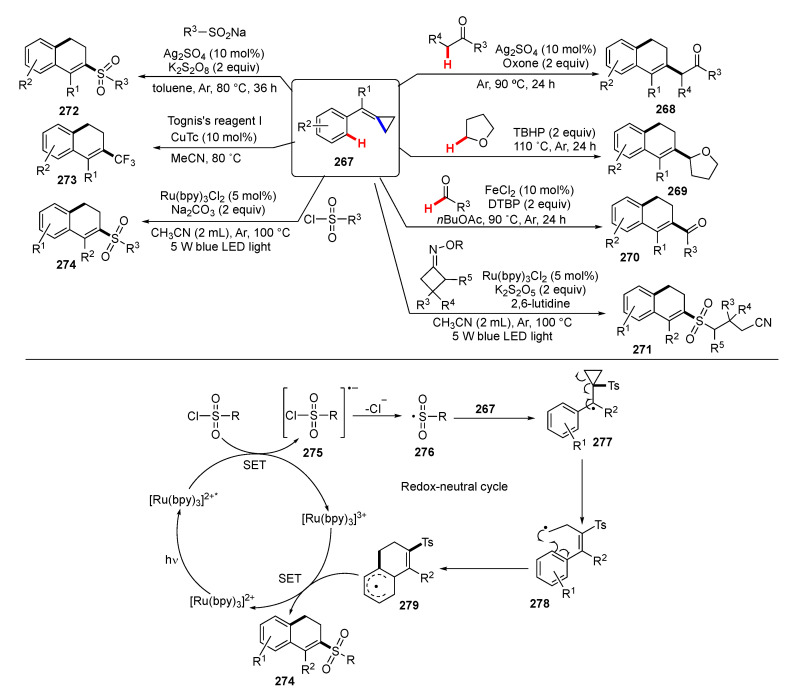
Derivatization routes of methylenecyclopropanes. The term [Ru(bpy)_3_]^2+^* refers to the excited state of the photocatalyst.

Methylenecyclopropanes (MCPs) are amenable substrates for the trapping of radicals, generating intermediates that are prone to C–C cleavage. Tang and Shi deepened in these strategies based on the attack of radical species generated in situ either chemically or photochemically (for instance CF_3_ [102], SCF_3_ [103], alkyl [104,105], acyl [106], sulfonyl [107,108] or cyanoalkylsulfonyl radicals [109]) to the exocyclic C=C bond of the MCP, generating the opening of the cyclopropyl ring and subsequent attack of the resulting alkyl radical to the aromatic ring (Scheme 34). For instance, the Ru-photocatalyzed approach to the synthesis of 3-sulfonyl-1,2-dihydronaphthalenes **274** employed (cyclopropylidenmethyl)benzene derivatives **267** and aryl or alkyl sulfonylchlorides as starting materials, in the presence of 5 mol% of [Ru(bpy)_3_Cl_2_] catalyst under blue LED irradiation. The excited Ru catalyst can act as a SET reducing agent, generating the sulfonyl radicals **276** which can add to the C=C bond of the cyclopropyliden derivative, giving rise to the benzyl radical **277**. Next, strain-promoted C–C homolytic cleavage would give the alkyl radical species **278**, which could subsequently attack the neighbouring aryl group. Finally, single electron oxidation by the Ru(III) species would furnish the functionalized carbocycles **274**.

### 2.3. Reactions Promoted by Lewis or Brønsted Acids or Bases 

Alternatively to the transition-metal catalysis and radical approaches described in the previous sections, the opening of strained rings can be promoted thorough the interaction of some substituents tethered to a strained ring with Lewis acids, either metallic cations or proton sources [110]. In some occasions these ring-opening processes are accompanied by additional C–H functionalization steps. 

**Scheme 35:**
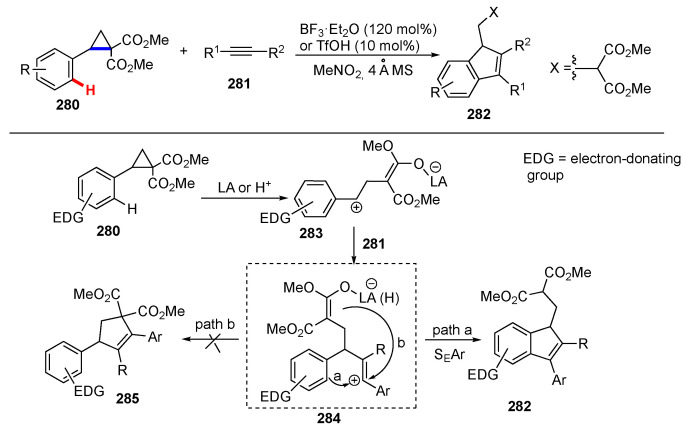
Lewis-acid promoted C–H/C–C functionalization.

Budynina developed the [3 + 2] cycloaddition reaction of donor-acceptor cyclopropanes **280** and alkynes promoted by Lewis acids such as BF_3_·Et_2_O (120 mol%) or triflic acid (10 mol%) (Scheme 35) [111]. In this particular annulation reaction, the donor-acceptor cyclopropane behaves as a 1,3-carbodipole, where the nucleophilic site is the *ortho*-C–H bond of the aromatic or heteroaromatic ring present in the substrates **280**. The opening of the cyclopropyl unit is triggered by the interaction of the Lewis acid with the ester groups. Next, attack of the carbocation to the alkyne followed by intramolecular attack to the aryl ring provokes the C–H functionalization, providing the indene derivatives **282**.

**Scheme 36:**
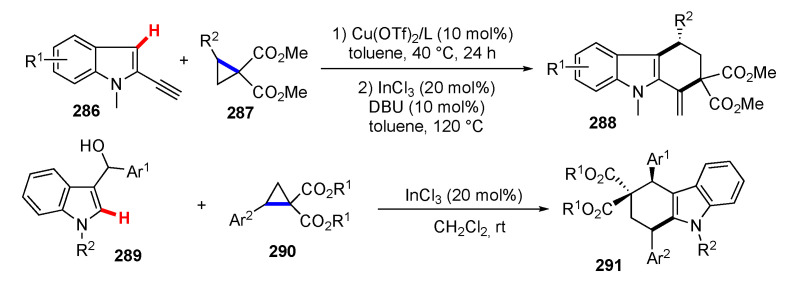
Derivatization of indoles through C–H/C–C bond cleavage.

Related methodologies that exploited the Lewis acid-promoted opening of donor-acceptor cyclopropanes **287** to functionalize the C–H bond of indole cores **286** and **289** were reported by Banerjee and Tang (Scheme 36) [112,113].

**Scheme 37:**
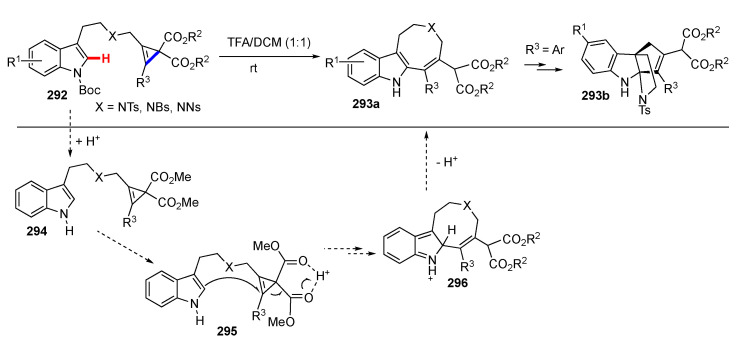
Lewis-acid catalyzed opening of *gem*-diester substituted cyclopropenes.

Shi et al. explored the reactivity of *gem*-diester substituted cyclopropenes **292** containing a pendant indole core on one of the Csp^2^ atoms of the strained carbocycle (Scheme 37) [114]. They uncovered a Lewis acid-catalyzed cycloisomerization reaction which outcome depended on the substitution of the second Csp^2^ of the cyclopropene moiety: when it included a H atom azocino[5,4-*b*]indoles **293a** were obtained, however when it contained an aryl group tetracyclic (epiminoethano)cyclopenta[*b*]indole derivatives **293b** were produced. The proposed reaction path involved the Boc deprotection of the indole, followed by activation of the cyclopropene ring through the Lewis acid (H^+^, Ag^+^ or Au^+^) coordination to the ester groups to give a cationic intermediate. Next, an intramolecular Friedel-Crafts reaction on the C2 position of the indole followed by deprotonation or demetalation would give the derivatives **293a**. Interestingly, when the substrate bears an aryl group, the analogue product keeps on reacting to render the tetracyclic structure **293b**.

**Scheme 38:**
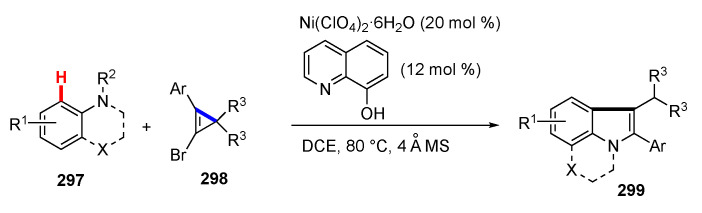
[3 + 2] Annulation reaction involving C–H/C–C cleavage.

A related Lewis acid activation of *gem*-diester cyclopropenes was reported by Tang et al. (Scheme 38) [115]. In this case, the reaction of dialkyl 2-bromo-3-phenylcyclopropene-1,1-dicarboxylate derivatives **298** with anilines, in the presence of a catalytic amount of Ni(ClO_4_)_2_·6H_2_O and 8-hydroxyquinoline ligand, led to the formation of 2,3-disubstituted indoles **299** upon a [3 + 2] annulation reaction. The replacement of anilines by tetrahydroisoquinolines as starting materials gave rise to the tricyclic indole derivatives. In addition, this methodology was implemented in a concise synthesis of Paullone, a potent kinase inhibitor.

**Scheme 39:**
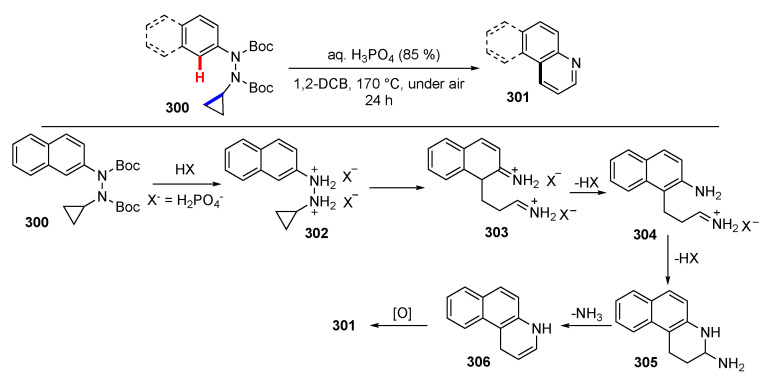
H_3_PO_4_-mediated synthesis of quinoline derivatives.

List and co-workers described recently an H_3_PO_4_-mediated synthesis of quinoline derivatives (Scheme 39) [116]. 

The reaction employed bis-Boc-protected *N*-aryl-*N*′-cyclopropylhydrazine **300** as starting materials. The reaction is initiated through the *N*-Boc deprotection to generate a protonated hydrazide salt **302**. This species can evolve via a pseudo-sigmatropic rearrangement involving the opening of the cyclopropyl ring to deliver the iminium species **303**. A re-aromatization would provide the intermediate **304**, which further evolves under the experimental conditions through an intramolecular cyclization, elimination of NH_3_ and oxidation to give the heteroaryls **301**. Depending on the conditions other *N*-heterocycles can be formed.

**Scheme 40:**
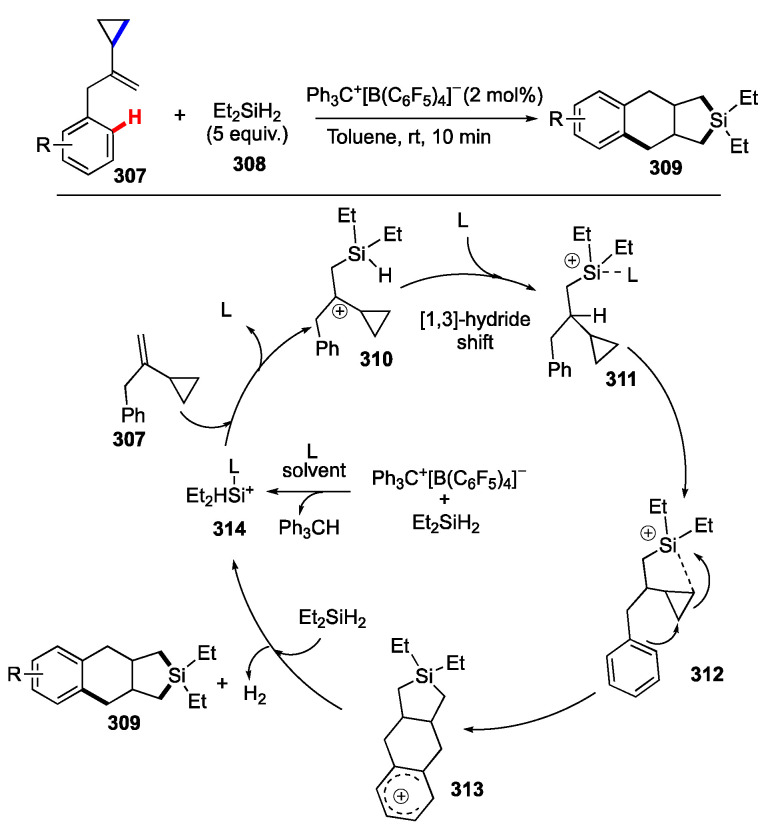
Coupling of benzyl-substituted VCP’s and hydrosilanes.

Oestreich et al. developed an interesting route to silicon-containing polycyclic structures **309** through the coupling of benzyl-substituted VCPs **307** and hydrosilanes **308** (Scheme 40) [117]. The process employed the trityl cation derivative Ph_3_C^+^[B(C_6_F_5_)_4_]^−^ as an initiator to generate a silylium-ion from the hydrosylanes **308**. The attack of this species to the VCP would render the β-silylcarbenium ion **310**, which upon a [1,3]-hydride shift would afford the intermediate **311**. The intramolecular interaction of the silylcation with the cyclopropyl ring would promote an intramolecular Friedel-Craft alkylation to render the Wheland intermediate **313**, which could be deprotonated by a molecule of Et_2_SiH_2_, rendering the desired cyclic compound **309** and regenerating the active silylium-ion species.

## 3. Synthetic Methodology Involving C–H Functionalization Along with the Cleavage of Unstrained C–C Bonds

The scission of C–C bonds in acyclic carbon skeletons is a challenging topic since these processes lack the release of the strain present in small carbocyclic structures as a driving force. Nevertheless, this fact has not precluded the development of synthetic methods where unstrained C–C bonds are cleaved [22,118,119,120]. Furthermore, in some cases these processes have been engaged with the functionalization of C–H bonds either intra- or intermolecularly. 

### 3.1. Reactions Involving Transition-Metal Catalyzed or Mediated Processes and Unstrained Substrates

As happens with the cyclopropyl and cyclobutyl alcohol derivatives commented in the previous section, the OH group of unstrained alcohols has served as a useful handle to induce the scission of the C–C bond through transition-metal catalysis [118,119]. 

**Scheme 41:**
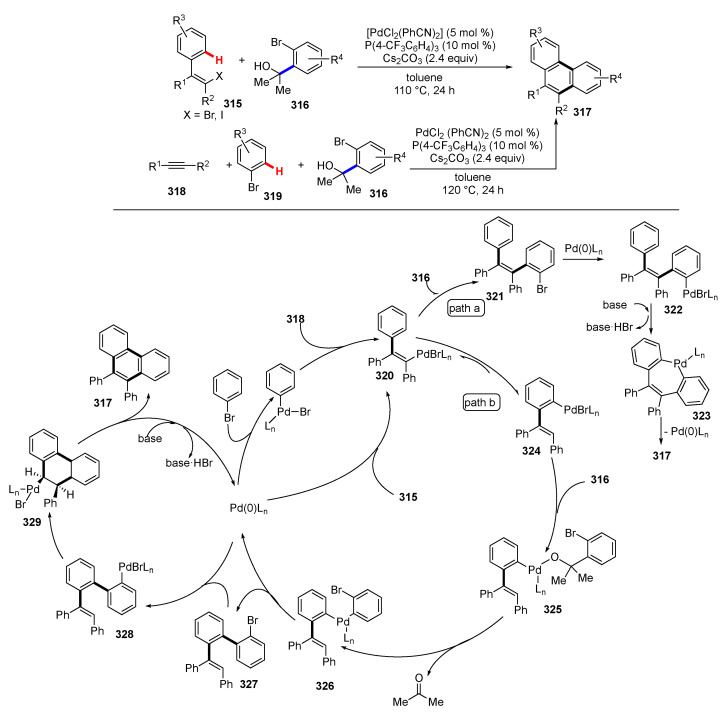
Pd-catalyzed synthesis of phenanthrenes.

For instance, Iwasaki et al. exploited the β-carbon elimination of substituted benzyl alcohols **316** in cross-coupling reactions with (Z)-β-halostyrenes **315** to get phenanthrene derivatives (Scheme 41) [121]. They employed 5 mol% of [PdCl_2_(PhCN)_2_] and 10 mol% of P(4-CF_3_-C_6_H_4_)_3_ ligand. The use of an electron poor phosphine was key to control the regioselective oxidative addition of the C–Br bond present in the halostyrenes **315** over the C–Br bond of the coupling partner **316**. Two possible reaction paths were proposed according to the results of several mechanistic experiments carried out by the authors. In both of them, the formation of the alkenyl-Pd intermediate **320** would happen in first place. **320** could directly react with the benzyl alcohol **316** to afford the intermediate **321** upon C–C cleavage and reductive elimination (path a). The remaining C–Br bond present in **321** can add oxidatively to Pd(0) and further C–H activation on the neighbouring aryl group would lead to a *C*,*C*-palladacycle **323** from where reductive elimination can release the phenanthrene core **317**. The path b involves a 1,4-Pd migration in **320** to give the aryl-Pd species **324**. Following similar steps to the path a, the reaction with **316** would render the 2-bromobiaryl **327**. Next, oxidative addition to Pd(0) and intramolecular Heck coupling with the pending alkene moiety would provide the desired polycyclic compound. The process could also be carried out as a three-component reaction by replacing (Z)-β-halostyrenes **315** by the arylbromide **319** and an alkyne.

**Scheme 42:**
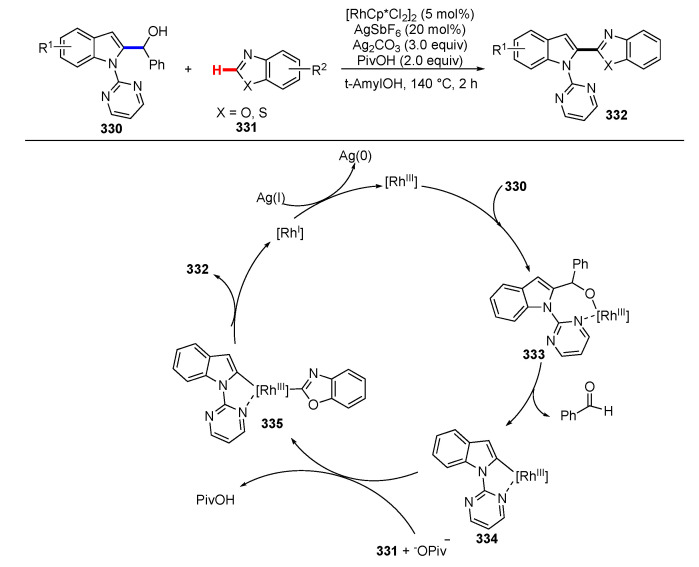
Rh-catalyzed heteroarylation of indole derivatives. Cp* refers to pentamethyl-cyclopentadienyl ligand.

The Rh-catalyzed C-2 heteroarylation of indole derivatives **330** was developed recently by Yu and co-workers (Scheme 42) [122]. The transformation relied on the favourable generation of the five-membered rhodacycle intermediate **334**. The coordination of the *N*-pyrimidyl group to Rh(III) in **333** promoted the β-carbon elimination over the possible β-hydrogen elimination process in the secondary alcohol moiety. The presence of pivalic acid in basic conditions favoured the metalation of heterocycles such as benzoxazoles, 5-substituted oxazoles, or benzothiazoles. Reductive elimination from intermediate **335** with concomitant C–C bond formation affords the heterobiaryls **332** in good yields and Rh(I) species. The presence of Ag_2_CO_3_ in the reaction mixture is necessary to re-oxidize the metal catalyst. The authors were able to isolate the rhodacycle **334** from the stoichiometric reaction of the substrate **330** and [RhCp*Cl_2_]_2_. This intermediate proved to be competent when used as catalyst in place of [RhCp*Cl_2_]_2_.

**Scheme 43:**
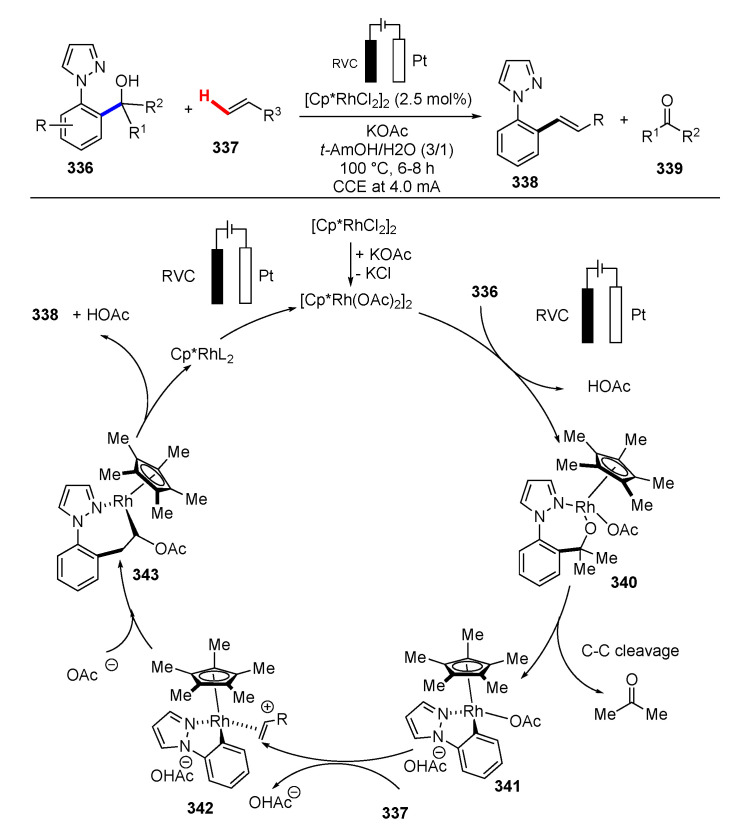
Rh-catalyzed alkenylation through C–C cleavage. Cp* refers to pentamethylcyclopentadienyl ligand.

In 2019, Ackermann tackled the use of electricity [25] in place of a stoichiometric amount of oxidant to accomplish the alkenylation of secondary and tertiary benzylic alcohols **336** (Scheme 43) [123]. 

The reaction conditions involved the use of a mixture of *t*-AmylOH/water as solvent, KOAc as additive, and 2.5 mol% of [Cp*RhCl_2_]_2_ catalyst, in an undivided electrochemical cell containing a platinum plate cathode and a reticulated vitreous carbon (RVC) anode, at 100 °C. Mechanistically, the first steps of this transformation are similar to other reactions based on β-carbon elimination of substituted benzylic alcohols commented above. The migratory insertion of the olefin into the C–Rh bond of the rhodacycle **341** and subsequent β-hydrogen elimination allows the C–H functionalization of a range of styrene coupling partners. Notably, the presence of halogens and cyano groups was tolerated under the reaction conditions. The anodic oxidation of the Rh(I) species to regenerate the active Rh(III) catalyst avoids the generation of metal-containing by products produced when oxidants such as silver or copper salts are employed.

**Scheme 44:**
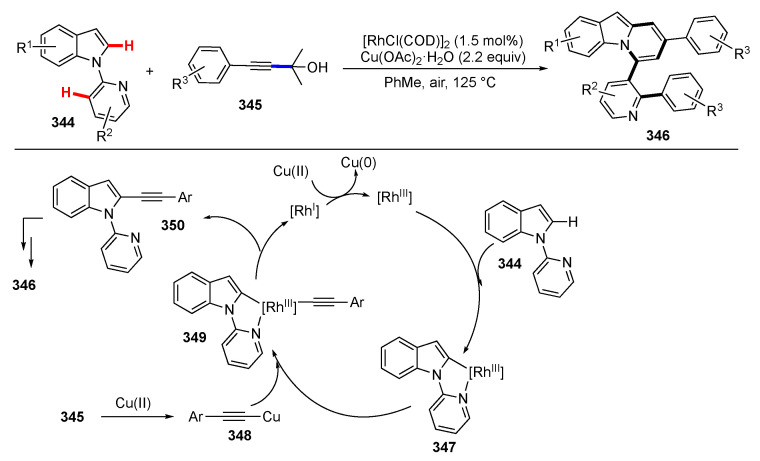
Rh-catalyzed multiple C–H and C–C bond cleavage.

*tert*-Propargyl alcohols have successfully been used as masked terminal alkynes [124]. Wen and co-workers made use of this ability in an impressive Ru/Cu-catalyzed cascade process to synthesize pyrido[2,1-*a*]-indoles **346** from 1-(pyridin-2-yl)-1*H*-indoles **344** (Scheme 44) [125]. The overall transformation involved the cleavage of two C–H, three C–C and one C–N bonds with concomitant formation of various C–C bonds. Several mechanistic experiments revealed that Rh(III) species were responsible of the C–H activation at the 2 position of the indole moiety, while Cu(OAc)_2_ performed the β-C elimination of the *tert*-propargyl alcohol **345** to render Cu-alkynyl intermediates. The transmetalation of the alkynyl group from copper to rhodium would precede the reductive elimination of Rh(I) and C–C bond formation, to give the 2-alkynylated indole **350**. The mechanistic features to reach the final pyrido[2,1-*a*]-indoles scaffolds from **350** could not be unveiled. Noteworthy, the replacement of *tert*-propargyl alcohol **345** by the corresponding terminal alkyne led to a much more extensive formation of undesired alkyne homocoupling products.

**Scheme 45:**
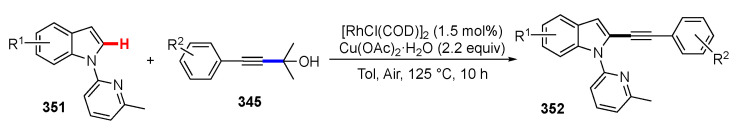
Rh-catalyzed C–H alkynylation of indole derivatives.

Further investigation revealed that the reaction could be driven to the synthesis of 2-alkynylated indoles **352** by installing a 6-methylpyridin-2-yl directing group on the nitrogen atom of **351** [126]. Similar experimental conditions for this Ru/Cu-cooperative process were applied to get a broad substrate scope of alkynylated indoles and carbazoles (Scheme 45).

**Scheme 46:**
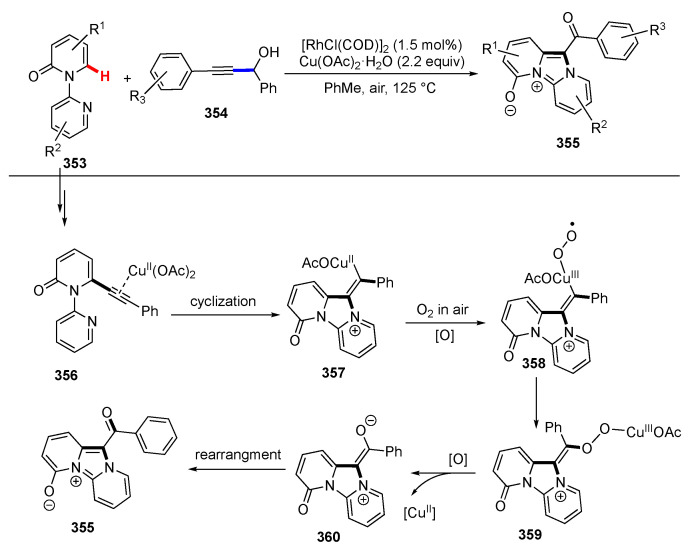
Rh-catalyzed C–H alkynylation of 2-pyridinone and further cascade process.

Li et al. described the Rh-catalyzed/Cu-mediated C–H alkynylation of 2-pyridinone derivatives **353** with propargyl alcohols under air to render 11-acylated imidazo[1,2-*a*:3,4-*a*’]dipyridin-5-ium-4-olates **355** (Scheme 46) [127]. The presence of a 2-pyridyl group in **353** promoted the coordination to Rh and subsequent C–H metalation. Alkynyl-Cu species generated upon beta-C cleavage from the propargyl alcohol are transmetallated to the Rh center, which in turn performs the C–C bond formation to give the alkynylated pyridinone **356**. Under the reaction conditions, Cu(II) could activate the alkyne to undergo the intramolecular nucleophilic attack of the nitrogen atom from the pyridyl substituent, rendering the intermediate **357**. The authors proposed that the reaction with molecular oxygen could afford the peroxycopper species **359**, which under these oxidative conditions could evolve to the acylated heterocycle **355**. The mechanistic proposal was supported by experiments carried out with H_2_^18^O, where no incorporation of ^18^O was observed in the products **355**. In addition, the substitution pattern of the propargyl alcohol coupling partner affected deeply to the yield of the process, while those secondary alcohols bearing a phenyl group worked nicely, the tertiary propargyl derivatives failed to provide the coupling product.

**Scheme 47:**
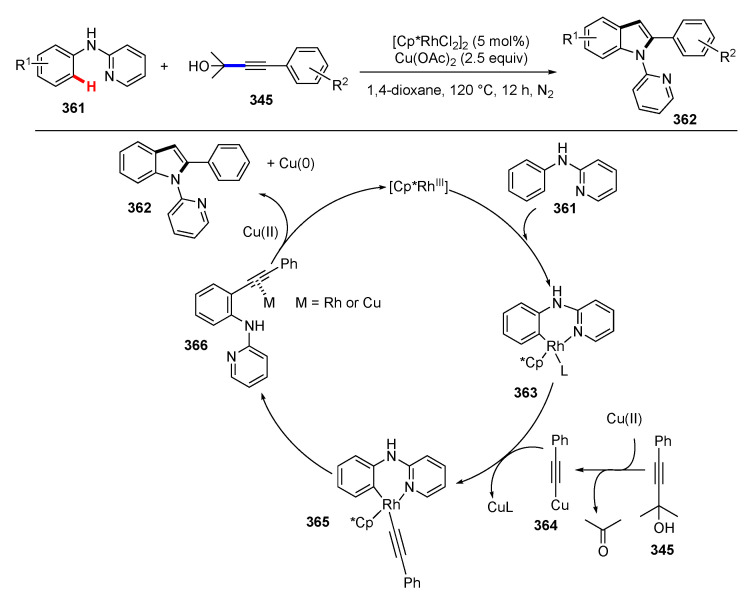
Rh-catalyzed synthesis of 2-arylindoles. Cp* refers to pentamethylcyclopentadienyl ligand.

The Zhou research group developed the synthesis of 2-arylindoles via annulative coupling of *N*-aryl-2-aminopyridines **361** and propargyl alcohols involving a selective C–H/C–C bond activation (Scheme 47) [128]. The process required the use a Rh(III) catalyst and 2.5 equivalents of Cu(OAc)_2_. The proposed mechanism involved the *ortho*-C–H metalation of the aniline promoted by the pyridine directing group to give the rhodacycle **363**. Meanwhile the C–C cleavage of the propargyl alcohol coupling partner could be accomplished by Cu(OAc)_2_, generating the alkynyl copper species **364**. Next, the transmetalation of the alkynyl fragment to the Rh center followed by reductive elimination would form the 2-alkynylated aniline **366**. Under the reaction conditions, either Rh or Cu species could activate the internal alkyne moiety in **366** through π–coordination and promote the intramolecular cyclization leading to the desired functionalized indole scaffolds. During their study, the authors were able to isolate the rhodacycle **363** intermediate. They also proved that the C–C cleavage was carried out by Cu(OAc)_2_, since an independent reaction of propargyl substrates **345** with this copper salt afforded the corresponding Glaser coupling product.

Slightly different experimental conditions allowed the replacement of the tertiary propargyl alcohol by propargylic amines as suitable coupling partners where C–C cleavage could take place [129]. A range of 2-arylated indole derivatives were obtained in moderate to good yields, following a similar reaction pathway.

**Scheme 48:**
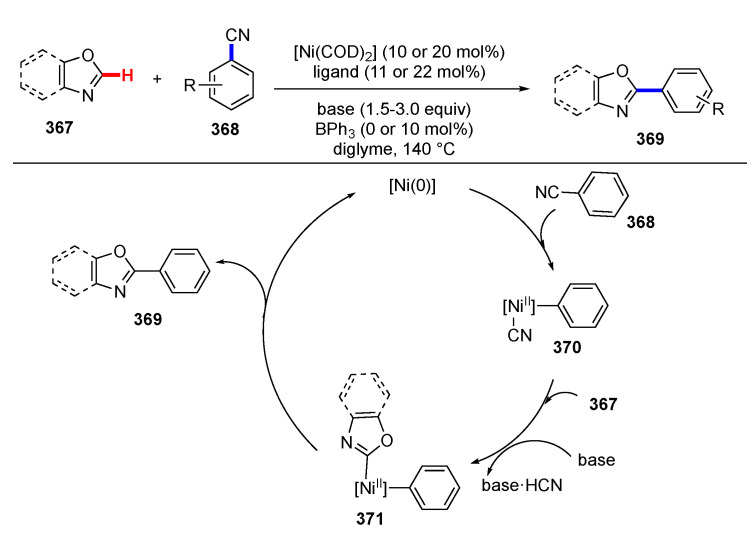
Ni-catalyzed arylation of azoles.

The C–CN bond activation constitutes an interesting approach for the use of nitriles as coupling partners [130]. Kalyani and co-workers reported a protocol for the C–H arylation of azoles **367** utilizing benzonitriles **368** as the arylating reagents (Scheme 48) [131]. The reaction was carried out employing a 20 mol% of [Ni(COD)_2_]/dcype as catalyst. These arylations proceed through the oxidative addition of the benzonitrile to Ni(0) to give the aryl-Ni species **370**, followed by C–H activation of the azole core, and reductive elimination with C–C bond formation.

**Scheme 49:**
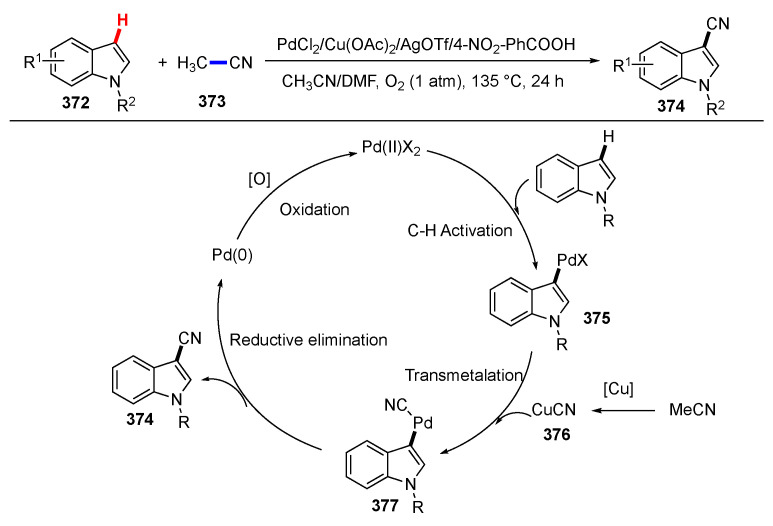
Pd-catalyzed C3–H cyanation of N-protected indoles.

Liu reported an approach for direct C3–H cyanation of *N*-protected indoles utilizing MeCN as the cyanide source (Scheme 49) [132]. The transformation required the use of 5 mol% of PdCl_2_, stoichiometric amounts of Cu(OAc)_2_ and AgOTf as oxidants, along with 1 equiv. of *p*-nitrobenzoic acid, under oxygen atmosphere. Under these oxidative conditions MeCN could form in situ the copper cyanide complex **376** which could transmetallate to a 3-palladated indole intermediate **375** previously formed through C–H activation. The C-3-cyanation of indoles utilizing MeCN had been previously explored by Shen, although in that case the C3–H was halogenated in situ by adding stoichiometric I_2_ to the reaction mixture [133].

**Scheme 50:**
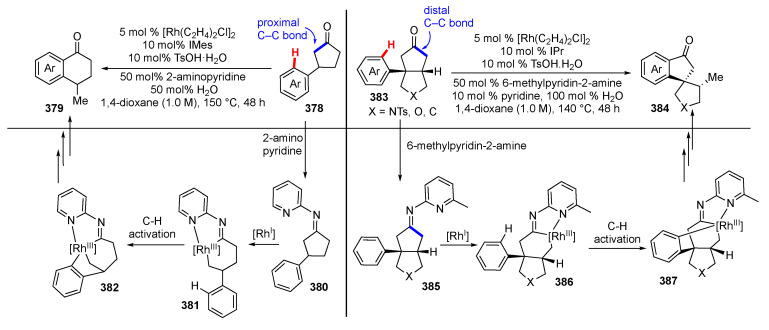
Rh-catalyzed synthesis of cyclopentanones.

A powerful strategy to merge C–H and C–C cleavage relies on the use of properly designed coordinating groups to assist either the cleavage of the C–C or the C–H bond, or both [120]. In 2016, Dong’s group reported the Rh-catalyzed synthesis of tetralones **379** via the selective proximal C–C cleavage of cyclopentanones cores **378** [134]. One year later, a finely tuned 2-amino pyridine ligand enabled them to promote the selective cleavage of the distal C–C bond over the proximal one, hence getting the spiroindanones **384** in good yields from ring-fused cylopentanones **383** (Scheme 50) [135]. The key features of this transformation relay on the transient formation of an imine through the condensation of the ketone with the substituted 2-aminopyridine. The imine group in **385** serves as a temporal directing group to promote the C–C activation by Rh(I) which can further promote the C–H activation of the neighbour aryl ring to form the intermediate **387**. Protodemetalation of the initial alkyl-Rh bond and reductive elimination with formation of the aryl–acyl bond renders the spirocyclic derivative **384**. The distal C–C cleavage selectivity is due to the higher steric hindrance exerted by the 6-substituted pyridine ligand in the transition state.

**Scheme 51:**
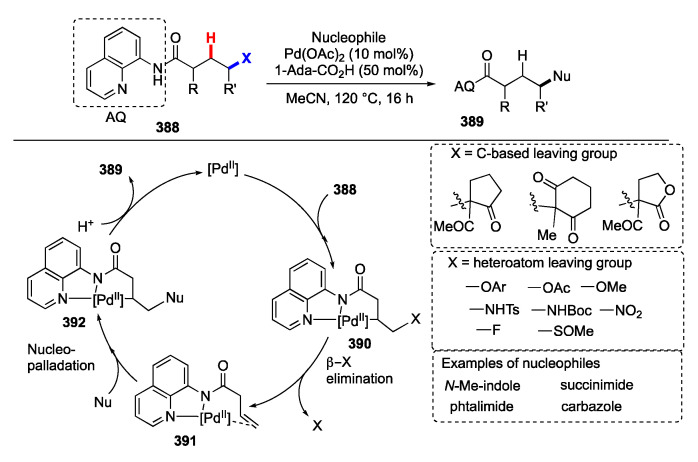
Pd-catalyzed remote C–C functionalization through C–H activation.

In 2018 Engle and collaborators developed an interesting approach for the functionalization of strong Csp^3^–heteroatom and Csp^3^–Csp^3^ bonds by means of a Pd-catalyzed Csp^3^–H activation/β-group elimination cascade (Scheme 51) [136]. The procedure involved the use of 8-aminoquinolyl amide substrates **388** bearing a heteroatom-based or carbon-based leaving group at the γ-position of the aliphatic chain, 10 mol% of Pd(OAc)_2_, and 50 mol% of 1-Ada-COOH, along with the presence of a nucleophile such as *N*-methyl indole. The authors took advantage of the good ability of 8-aminoquinolyl amide directing group to promote a Csp^3^–H palladation, rendering the *C*,*N*,*N*-pincer type intermediates **390**. Under the reaction conditions, the β-carbon elimination of a carbon-based leaving group containing two carbonyl moieties could take place to render the alkenyl derivative **391**. The π-coordination of the olefin to Pd(II) promoted its activation towards the nucleophilic attack of *N*-methylindole, giving a new *C*,*N*,*N*-pincer Pd(II) complex **392**, which in turn could undergo a protodepalladation step to render the functionalized product and the active Pd(II) catalytic species. Albeit the yields obtained with the substrates bearing C-based leaving groups were lower compared to other heteroatom-based ones, the overall process highlight the activation of sterically hindered C–C bonds in unstrained systems.

**Scheme 52:**
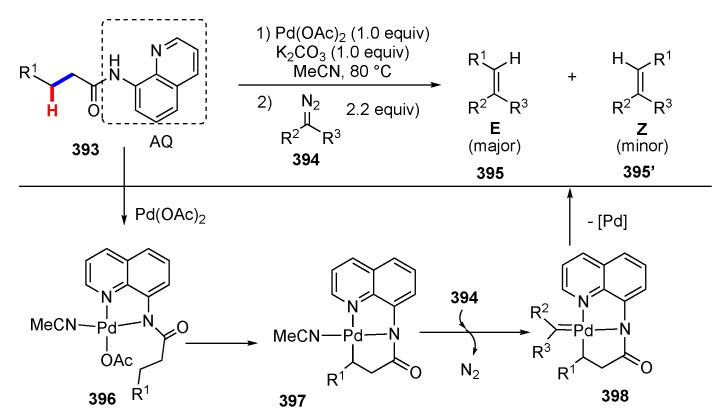
Pd-mediated C–H and C–C functionalization.

Related to Engle’s work, a stoichiometric study on the Pd-mediated Csp^3^–H activation/Csp^3^–Csp^3^ bond cleavage on 8-aminoquinolyl amide substrates **393** was reported by Lautens, García-López and co-workers (Scheme 52) [137]. *C*,*N*,*N*-pincer palladacycles **397**, obtained upon Csp^3^–H activation of substrates **393** by reaction with Pd(OAc)_2_, reacted with carbene precursors such as diazocarbonyl compounds **394**. The migratory insertion of the carbene moiety into the Pd–C bond present in **398** did not lead to the expected β-hydrogen elimination. Instead, the reaction evolved through the scission of the Csp^3^–Csp^3^ bond, hence giving rise to the olefines **395**/**395′**. The inertness nature of the Pd species produced upon the release of the coupling product precluded the development of a catalytic procedure. Mechanistic experiments and DFT calculations showed that the migratory insertion of the carbene and the C–C cleavage might occur in a concerted asynchronous process. Noteworthy, the overall reaction represents the splitting of an unstrained aliphatic chain.

**Scheme 53:**
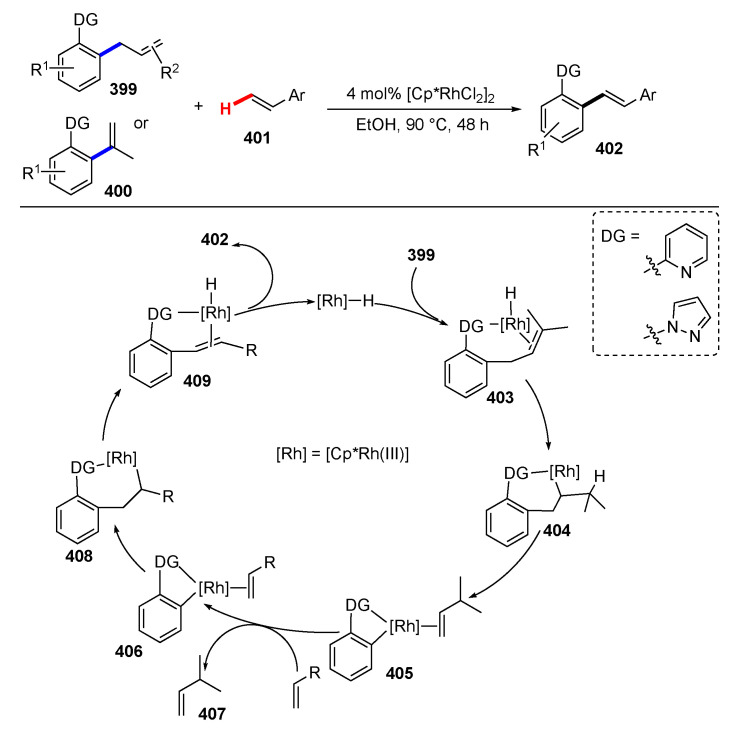
Rh-catalyzed alkenylation of allylarenes. Cp* refers to pentamethylcyclopentadienyl ligand.

Kakiuchi and co-workers described the direct alkenylation of allylarenes **399** bearing a directing group (2-pyridyl or pyrazolyl moieties) in *ortho*-position (Scheme 53) [138]. The reaction was catalyzed by [Cp*RhCl_2_]_2_ and required the use of EtOH as solvent. The process would start with Rh–H, generated in situ from the EtOH solvent. Hydrometalation of the allyl fragment would provide the alkyl species **404**, where a β-C elimination would give the rhodacycle **405**. Exchange of the coordinated olefin by the substrate **401**, migratory insertion into the Rh–C bond, and β-H elimination would be the next steps in the catalytic cycle. In contrast to other Rh-catalyzed reactions where the β-C elimination occurs from alkoxide complexes, in this methodology this step happens directly from alkyl species **404**. This methodology has been extended to the use of alkenylarenes **400** in place of the allyl derivatives **399** [139].

**Scheme 54:**
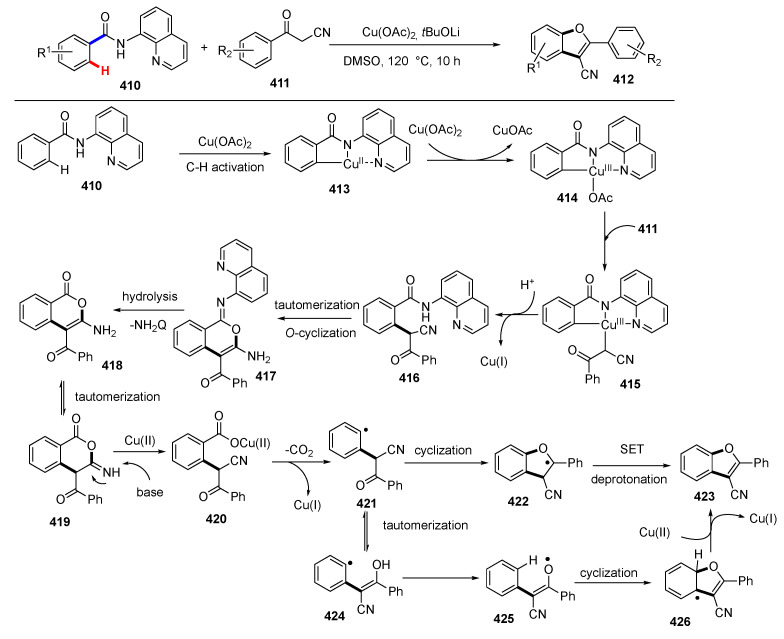
Cu-mediated synthesis of 2-aryl-3-cyanobenzofuranes.

Zhang et al. described a Cu(II)-mediated cascade coupling of benzamides with benzoylacetonitriles to access 2-aryl-3-cyanobenzofuran derivatives **412** (Scheme 54) [140]. The reaction utilized benzamides containing the 8-aminoquinoline moiety as traceless directing group. The proposed mechanism involves the directed *ortho*-C–H metalation of **410** to give the *C*,*N*,*N*-Cu(II) pincer complex **413**. Disproportionation of **413** with an equivalent of Cu(OAc)_2_ would give the Cu(III) intermediate **414**. Benzoylacetonitriles could act as nucleophilic coupling partners, bonding to the metal and undergoing the C–C bond formation to release **416**. A series of tautomerization and hydrolysis events would give the carboxylate **420**, which in turn would undergo the loss of CO_2_ to generate an aryl radical. Further intramolecular cyclization and oxidation would furnish the functionalized benzofuran cores.

**Scheme 55:**
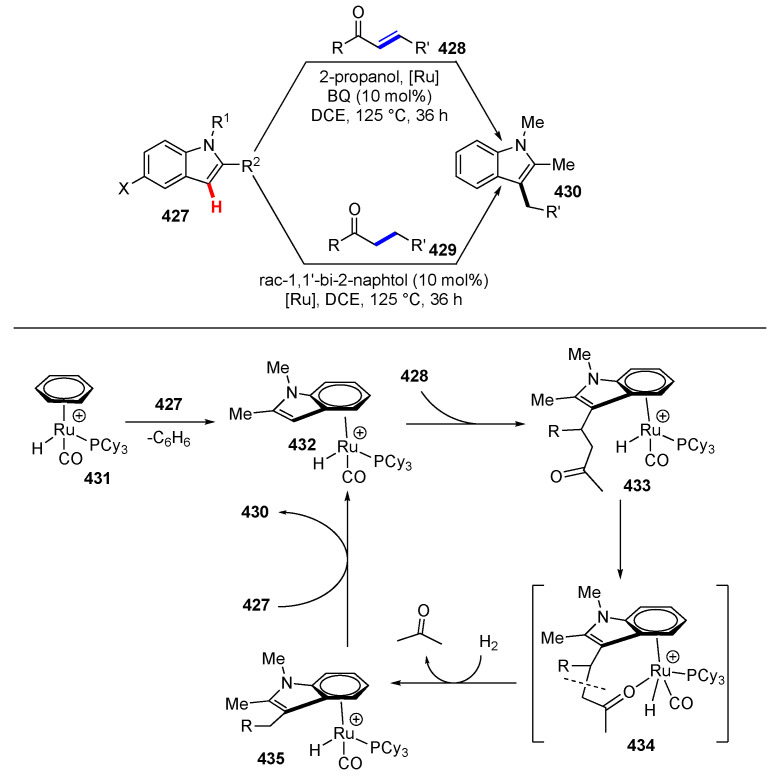
Ru-catalyzed C3–H alkylation of indoles.

In 2020 Yi et al. explored the Ru-catalyzed alkylation of indoles in the C3-position of the ring through the coupling of 1,2-disubstituted indoles **427** and both saturated and unsaturated ketones and aldehydes (Scheme 55) [141]. The process involved the activation of the Cα–Cβ bond present in the carbonyl coupling partner. The experimental conditions required the presence of molecular hydrogen (or isopropanol to generate it in situ) in order to promote the hydrogenolysis of the C–C bond. The authors carried out several deuterium labelling experiments, measured the carbon kinetic isotope effect, and isolated some organometallic intermediates to propose a suitable mechanistic pathway. The reaction would start with the generation of the complex **432** upon ligand exchange in the catalyst **431** with the indole substrate. Then, the conjugated addition of the indole to the enone moiety would produce the alkylation of the indole core at the 3 position. Next, the metal center could coordinate to both the indole and the ketone groups to then promote the hydrogenolysis of the Csp^3^–Csp^3^ bond in **434**. The exchange of the functionalized indole by a new molecule of substrate would re-start the catalytic cycle. Saturated carbonyl substrates could also be utilized in this reaction, since the Ru catalyst could perform an in situ dehydrogenation step prior to the coupling with the indole substrate. Furthermore, no external source of hydrogen is required in this case.

**Scheme 56:**
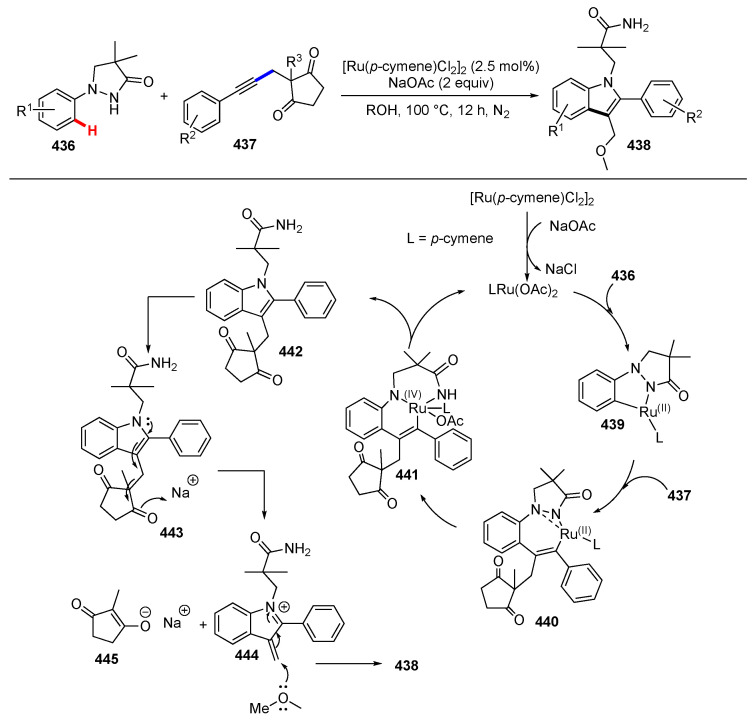
Ru-catalyzed synthesis of 3-(alkoxyalkyl)-1*H*-indoles.

Cui’s group has investigated a Ru-catalyzed synthetic route to 3-(alkoxyalkyl)-1*H*-indoles **438** from pyrazolidinones **436**, 2-acetylenic ketones **437**, and alkyl alcohols (Scheme 56) [142]. 

The mechanistic path starts with the pyrazolidinone-directed C–H metallation, followed by migratory insertion of the acetylenic fragment to give the intermediate **440**. Oxidative addition of the N–N bond to Ru(II) and further reductive elimination afforded the indole core **442**. The cleavage of the C–C bond was assisted by NaOAc, releasing a 1,3-cyclopentadione leaving group. Final nucleophilic attack of the alcohol would provide the functionalized indole scaffolds **438**.

An alternative strategy to merge C–H and C–C cleavage relies on the transition-metal catalyzed functionalization of a C–H bond with an unsaturated coupling partner, to further promote a C–C scission through a retro-Diels Alder, retro-Claisen, retro-aldol or retro-Friedel-Craft reaction.

**Scheme 57:**
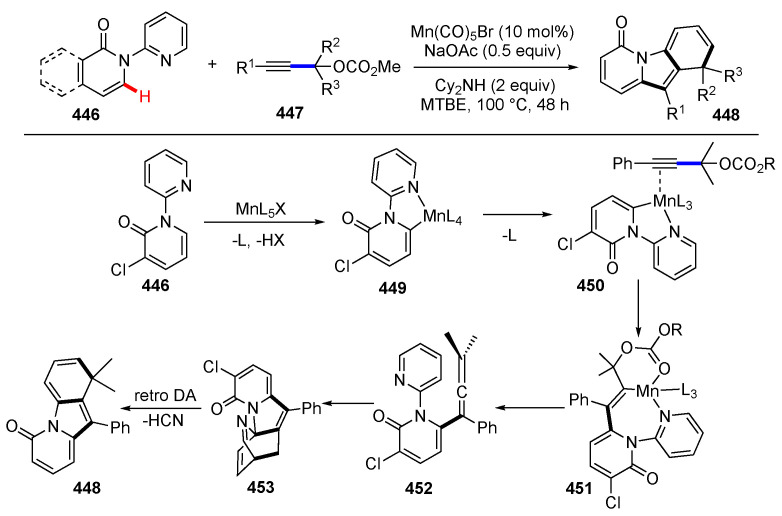
C–H activation/retro-Diels-Alder cascade.

Li and Ackermann simultaneously reported similar conditions for a Mn(I)-catalyzed C–H allenylation of *N*-pyridyl-2-pyridones **446** with propargyl carbonates **447** (Scheme 57) [143,144]. Upon directed metalation, the migratory insertion of the alkyne into the Mn–C bond led to the alkenyl intermediate **451**, which evolved via β-oxygen elimination to give allenyl derivative **452**. Next, intramolecular Diels-Alder reaction involving the pyridine ring, and subsequent C–C cleavage via retro-Diels-Alder furnished the *N*-heterocycles **448**. The approach was also applicable to *N*-pyrimidylindoles. Noteworthy this route utilizes pyridyl and pyrimidyl moieties as transformable directing groups.

**Scheme 58:**
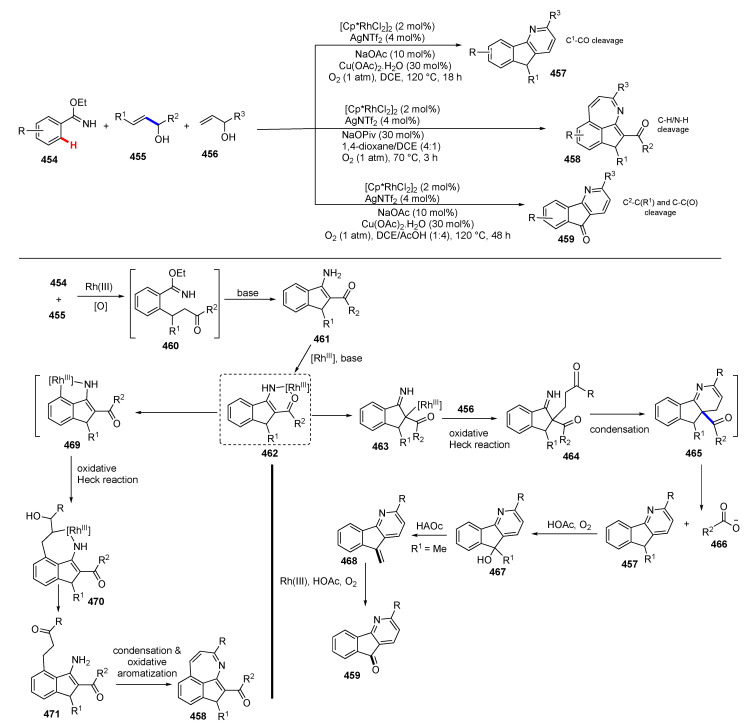
Divergent cascade leading to pyridines, azepines and azafluorenones. Cp* refers to pentamethylcyclopentadienyl ligand.

The Li group reported the synthesis of fused pyridines **457**, azepines **458** and azafluorenones **459** through the Rh-catalyzed coupling of imidates **454** and allylic alcohols (Scheme 58) [145]. The divergent cascade process was controlled by the experimental conditions, while the use of O_2_ as oxidant in 1,4-dioxane/DCE at 70 °C afforded the azepines **458** as a the result of double C–H activation and coupling with allylic alcohols **455**, the presence of Cu(OAc)_2_ at 120 °C afforded the pyridine derivatives **457** as a result of a C–H/C–C cleavage sequence. The proposed mechanism involves the amidate group-directed metalation of **454** and coupling with **455** to give the intermediates **460**, which under the reaction conditions can cyclize to afford the indene species **461**. Then, the coupling with a second molecule of the allylic alcohol and condensation would render the cyclic product **465**. Next, a re-aromatization induced C–C(O) cleavage, or a retro-Claisen reaction renders the fused pyridines **457** and the corresponding carboxylate by-product. Furthermore, if acetic acid was added to the reaction set, the products **457** were oxidized to the azafluorenones **459**.

**Scheme 59:**
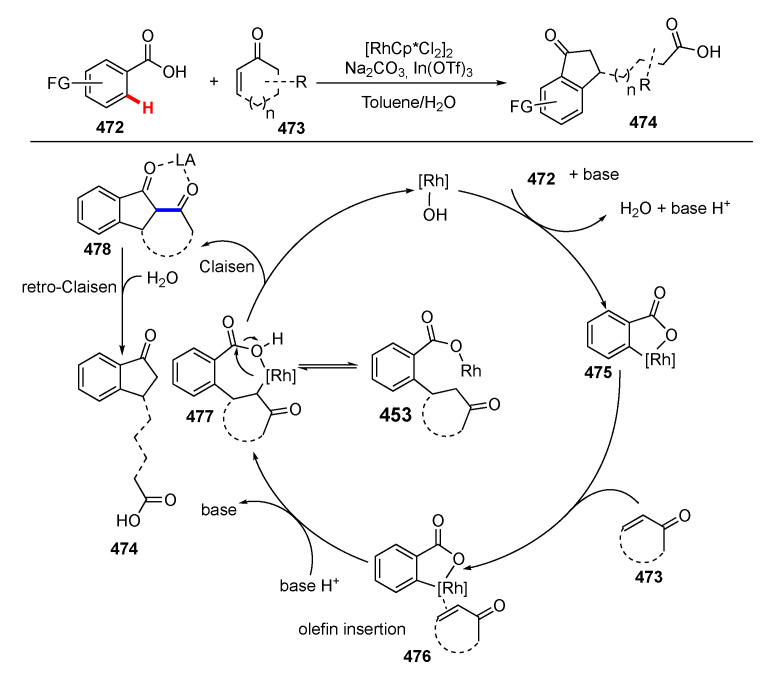
Rh-catalyzed synthesis of indanones. Cp* refers to pentamethylcyclopentadienyl ligand.

Gooßen explored the rhodium-catalyzed annulation of *ortho-* or *meta*-substituted benzoic acids with α,β-unsaturated ketones **473** to give indanone derivatives **474** (Scheme 59) [146]. The process involved the insertion of the olefin into the Rh–C bond of **475**, previously fromed via concerted metalation-deprotonation from the free carboxylic acid **472**. The alkyl-Rh bond present in the intermediate **477** acts as an intramolecular nucleophile to replace the OH group from the acid, rendering the 1,3-dicarbonyl intermediate **478**. The optimized reaction conditions include In(OTf)_3_ which acts as a Lewis acid that promotes a retro-Claisen reaction where the C–C(O) bond is splitted to give the alkylated indanone core **474**.

**Scheme 60:**
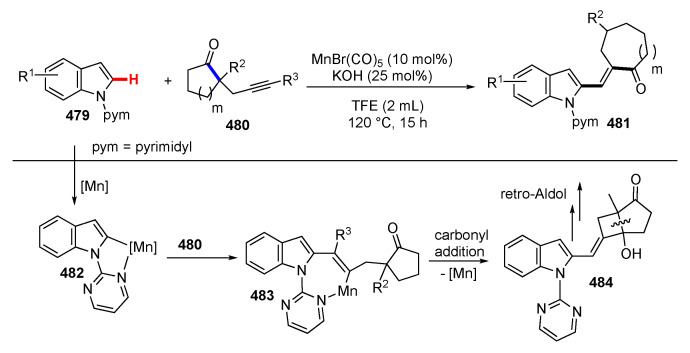
Mn-catalyzed synthesis of medium-size carbocycles.

Li and co-workers reported the synthesis of seven- and eight-membered carbocycles through a Mn(I)-catalyzed C–H activation of *N*-2-pyrimidyl substrates **479** (Scheme 60) [147]. The approach relied on the generation of the metallatated intermediate **482**, which could insert into the alkyne moiety present in the coupling partner **480**. An intramolecular addition to the carbonyl group would afford the cyclobutanol intermediate **484**, which would evolve through a retro-aldol reaction under the experimental conditions, providing the desired carbocycles **481**.

**Scheme 61:**
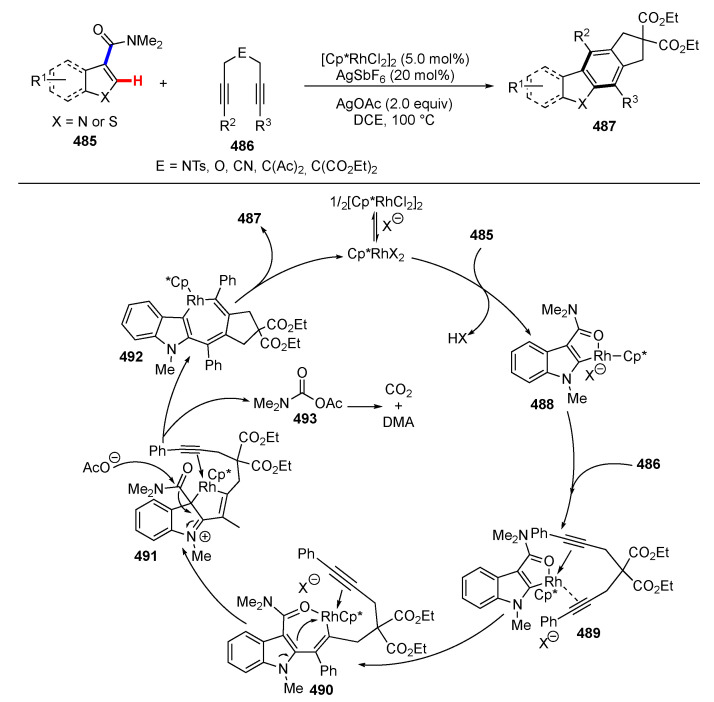
Rh-catalyzed synthesis of cyclopenta[*b*]carbazoles. Cp* refers to pentamethylcyclopentadienyl ligand.

Wang et al. disclosed a Rh(III)-catalyzed route to cyclopenta[*b*]carbazoles **487** through the coupling of 3-amide substituted indoles **485** and 1,3-diynes **486** (Scheme 61) [148]. Building up on the possibility to remove ketone substituents located at the 3-position of indole cores via retro Friedel-Crafts reaction previously described by Shi et al. [149], Wang employed an *N*,*N*-dimethylamide substituent to act as a traceless directing group for the C2–H activation of the heteroaromatic ring and to promote the C3-functionalization through the C–C cleavage of this group. The mechanistic proposal involves the formation of rhodacycle **488** upon C–H activation by cationic Rh(III) species generated in situ from the reaction of [Cp*RhCl_2_]_2_ and AgSF_6_. Next, the coordination of the diyne and migratory insertion of one the triple bonds into the C–Rh bond affords intermediate **490**. Subsequently, the de-aromatization of the indole core would give rise to the *C*,*C*-rhodacycle **491**. Retro-Friedel-Craft reaction with an acetate anion allows the re-aromatization of the heteroarene and the C–C cleavage process, leading to a new organometallic intermediate where the remaining tethered alkyne moiety can undergo migratory insertion. Finally, reductive elimination would form the cyclopenta[*b*]carbazoles **487** and Rh(I). The use of 2 equiv. of AgOAc is necessary to restore the active Rh(III) species.

**Scheme 62:**
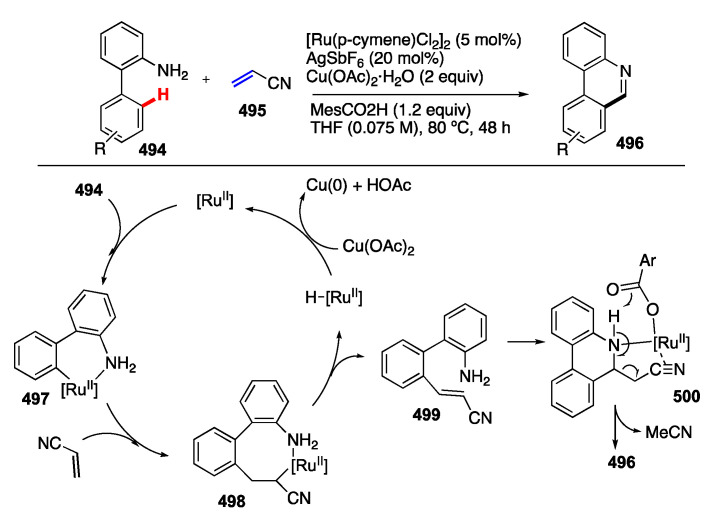
Ru-catalyzed synthesis of phenanthridines through MeCN elimination.

The synthesis of phenanthridines **496** by means of a Ru-catalyzed coupling of 2-phenylaniline **494** with acrylonitrile was reported by Baidya et al. (Scheme 62) [150]. The process relied on the amino group directed C–H metalation, with subsequent olefin insertion to give an alkenyl derivative **499** that, under the reaction conditions, would suffer an intramolecular Michael addition to produce the intermediate **500**. The authors proposed that the coordination of the Ru catalyst to heterocyclic intermediate might facilitate a carboxylate-assisted deprotonation with MeCN elimination to furnish the observed 6-unsubstituted phenanthridines **496**.

**Scheme 63:**
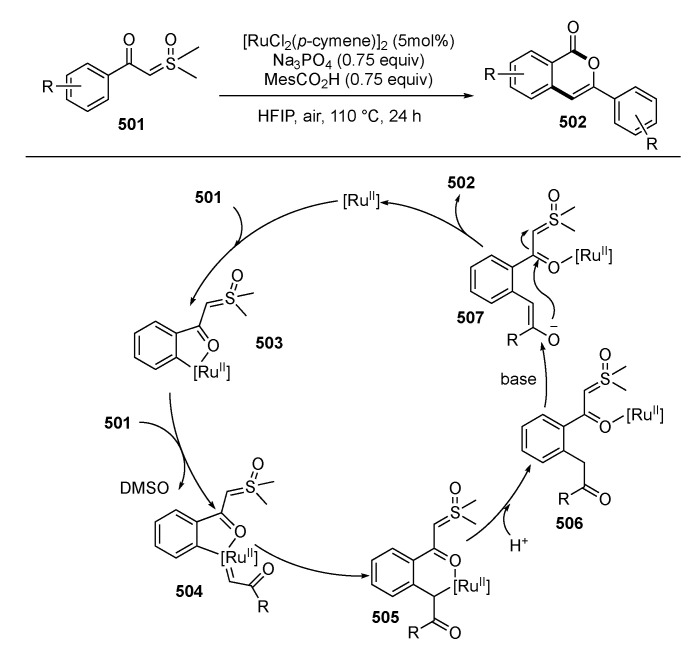
Ru-catalyzed synthesis of isocoumarines.

Cheng’s and Zhou’s respective groups independently described the synthesis of isocoumarines **502** through the Ru-catalyzed C–H/C–C activation of sulfoxonium ylides **501** (Scheme 63) [151,152]. The proposed path starts with the ketone-directed C–H metalation followed by trapping of the resulting metallacycle **503** by a second molecule of sulfonium ylide. The release of DMSO would provoke the generation of a carbene within the coordination sphere of the metal. Next, migratory insertion and protodemetalation would render the *ortho*-alkylated intermediate **506**. Under the basic reaction conditions, an intramolecular cyclization results in the scission of the ylide C–C bond and the formation of the isocoumarin core **502**.

**Scheme 64:**
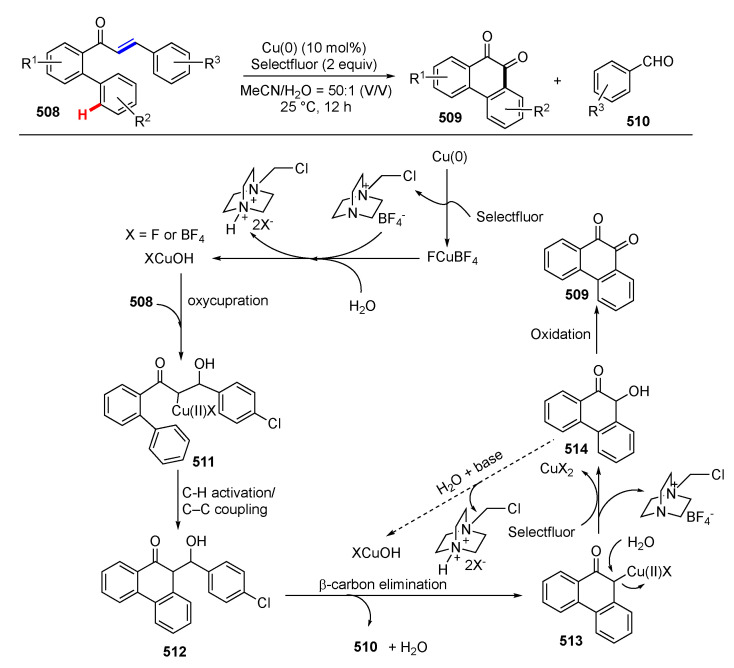
Cu-catalyzed synthesis of 9,10-phenanthraquinones.

Liu et al. disclosed the unexpected route to 9,10-phenanthraquinones **509** as the result of the reaction of *o*-aryl chalcones **508** with 10 mol% of metallic copper and 2 equivalents of Selectfluor in a MeCN/H_2_O mixture (Scheme 64) [153]. The proposed mechanism involves the oxycupration of **508** carried out by Cu–OH species generated in the reaction mixture. A C–H activation on the *o*-aryl group and subsequent C–C coupling would afford the species **512**, where a β-C elimination followed by sequential oxidative steps would furnish the 9,10-phenanthraquinones **509**. Experiments with ^18^O labelled water proved that this was the source from where oxygen incorporation to the organic skeleton took place.

**Scheme 65:**
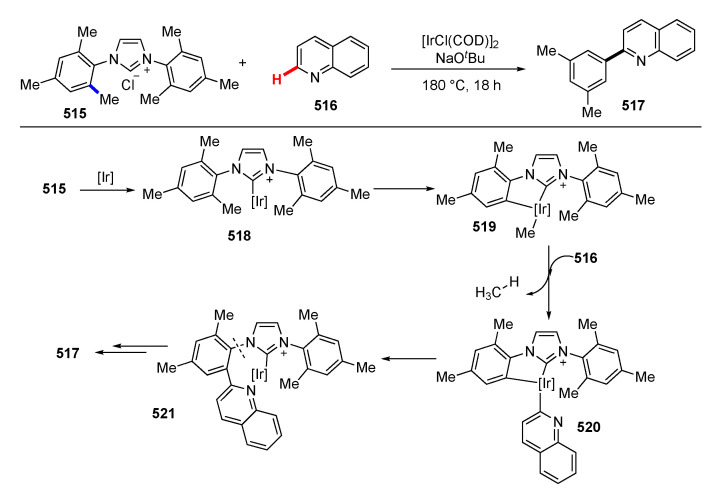
Ir-mediated arylation of heterocycles.

Tobisu’s group found that the reaction of *N*-heterocycles such as quinoline or quinoxaline with stoichiometric amounts of [IrCl(COD)]_2_ and imidazolium salts **515** led to the formation of the 2-arylated heterocycles **517** (Scheme 65) [154]. The reaction involved the cleavage of the C–N and C–C bonds present in the imidazolium ligand. The proposed path implies the oxidative addition of the Me–Ar bond to Ir(I) to give the intermediate **519**, which could then perform the C–H activation of the heterocycle. Reductive elimination would give rise to the complex **521**, which further and not yet completely elucidated evolution would provide the 2-arylated products **517**.

### 3.2. Reactions Involving Radical Intermediates

As in the case of transition-metal catalyzed reactions, functionalized alcohols have served as a versatile platform to implement synthetic methodologies based on the generation of radical intermediates [155,156,157], either through the use of radical precursors or via photochemistry.

**Scheme 66:**
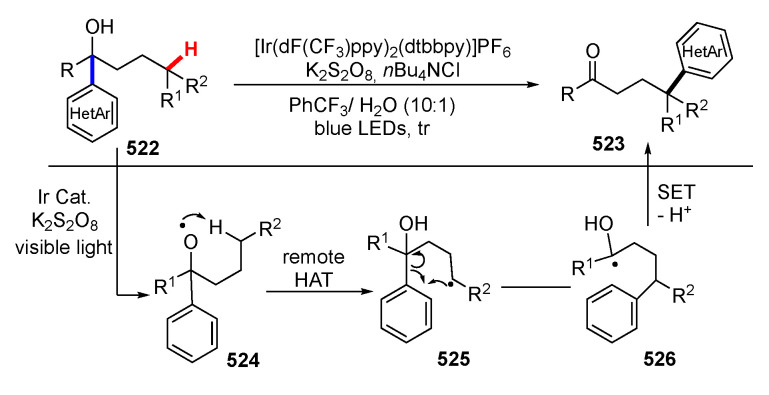
Ir-catalyzed remote C–H arylation.

For instance, Zhu and co-workers developed the heteroarylation of Csp^3^–H bonds located at *γ*-positions in the tertiary alcohol substrates **522** (Scheme 66) [158]. The authors employed Ir[(dF(CF_3_)ppy)_2_(dtbbpy)]PF_6_ photocatalyst, K_2_S_2_O_8_ as oxidant, and *n*Bu_4_NCl additive, in a mixture of PhCF_3_/H_2_O as solvent under blue LED irradiation. The proposed mechanism involves the initial generation of the alkoxyl radical **524** through a proton-coupled electron transfer (PCET) carried out by the oxidized Ir(IV) catalyst at room temperature. Next, 1,5-hydrogen atom abstraction (HAT) would afford the alkyl radical intermediate **525**. This radical would attack to the heteroaryl ring, promoting the homolytic cleavage of the C–C bond which results in the aryl migration and the generation of the radical **526**. Finally, single electron oxidation and deprotonation would afford the γ-heteroarylated ketone. Several heteroaryl rings such as benzothiazole, thiazole or pyridine were studied in the scope of the reaction. The mild conditions to generate the alkoxyl radical compared to other non-photocatalytic methods allowed a broad tolerance to functional groups such as halides, alkenes or alkynes.

**Scheme 67:**
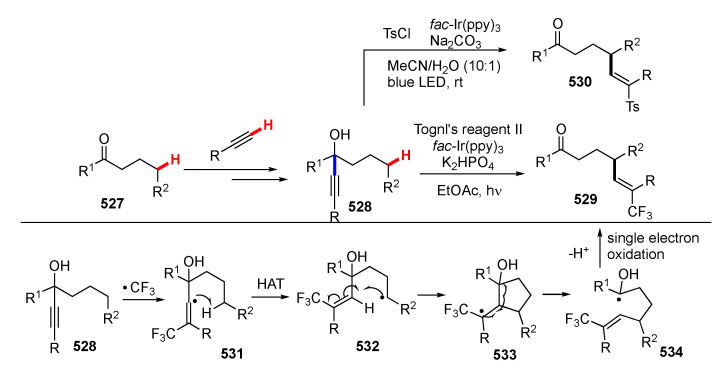
Ir-catalyzed remote C–H functionalization.

The same group further explored the possibilities to combine C–H and C–C functionalization processes in alkynyl tertiary alcohols **528** (Scheme 67) [159]. As in their previous work, they used a photochemical approach to carry out the reaction. However, this time the process did not relay on the generation of an alkoxyl radical but a cabon-centered radical. The synthetic strategy consisted in the photocatalyst-promoted formation of a radical arising from a coupling partner, for instance a CF_3_· radical delivered by Togni’s reagent II, which would add regioselectively to the alkyne moiety, rendering the alkenyl intermediate **531**. This species would evolve through 1,5-HAT giving the new radical intermediate **532** bearing a pending olefine. This step would be favoured given the higher bond energy dissociation of an olefinic Csp^2^–H bond compared to a Csp^3^–H one present in the alkyl group. The double C–C bond can then act as an acceptor of the alkyl radical, producing the cyclopentanol intermediate **533**. Next, the homolytic cleavage of the cyclic Csp^3^–Csp^3^ bond affords the species **534** with the generation of a substituted alkene with *E* configuration. Finally, single-electron oxidation and deprotonation renders the functionalized ketone **529** containing the pending olefine. Other coupling partners to generate the initial radical moiety could be employed under similar conditions, for instance mono- or difluoroalkyl radical precursors such as BrCHFCO_2_Et or BrCF_2_CO_2_Et. Perfluoroalkylsulfinates could also be used in the reaction, but the generation of the corresponding perfluoroalkyl radical was carried out with the assistance of phenyliodide bis(trifluoroacetate) (PIFA).

Zhu extended this photochemical C–H/C–C cleavage of alkynyl tertiary alcohols **528** by utilizing sulfonylchlorides as suitable substrates to generate sulfonyl radicals that could engage in the cascade reaction via similar reaction mechanism to give the derivatives **530** (Scheme 67) [160].

**Scheme 68:**
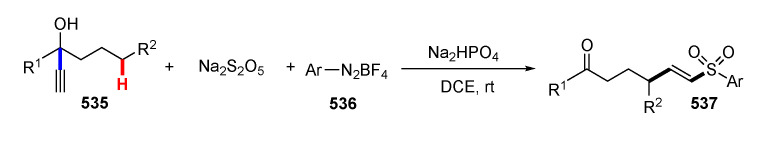
Radical C–H/C-C cleavage leading to sulfone derivatives.

Wu described the synthesis of *E*-vinyl sulfones **537** through 1,5-HAT in the presence of SO_2_ generated from sodium metabisulfite (Scheme 68) [161]. Under the experimental conditions the aryl diazonium salts decomposed to the aryl radicals, which in turn could attack SO_2_ to give a sulfonyl radical. The addition of this radical to the alkyne moiety present in the substrates **535** evolved through 1,5-HAT. Similar evolution as commented above for other radical precursors furnished the functionalized sulfones **537**.

**Scheme 69:**
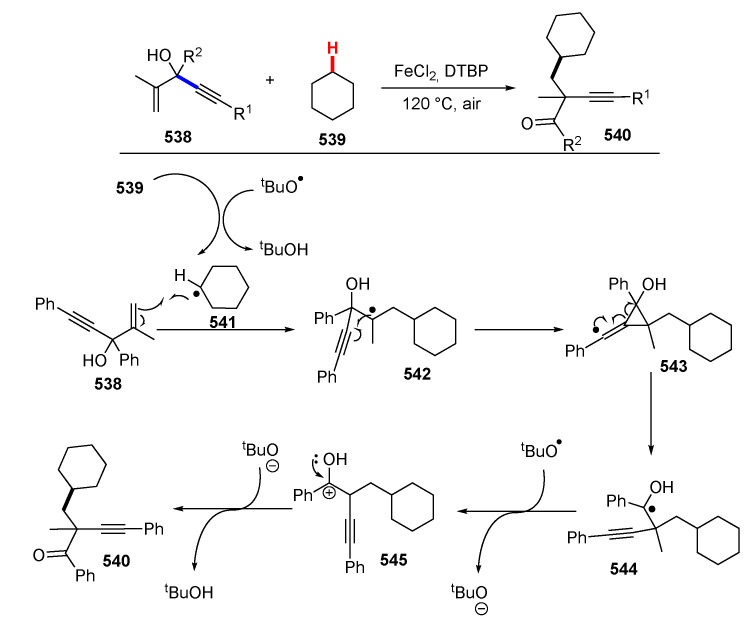
Radical C–H/C–C cleavage leading to functionalized ketones.

Jiang et al. developed the synthesis of α-alkynyl ketones **540** through the 1,2-migration of the alkynyl moiety promoted by a radical pathway (Scheme 69) [162]. The process took place under air at 120 °C, and involved the use of 1,4-alkynyl substrates such as **538**, 10 mol% of FeCl_2_, DTBP (4 equiv) and a cycloalkane acting as both solvent and coupling partner. The reaction would be triggered by Fe(II)-mediated decomposition of DTBP, generating a tBuO· radical which is able to abstract an H atom from the cycloalkyl substrate. Then, the cycloalkyl radical would add selectively to the terminal carbon of the olefin moiety, rendering the new radical intermediate **542**. Next, a 3-*exo-dig* cyclization and subsequent alkynyl migration via Csp^2^–Csp^3^ cleavage leads to the intermediate **544**. Finally, single electron oxidation of this species and deprotonation afford the functionalized ketone **540**. The formation of products **540** was not observed when the reaction was run in the presence of TEMPO as a radical scavenger, supporting a radical pathway for this transformation. Remarkably, 3-*exo*-*dig* cyclizations are considered unfavourable according to Baldwin rules, but in this case the proper design of the substrate forced this type of cyclization due to the initial generation of tertiary radical **542**, more stable than the primary one arising from reverse cycloalkyl radical addition to the olefine moiety present in **538**. Previous work [163] on alkynyl migration of alkynols were limited to 5- or 6-*exo dig* cyclizations.

**Scheme 70:**
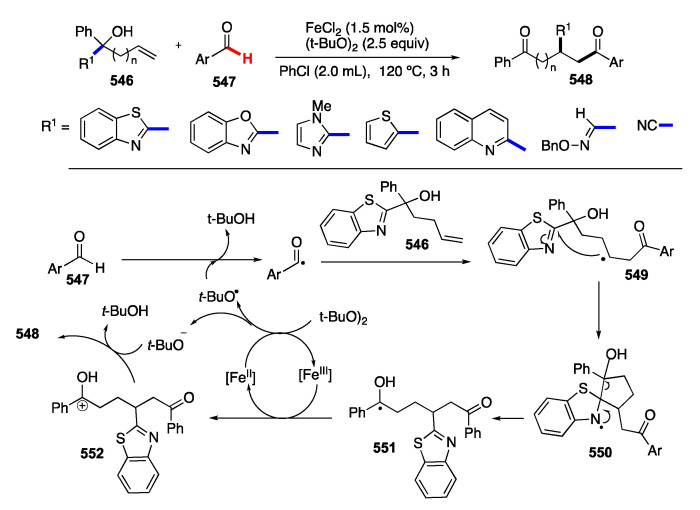
Coupling of olefin-tethered alcohols and benzaldehydes.

A complementary approach to promote the C–C cleavage through radical *ipso*-migration of tertiary alcohols was described by Li and co-workers (Scheme 70) [164]. In this case, acyl radicals generated from benzaldehyde in the presence of FeCl_2_ and di-*tert*-butyl peroxide could evolve through the attack to the tethered olefin moiety in substrates **546**. Similar evolution as described above would produce the heteroaryl ring migration to afford the diketones **548**.

**Scheme 71:**
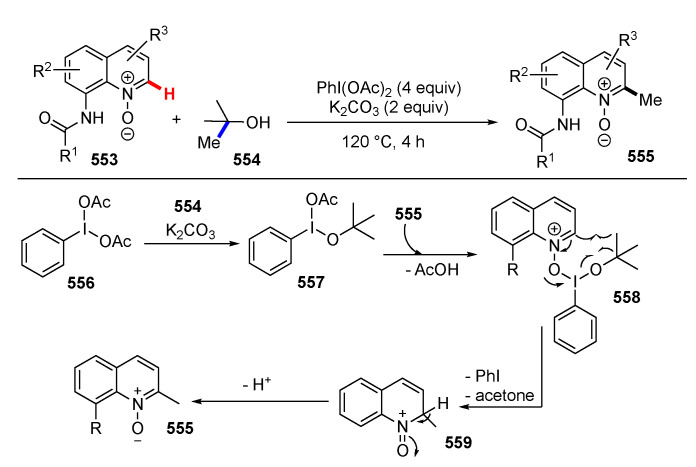
PhI(OAc)_2_-mediated radical alkylation of quinoline *N*-oxides.

Ghosh explored a transition-metal free regioselective alkylation of quinoline *N*-oxides **553** using tertiary and secondary alcohols as alkylating reagents (Scheme 71) [165]. The strategy relied on the oxidative alkyl group migration through C–C bond cleavage promoted by hypervalent iodine reagents such as PhI(OAc)_2_ (PIDA). The proposed pathway starts with the replacement of an acetate group by the alcohol coupling partner. The remaining acetate group on the iodine atom is then replaced by the *N*–oxide group, giving the intermediate **558**. Homolytic C–C cleavage of the alcohol with elimination of a ketone fragment and iodobenzene, followed by deprotonation, furnishes the 2-alkylated product **555**. The *N*-oxide moiety proved crucial for the success of the coupling, since free quinolines did not undergo the alkylation process under similar conditions.

**Scheme 72:**
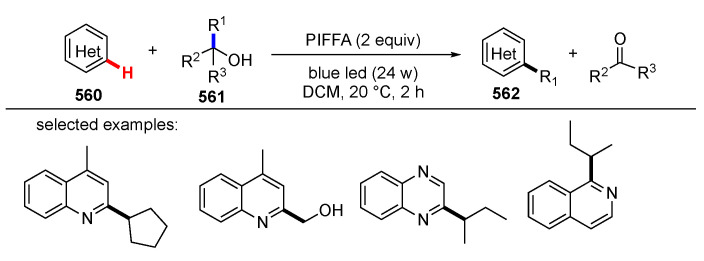
PIFA-mediated radical alkylation of *N*-heterocycles.

Liu and co-workers found that the alkylation of *N*-heterocycles could be carried out not only with tertiary and secondary alcohols, but also with primary ones (Scheme 72) [166]. The key was the use of more electron deficient hypervalent iodine species such as [bis(trifluoroacetoxy)iodo]benzene (PIFA) under blue LED irradiation. This way, the homolytic C–C cleavage could afford the alkyl radical that would further attack the *N*-heterocycle. The scope of this reaction includes sugars and steroids as alkylating agents and a variety of *N*-heterocycles **560** such as quinolines, quinoxalines, phthalazines, or phenathridine among others.

**Scheme 73:**
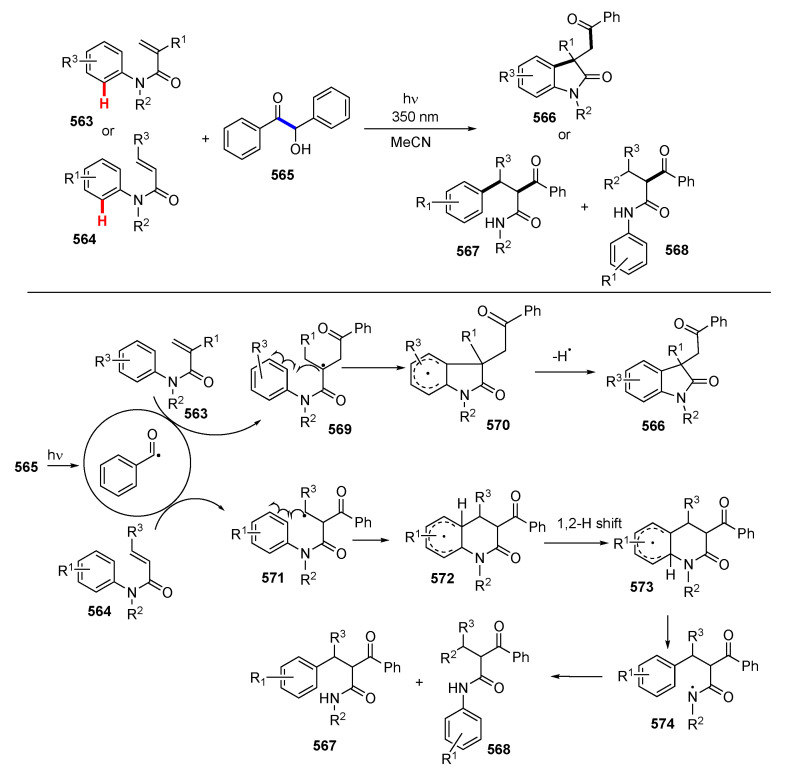
UV-mediated aroyl radical generation from benzoins.

Xia described a UV-light mediated alkene difunctionalization through aroyl radical addition (Scheme 73) [167]. They found that the aroyl radicals generated upon the C–C cleavage of benzoins **565** under UV-light irradiation could be efficiently trapped by alkenes tethered to an aryl group. The outcome of the reaction was highly dependent on the structure of the olefin coupling partner. While acrylamides **563** gave the oxindole derivatives **566** upon attack of the alkyl radical **569** to the aryl ring, the cinnamic amides **564** led to the open-chain amides **567**, through a 1,2-H shift and C–N bond cleavage.

**Scheme 74:**
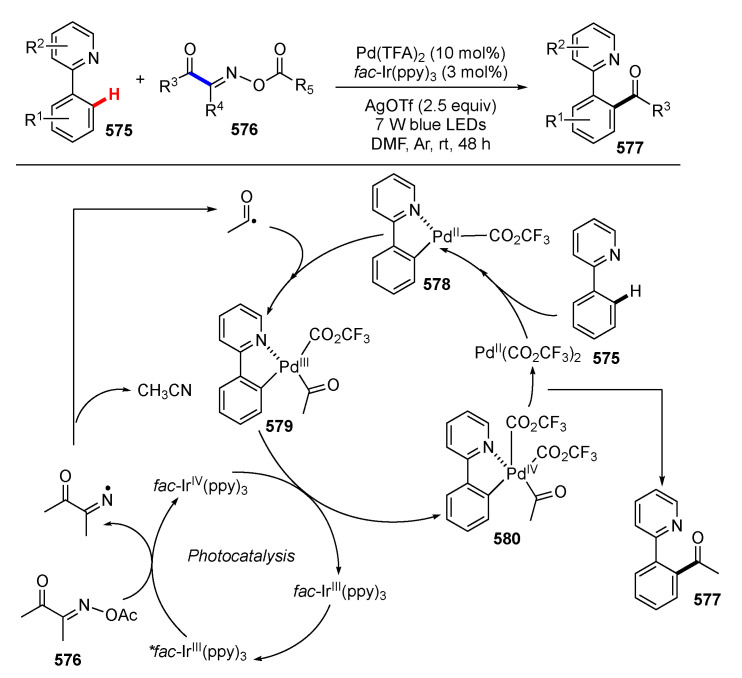
Pd-catalyzed C–H acylation of 2-arylpyridines. The term *fac-Ir^III^(ppy)_3_ refers to the excited state of the photocatalyst.

Parallelly to the use of the strained cyclobutanone oxime precursors of iminyl radicals discussed above, the analogue open chain derivatives have been utilized as building blocks to promote C–C scission along with C–H functionalization. For instance, Chen et al. engaged Ir-photoredox and Pd catalysis for the C–H acylation of 2-arylpyridines **575** [168]. The protocol utilized the ability of *fac*-Ir(ppy)_3_ to generate iminyl radicals from α-keto oxime esters **576**, which in turn generated acyl radicals upon C–C cleavage (Scheme 74). The acyl radicals could be trapped by palladated 2-arylpyridines **579**, previously formed via C–H activation. The resulting Pd(III) species could undergo a SET oxidation by the Ir(IV) oxidized photocatalyst to give a Pd(IV) intermediate. Reductive elimination would render the acylated products **577** and restore the Pd(II) active catalyst.

**Scheme 75:**
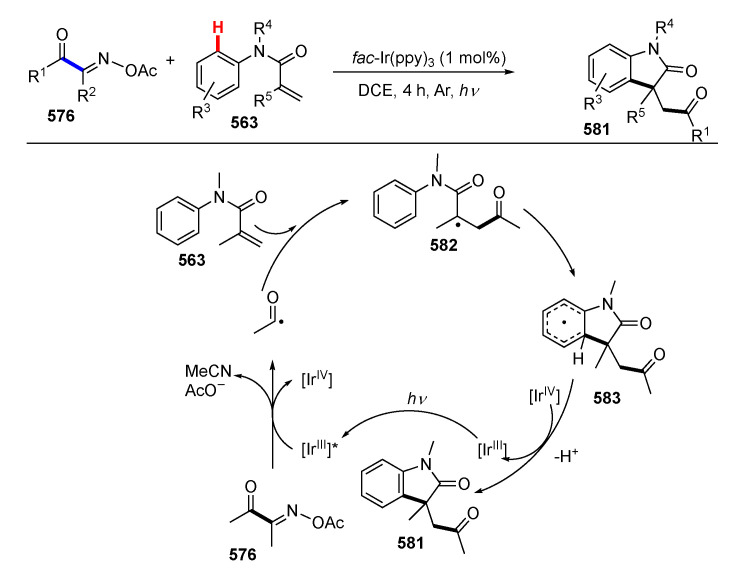
Ir-photocatalyzed synthesis of functionalized oxindoles. The term [Ir^III^]* refers to the excited state of the photocatalyst.

Wu and co-workers studied a related photochemical C–C/C–H functionalization utilizing oxime ester derivatives **576** (Scheme 75) [169]. The presence of a radical acceptor such as acrylamides **563** in the reaction mixture affords the intermediate **582**. The attack of the alkyl radical fragment to the aromatic ring followed by further single electron oxidation and deprotonation gives rise to the alkylation of the C–H bond. The authors proved that oxime esters bearing *O*-benzoic or *O*-acetic moieties were competent to promote the N–O bond cleavage. Moreover, a range of alkyl- and aryl- substituents on the carbonyl group rendered good yields of the coupling products arising from the respective alkyl- or aryl-acyl radicals.

**Scheme 76:**
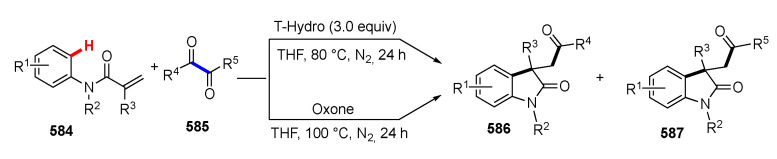
Radical synthesis of functionalized oxindoles from α-diketones.

The Guo’s research group reported the synthesis of oxindole cores bearing a carbonyl group through the addition of acyl radicals to *N*-arylacrylamides (Scheme 76) [170]. The generation of the acyl radical was carried out from the homolytic scission of the Csp^2^–Csp^2^ bond of α-diketones **585**, promoted either by *tert*-butyl hydroperoxide or oxone. Both aryl/aryl or alkyl/alkyl- substituted diketones performed well in the reaction.

**Scheme 77:**
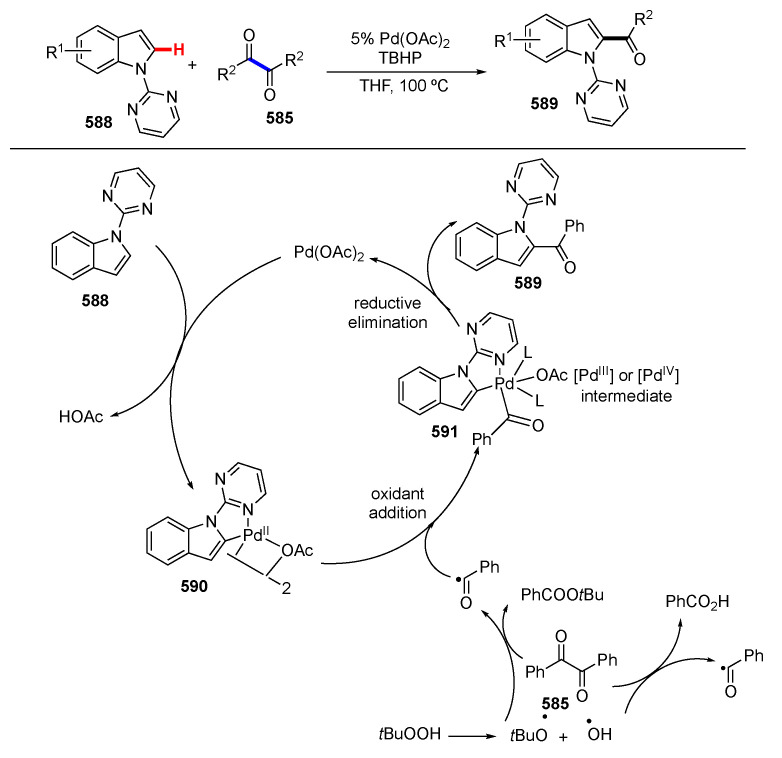
Pd-catalyzed acylation of indoles.

Liu et al. described the C2-acylation of *N*-(2-pyrimidyl)indoles **588** utilizing α-diketones **585** as acylating reagents [171]. The reaction involved the use of 5 mol% of Pd(OAc)_2_ as catalyst and TBHP to promote the homolytic cleavage of the CO–CO bond present in the diketone coupling partner (Scheme 77). The reaction was initiated by the C2–H metalation assisted by the pyrimidyl group. The palladacycle **590** could trap the acyl radical to give a Pd(III) or Pd(IV) intermediate. Finally, the C–C coupling would render the desired 2-acylated indole derivatives.

**Scheme 78:**
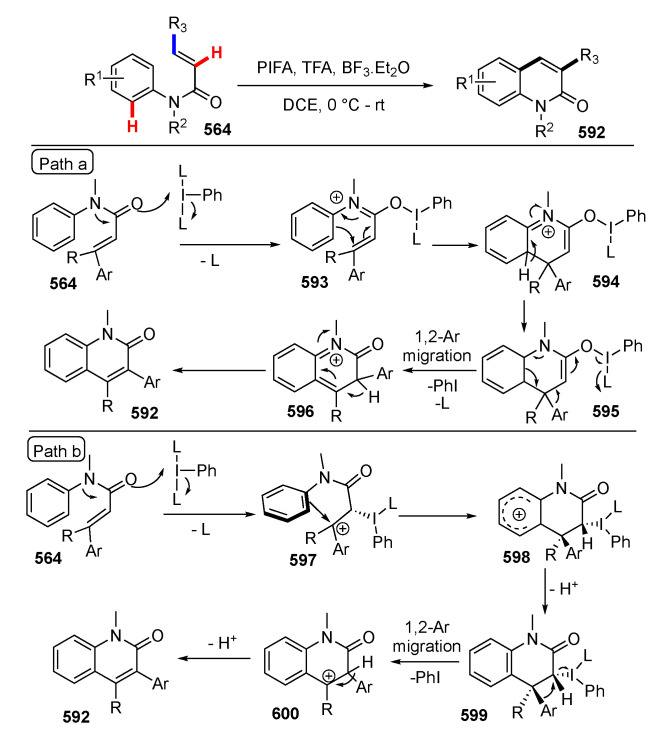
Hypervalent iodine-promoted 3-arylquinolin-2-ones.

Some methodologies relying on radical intermediates exploit intramolecular 1,2-aryl migration processes to promote the homolytic cleavage of C–C bonds. In 2016, Zhao and co-workers [172] deepened on the study of their previously reported work about hypervalent iodine-promoted synthesis of 3-arylquinolin-2-ones from cyclic amides [173]. In this transformation an oxidative annulation involving the C–H bond in *ortho*-position to the amide group in substrates **564**, and a 1,2-aryl migration took place to deliver the structures **592** (Scheme 78).

**Scheme 79:**
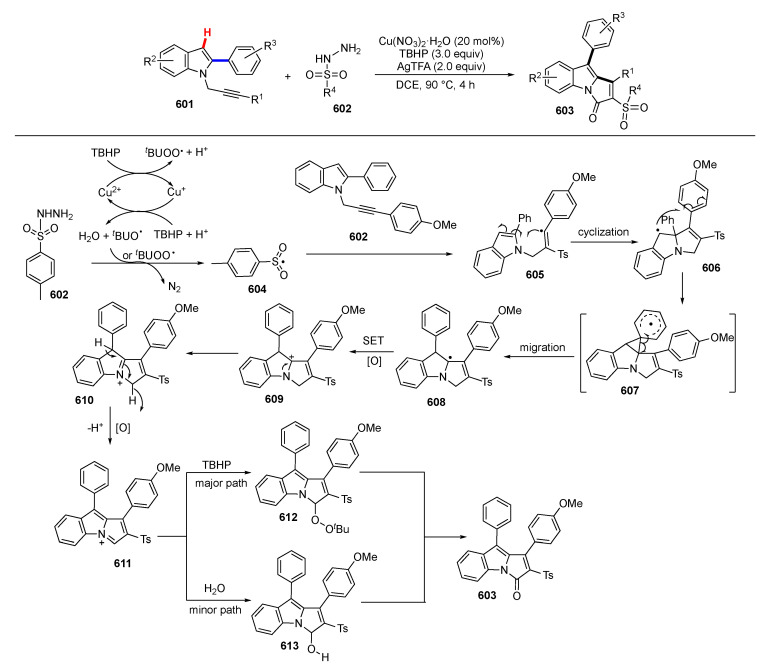
Radical methodology to prepare sulfonated pyrrolo[1,2-α]indole derivatives.

Liang et al. exploited a radical aryl migration strategy for the dual functionalization of a C–H and a C–C bond in *N*-propargylindole substrates **601** (Scheme 79) [174]. The reaction conditions involved the use of TsNHNH_2_, Cu(NO_3_)_2_·3H_2_O, TBHP, and AgTFA in DCE at 90 °C. The decomposition of TBHP promoted by copper would trigger the generation of sulfonyl radicals **604** from the sulfonyl hydrazide starting material. Next, the attack of the sulfonyl radical to the alkyne moiety present in **601** would render the vinyl intermediate **605**, which in turn could undergo an intramolecular cyclization and 1,2-aryl migration. From the intermediate **608**, the reaction evolves through a series of single-electron oxidation and deprotonation processes to give the desired 2-sulfonated pyrrolo[1,2-α]indole derivatives **603**.

**Scheme 80:**
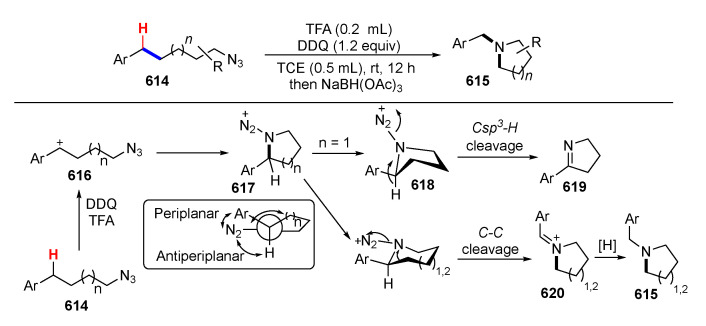
Synthesis of cyclic imines and amines through the intramolecular Csp^3^–H/C–C bond amination.

Jiao et al. reported the synthesis of cyclic imines and tertiary amines through the intramolecular Csp^3^–H/C–C bond amination of alkylazide substrates **614** bearing a pendant aryl group (Scheme 80) [175]. They found that in the presence of DDQ oxidant and TFA, the oxidation of substrates **614** could lead to the corresponding benzyl cation **616**, which could be attacked intramolecularly by the azide moiety. The six-membered ring intermediate **617** undergoes the loss of N_2_ and 1,2-alkyl migration to give the imine cation **620**. Subsequent reduction with NaBH(OAc)_3_ furnished the isolable cyclic tertiary amines **615**. Noteworthy, the alkyl migration was only observed when a six-membered-ring transition state was formed, shorter or longer alkyl chains were not suitable for this transformation.

**Scheme 81:**
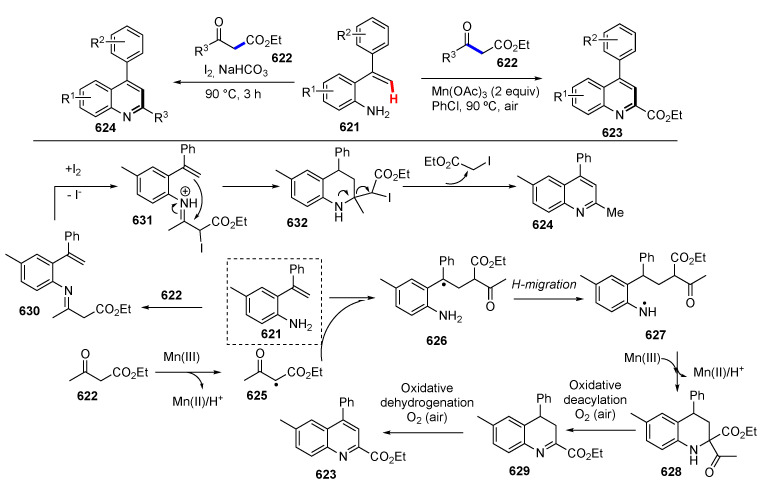
Divergent synthesis of quinoline derivatives.

Iodine derivatives have proven to be useful reagents to induce transformations involving C–H/C–C cleavage. Zhang et al. described two different routes to access substituted quinolines from 2-styrylanilines **621** and β-ketoesters **622** by the use of two different sets of experimental conditions (Scheme 81) [176]. When the reaction was carried out in the presence of molecular iodine and NaHCO_3_, the alkyl or aryl derivatives **624** were obtained via formation of the imine **630**. Nevertheless, the use of Mn(OAc)_3_ promoted a radical path through the abstraction of a hydrogen atom from the β-ketoester unit, delivering the compounds **623**.

**Scheme 82:**
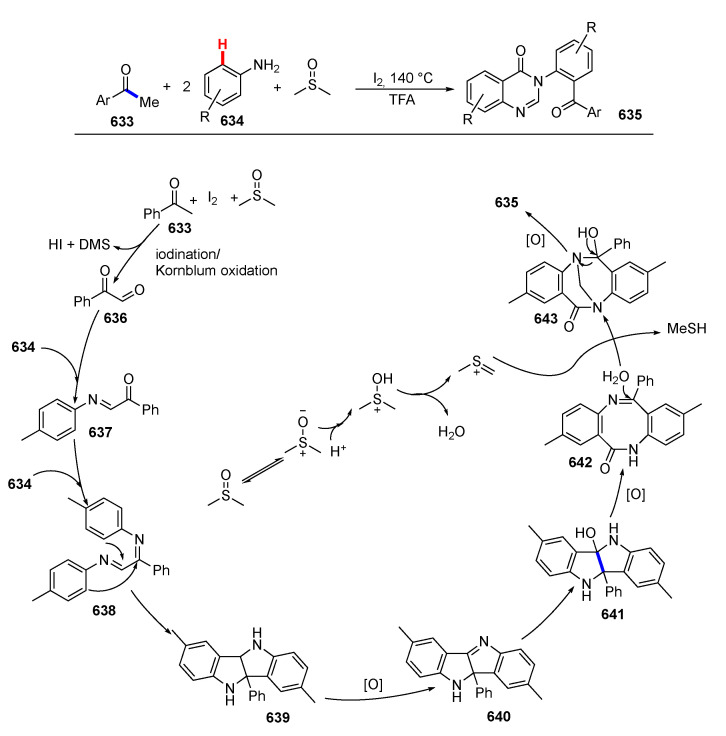
Oxidative coupling of aniline and acetophenone derivatives.

Wu and co-workers explored the synthesis of arylquinazolines **635** through the oxidative coupling of anilines and acetophenones (Scheme 82) [177]. The reaction required one equivalent of iodine and DMSO as solvent at 140 °C. Under such conditions, the methyl ketones are converted into phenylglyoxal **636** through an iodination/Kornblum oxidation. The intermediate **636** would undergo the condensation with aniline to form a diimine **638**, in which a series of intramolecular cyclization/hydration processes result in the functionalization of the *ortho*-C–H of aniline and splitting of the C(CO)–Me bond of the acetophenone starting material.

**Scheme 83:**
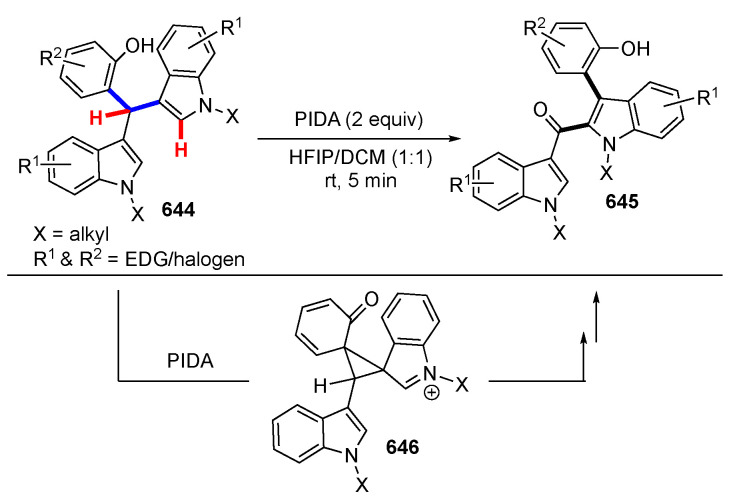
PIDA-mediated rearrangement of unstrained diindolylmethanes.

Lankalapalli described a PhI(OAc)_2_-mediated rearrangement of unstrained diindolylmethanes **644** in which two C–C and C–H bonds were cleaved (Scheme 83) [178]. The strategy relied on the oxidative dearomatization of the phenol moiety carried out by the hypervalent iodine reagent. From the intermediate **646** a series of aryl migration and cyclization steps gave rise to the 2,3′-diindolylketones **645**.

### 3.3. Reactions Promoted by Lewis or Brønsted Acids or Bases

**Scheme 84:**
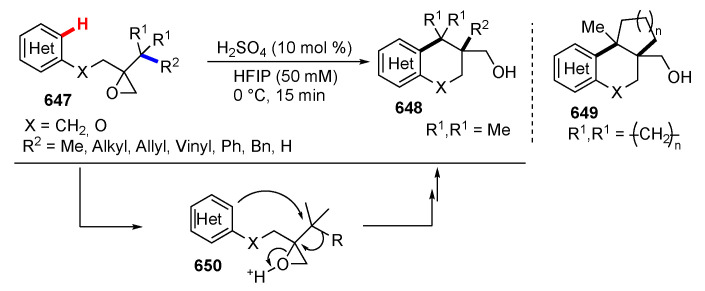
Sulfuric acid-catalyzed cycloisomerization of neopentylic epoxides.

As happened in the case of the strained systems, it is possible to perform the functionalization of C–H bonds along with the cleavage of unstrained C–C bonds in the presence of Lewis or Brønsted acids, or basic conditions. Recently, Magauer et al. established an acid-catalyzed route to tetralin and chromane derivatives **648** through the cycloisomerization of neopentylic epoxides **647** (Scheme 84) [179]. The presence of 10 mol% of H_2_SO_4_ in HFIP promoted the opening of the epoxide ring with subsequent 1,2-migration of the neighbouring alkyl group. Next, the aryl ring present in the substrate could act as a nucleophile for the Friedel-Crafts termination step, rendering the cyclic organic framework. When the substrate contained a cyclobutyl or cyclopentyl moiety in α-position to the epoxide, the reaction provoked the ring expansion through the alkyl migration, rendering tricyclic scaffolds **649**. Remarkably, the overall process set two vicinal all-carbon centers in the corresponding tetraline or chromane cores.

**Scheme 85:**
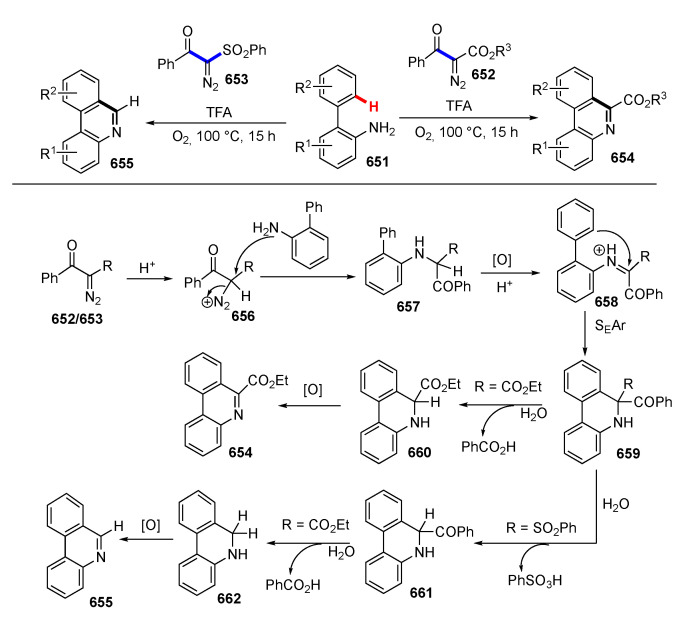
Synthesis of phenanthridines via C–H/C–C cleavage.

A trifluoroacetic acid-promoted [5 + 1] annulation of diazocompounds **652** or **653** and 2-arylanilines **651** leading to phenathridine cores was described by Szostak and collaborators (Scheme 85) [180]. The outcome of the reaction was controlled by the substitution pattern present in the diazocompounds. Under acidic conditions, **652**/**653** could undergo a protonation to deliver the species **656**. The nucleophilic attack of the amino group of **651** to **656** with N_2_ elimination would produce the secondary amine **657**. The oxygen atmosphere in which the reaction was carried out promoted the oxidation of **657** to give the imine **658**, that could evolve through an electrophilic attack to the aromatic ring to render the cyclized intermediate **659**. When the substituents of the diazocompound are -CO_2_Et and -COPh, a selective de-acylation and oxidation takes place to give the substituted phenanthridine derivative **654**. However, when they bear -COPh and -SO_2_Ph groups, a consecutive de-sulfonylation/de-acylation/oxidative aromatization furnishes the phenanthridines unsubstituted at the 6-position **655**.

**Scheme 86:**
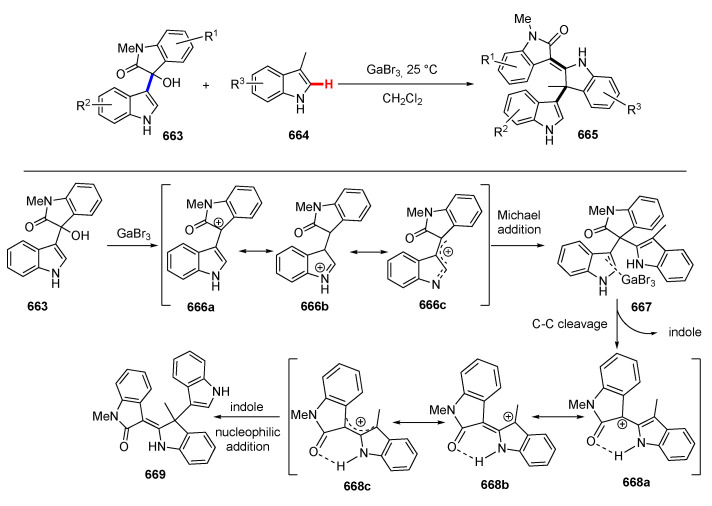
GaBr_3_-catalyzed synthesis of 3,3′-bisindole derivatives.

Shi described the synthesis of complex 3,3′-bisindole derivatives via gallium-bromide-promoted dearomative indole insertion in 3-indolylmethanols **663** (Scheme 86) [181]. The proposed mechanism relays on the initial generation of the delocalized cation intermediate **666** due to the strong Lewis character of Ga(III). GaBr_3_ would also favour the C–C cleavage step, upon the nucleophilic attack of 3-methyl-indole coupling partner.

**Scheme 87:**
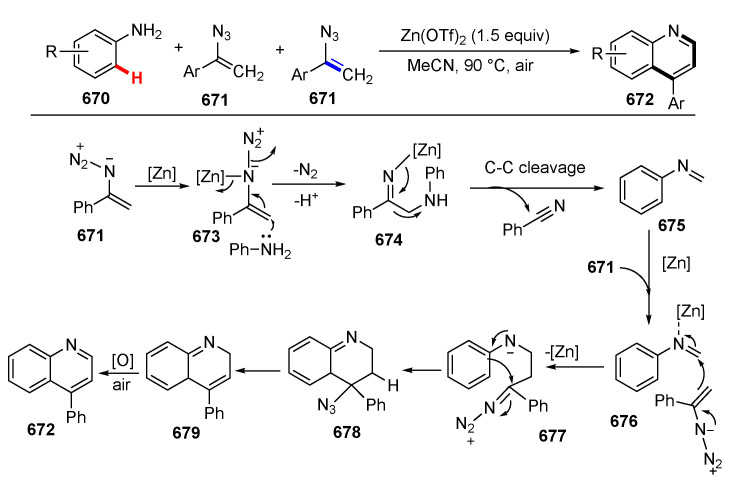
Zn(OTf)_2_-Catalyzed synthesis of 4-substituted quinolones.

Jiang disclosed a multicomponent reaction of vinylazides **671** and anilines promoted by Zn(OTf)_2_ leading to 4-substituted quinolines **672** (Scheme 87) [182]. The proposed mechanism stated the coordination of the Zn(II) to the azide moiety, facilitating the nucleophilic attack of aniline to give the intermediate **674** with loss of N_2_. The C–C bond cleavage produces the imine **675** with the elimination of benzonitrile as by-product. A [4 + 2] annulation of **675** with another molecule of the azide **671**, followed by loss of HN_3_ and oxidation under air furnish the 4-arylated quinolines **672** in moderate to good yields.

**Scheme 88:**
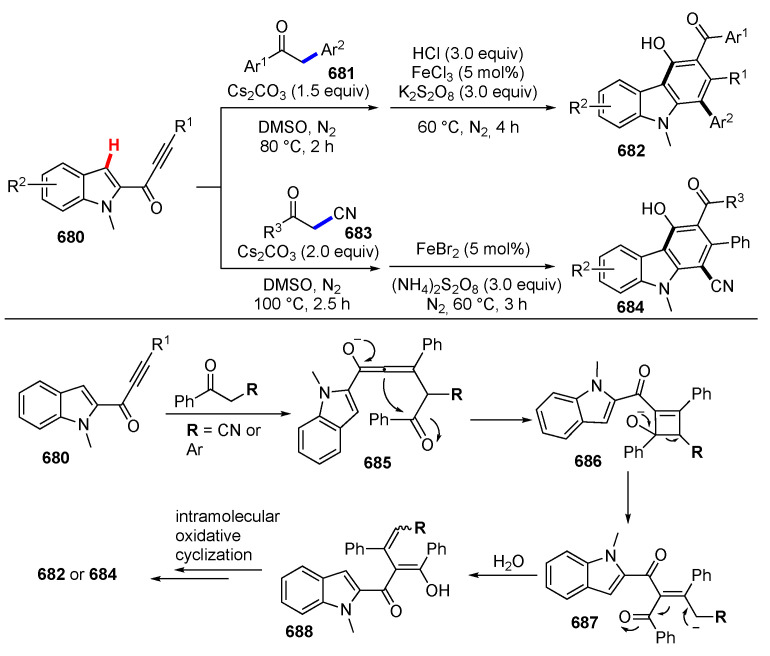
Base-mediated synthesis of carbazoles.

Li et al. developed a C–C/C–H bond cleavage approach to functionalized carbazoles by combining a base-promoted C–C scission with an iron-catalyzed skeletal rearrangement (Scheme 88) [183]. The authors described a one-pot two steps process in which the initial coupling of 1-(*N*-methyl-2-indolyl)alkynyl ketones **680** with α-aryl ketones rendered the derivatives **682**. In the first step the generation of a cyclobutanol intermediate **686** was key. The consecutive FeCl_3_-catalyzed cycloisomerization step furnished the desired carbazole **682**. The same authors expanded the scope of this transformation to cyano-substituted carbazoles **684** by employing α-cyano ketones **683** [184].

**Scheme 89:**
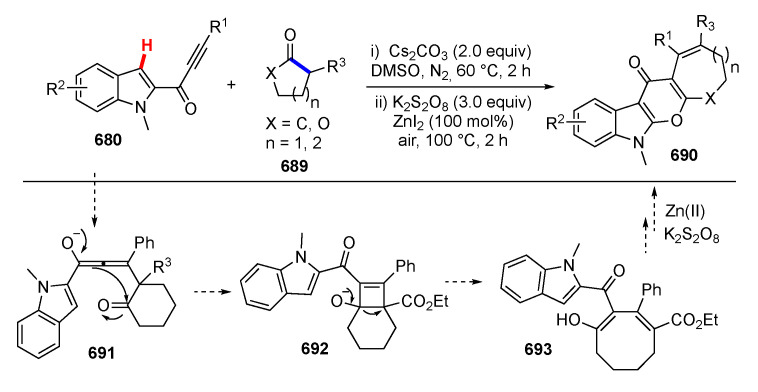
Base-mediated synthesis of pyranoindolones.

The use of 2-oxo-cyclohexanecarboxylate as coupling partner in this Cs_2_CO_3_-mediated methodology and a subsequent step in the presence of one equivalent of ZnI_2_ and K_2_S_2_O_8_ under air allowed the synthesis of pyranoindolones **690**, bearing a fused medium-size carbocyclic ring (Scheme 89) [185].

## 4. Reactions Involving Triple Bond Scission

There are some reports where the scission of triple C≡C bonds, generally under oxidative conditions, accompanies the functionalization of a C–H bond. Recent progress in this type of reactions is summarized below.

**Scheme 90:**
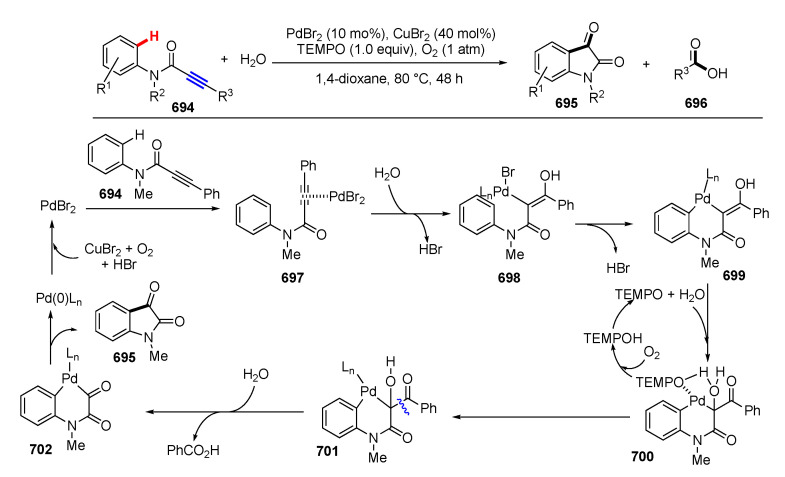
Pd-catalyzed synthesis of indoline-2,3-diones.

Li and co-workers obtained indoline-2,3-diones **695** through the C–H functionalization and the cleavage of the triple C≡C bond present in *N*-arylpropiolamides **694** (Scheme 90) [186]. The oxidative cyclization takes place in the presence of PdBr_2_ (10 mol%) as the catalyst, and a mixture of oxidants such as CuBr_2_ (40 mol%), TEMPO and O_2_ in 1,4-dioxane at 80 °C. The reaction would be initiated by the alkyne activation through the π-coordination to Pd(II), which would promote the nucleophilic attack of H_2_O, giving rise to the alkenyl-Pd intermediate **698**. Next, C–H activation on the aromatic ring would lead to the *C*,*C*-palladacycle **699**. Subsequently, a Wacker oxidation reaction with another molecule of water assisted by TEMPO would deliver the intermediate **701**. The C–C bond in this intermediate is cleaved in the presence of another molecule of water to give the new palladacycle **702** and the corresponding carboxylic acid. Reductive elimination in **702** would afford the desired indoline-2,3-dione and Pd(0). The role of CuBr_2_ and O_2_ would be the re-oxidation of Pd(0) to Pd(II). Mechanistic experiments carried out with ^18^O labelled water corroborated the role of water as coupling partner since both the heterocycles **695** and the side-product **696** contained ^18^O atoms.

**Scheme 91:**
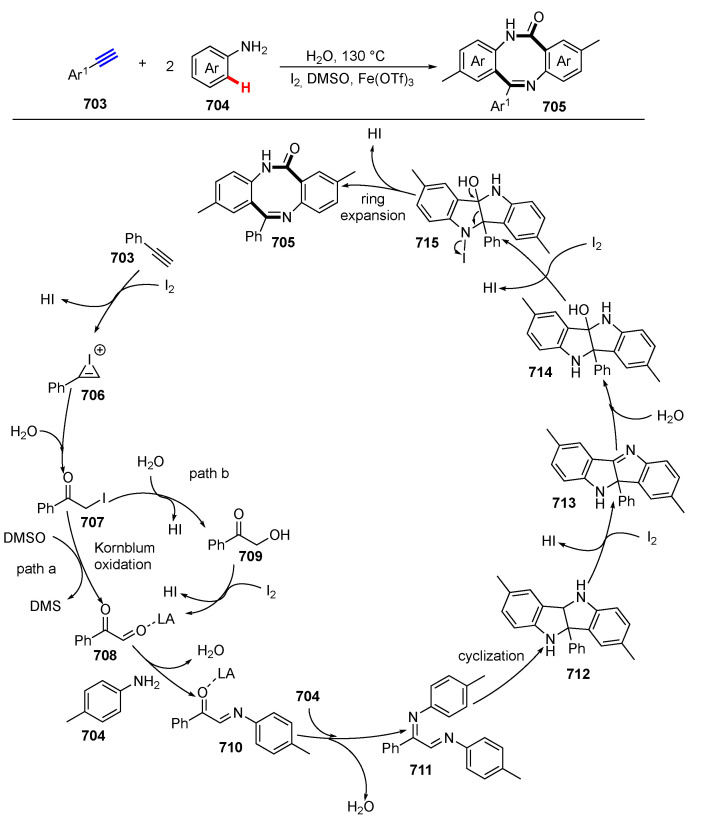
Synthesis of eight-membered heterocycles through C–H/C–C functionalization.

Wu et al. reported the synthesis of eight-membered *N*-heterocycles **705** utilizing arylacetylenes as one-carbon synthons (Scheme 91) [187]. 

This work is closely related to the reaction described in the Scheme 82, though replacing acetophenone by arylacetylenes. Indeed, the mechanism relies in the formation of an arylglyoxal through a Kornblum oxidation process, followed by a series of condensation and oxidation processes that render the eight-membered heterocycles **705**.

**Scheme 92:**
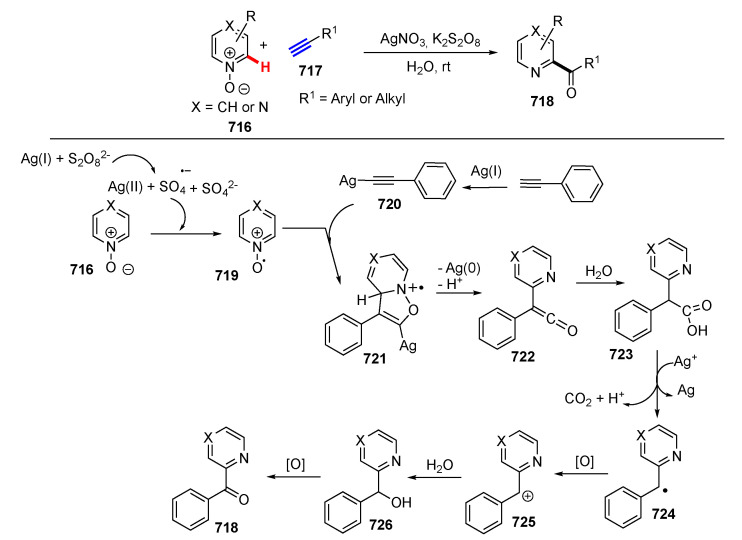
Radical acylation of electron-deficient heteroarenes.

Singh and collaborators described the oxidative acylation of electron-deficient heteroarenes **716** utilizing alkynes as acylating reagents (Scheme 92) [188]. The procedure employed AgNO_3_ as catalyst and K_2_S_2_O_8_ as oxidant. The reaction would be triggered by the generation of the *N*-oxide radical **719** upon removal of one electron from **716** by persulfate species. Next, the addition of this to the silver alkynyl species **720** formed in situ, would evolve to produce the ketene intermediate **722**. The hydration of **722** leads to the acid derivative **723**, which undergoes a silver-catalyzed decarboxylation to render the radical intermediate **724**. Further oxidation and hydration afford the acylated heterocycles **718**.

**Scheme 93:**
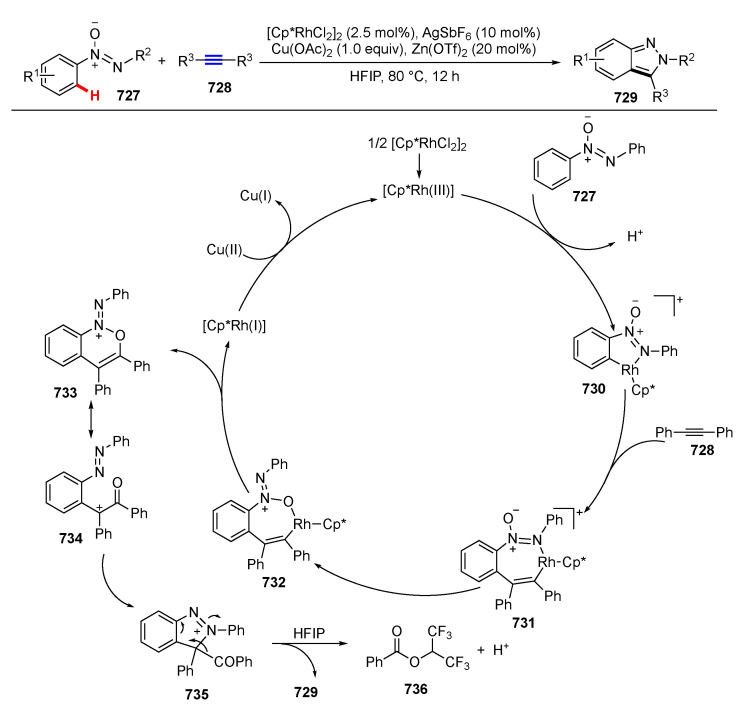
Synthesis of 2*H*-indazole via C–H/C–C cleavage. Cp* refers to pentamethylcyclopentadienyl ligand.

The You group reported a Rh-catalyzed route to 2*H*-indazoles **729** consisting in the [4 + 1]-annulation of azoxy compounds **727** with alkynes (Scheme 93) [189]. The reaction would commence with the azoxy-directed C–H metalation to give the rhodacycle **730**. Next, migratory insertion of the alkyne and reductive elimination of Rh(I) with C–O bond formation would give **733**. This intermediate would further evolve through ring-opening and reaction with HFIP to furnish the 2*H*-indazole scaffold.

## 5. Summary and Conclusions

The field of C–H activation and functionalization keeps on being a main avenue of research that, in conjunction with C–C bond cleavage, is giving rise to a great extension of synthetic tools to achieve complex organic transformations. This fact can be appreciated in the increasing number of works dealing with this type of methodology reported in the past few years. The combination of both types of bond functionalization can be assessed through different approaches, from classical transition-metal catalysis to more recent photocatalysis involving radical intermediates. Hence, given the versatility in terms of methodology along with the variability of substrate design, it would be expected that the C–H/C–C functionalization area will continue on attracting the interest of synthetic chemists in the years to come. Surely, new developments relaying on these new reaction manifolds will find applications in several areas of organic chemistry such as the synthesis of heterocyclic, pharmaceutical and natural products or materials science.

Some of the research areas where there are still limitations and room for improvement in the C–H/C–C bond functionalization field are for instance: (a) the deeper insights into the design of transition-metal catalyzed reactions where no directing groups need to be attached to the substrates, since usually the installation/removal of these groups make the overall synthetic processes more costly; (b) further implementation of electrochemical methods to avoid the use of stoichiometric oxidants/reductants and therefore contribute to the development of greener synthetic processes; or (c) the broadening of the routes to tackle the challenging cleavage of unstrained Csp^3^–Csp^3^ bonds, which have a ubiquitous presence in organic compounds, hence opening up new disconnections and possibilities in organic synthesis.
